# Multimodal antibacterial potency of newly designed and synthesized Schiff's/Mannich based coumarin derivatives: potential inhibitors of bacterial DNA gyrase and biofilm production†

**DOI:** 10.1039/d4ra05756b

**Published:** 2024-10-07

**Authors:** Kakarla Pakeeraiah, Pragyan Paramita Swain, Alaka Sahoo, Preetesh Kumar Panda, Monalisa Mahapatra, Suvadeep Mal, Rajesh Kumar Sahoo, Pratap Kumar Sahu, Sudhir Kumar Paidesetty

**Affiliations:** a Medicinal Chemistry Research Laboratory, School of Pharmaceutical Sciences, Siksha ‘O’ Anusandhan Deemed to be University Bhubaneswar 751003 Odisha India psudhirkumar@soa.ac.in; b Centre for Biotechnology, School of Pharmaceutical Sciences, Siksha ‘O’ Anusandhan Deemed to be University Bhubaneswar 751003 Odisha India; c Department of Skin & VD, Institute of Medical Sciences, SUM Hospital, Siksha ‘O’ Anusandhan Deemed to be University Bhubaneswar 751003 Odisha India; d Research and Development Division, Salixiras Research Private Limited Bhubaneswar Odisha India; e Department of Pharmacology, School of Pharmaceutical Sciences, Siksha ‘O’ Anusandhan Deemed to be University Bhubaneswar 751003 Odisha India pratapsahu@soa.ac.in

## Abstract

The briskened urge to develop potential antibacterial candidates against multidrug-resistant pathogens has motivated the present research study. Herein, newly synthesized coumarin derivatives with azomethine and amino-methylated as the functional groups have been focused on their antibacterial efficacy. The study proposed two distinct series: 3-acetyl substituted coumarin derivatives, followed by the Schiff base approach (5a–5i), and formaldehyde-secondary cyclic amine-based derivatives (7a–7g), using the Mannich base approach, further the compounds have been confirmed through various spectral studies. Further, target-specific binding affinity has been affirmed *via in silico* study. *In vitro* antibacterial study suggested compounds 5d and 5f to be most effective against *S. aureus* and multidrug-resistant *K. pneumoniae*, with MIC values of 8 and 16 μg mL^−1^. Among them, the compounds 5d and 5f showed excellent binding scores against different bacterial gyrase compared to the standard novobiocin. Based on RMRS, RMSF, Rg, and H-bond plots, MD simulation study at 100 ns also suggested better stability of 5d inside gyraseB of *E. coli* than the complex of *E. coli*-GyrB-novobiocin. The toxicity and pharmacokinetic profiles showed favorable drug-likeness. Overall, systematic *in vitro* and *in silico* assessment suggested that multimodal antibacterial derivatives 5d and 5f strongly inhibit both bacterial DNA gyrase and biofilm formation of drug-resistant pathogens, suggesting their potency in mainstream antibacterial therapy.

## Abstract 2

The briskened urge to develop potential antibacterial candidates against multidrug-resistant pathogens has motivated the present research study.

## Graphical abstract

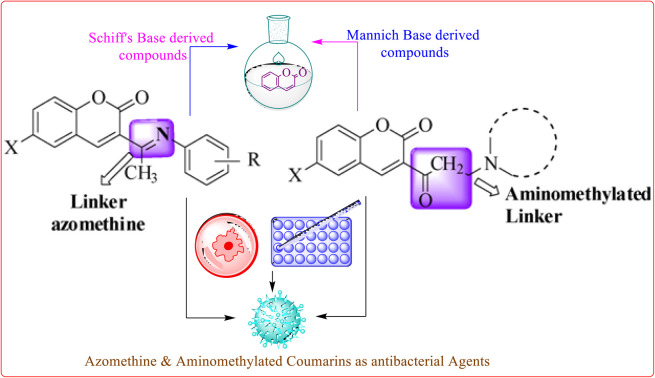

## 1. Introduction

Bacterial infections pose substantial health risks worldwide; currently, leading to heightened rates of illness and death. Pathogenic bacteria, especially multidrug-resistant (MDR) strains, are increasingly adept at resisting most modern antibacterial treatments. This resistance often emerges from shared resistances among bacteria naturally present in the human body and surrounding environments. As a result, there is a pressing need to develop novel drug candidates capable of combating the wide range of MDR bacterial strains.^1^ Many antibiotic classes including lactams, macrolides, tetracyclines, and fluoroquinolones have contributed significantly during the 20^'^s era in the treatment of bacterial infections, however, antimicrobial resistance (AMR) have risked the pharmacological actions of antibiotics imposing risks to human health.^2–4^ Clinically, the most notorious strains *viz.*, MRSA (Methicillin-Resistant *Staphylococcus aureus*), VRSA (vancomycin-Resistant *Staphylococcus aureus*), and CRSA (Clindamycin Resistant *Staphylococcus aureus*) are the causative pathogen deluding the mechanism of contemporary medicine.^5^ These nosocomial pathogens protrude from the soft skin and tissues causing serious dermatitis as their severe side-effects.^3,6–12^ Biofilm resistance causes serious threats to human health majorly urinary tract infections, dental or gingival plaque, catheters, inflammation of the prosthetic organs, and bacterial vaginosis, which have been associated with the production of bacterial biofilms produced by some serious bacterial pathogens *viz*. *Proteus mirabilis*, *Escherichia coli, Klebsiella pneumoniae*, *Enterococcus faecalis*, *Staphylococcus aureus*, *Staphylococcus epidermidis*, *Streptococcus viridans*, and *Pseudomonas aeruginosa*.^13^ Several prevalent diseases, including urinary tract infections, dental or gingival plaque, catheters, inflammation of the prosthetic organs, and bacterial vaginosis, have been associated due to the production of bacterial biofilms.^14,15^ These bacterial actions and reactions, increases the thirst for developing new potent drug among the budding researcher to control, prevent, and overcome the resistant problems. The drug development process confers the rationality either by incorporating smaller functional chemical entities in an existing antibiotic or by conjugating active phytochemicals into the core of heterocycles through different modifications for enhancing their biological actions.^16^ The literature survey imposes an eye upon amino methylated modifications of phytochemicals (thymol, menthol, carvacrol, coumarin, and quercetin) due to their versatility in producing different biological actions. Focusing upon the amino-methylated modification, Mannich reaction have been a great substitution reaction by introducing amino-methyl group into the rings of phenols/enols, also to the α-active carbonyl compounds through electrophilic substitution reactions.^17^ Mannich base derivatives have been shown a wide range of pharmacological actions including antihypertensive, antiparasitic, antiviral, antibacterial, antifungal, and anticancer.^18,19^ Certain marketed antibiotics synthesized through mannich base reactions are Rolitetracyclin, Clomocyclin, Lymicycline. Along with the amino-methylated substitution, Schiff base (C=N) substitution has also grabbed the attention of the researchers due to its reaction feasibility and bioavailability of drugs containing the small imine functional group *viz.*, nifuroxazide as antibiotic and thiacetazone as antituberculosis drugs.^20,21^ The most abundant nuclei present in all phytochemicals are coumarins and their isomers flavones, chemically coumarins are benzene fused with an α-pyrone system known as 2*H*-chromen-2-one.^22,23^ The most effective antibiotics, novobiocin and chlorobiocin, have a structural residue called amnio-coumarin nucleus that inhibits bacterial DNA gyrase.^24,25^ The present research focuses on the condensation reaction, previously some similar research works have been reported through microwave-assisted synthesis of quinoxalone with coumarin *via* a hydrazone linkage and evaluated for their antimicrobial actions, whereas our work emphasizes on the modification of 3-acetylcoumarin *via* two linkers amino-methylated and azo-methylated groups.^26–28^ Another coumarin-Schiff base reaction being reported for notable antibacterial efficacy, among which the coumarin candidate conjugated with sulfamethoxazole at C-3 position showed good inhibition against *S. aureus*.^29^ The newly synthesized derivatives have been investigated for their binding affinity with bacterial target (DNA gyrase B targets) through molecular docking and the physicochemical and pharmacokinetic profiles have also been evaluated through Lipinski's Rule of Five, ADMET, and PASS prediction. Thereafter, the stability and free energy optimization (HOMO–LUMO) of the screened potent candidate was determined by molecular dynamics simulations and molecular orbital analysis, respectively. Eventually, all the compounds been evaluated for their biofilm inhibition efficacy and antimicrobial potency against some harmful bacterial strains.

## 2. Experimental

### 2.1 Materials and methods

All the necessary chemicals were utilized as AR grade and provided by Sigma-Aldrich and used without purification. The products were analyzed by ATR (JASCO FT/IR4600 Spectrophotometer), H^1^/C^13^ NMR (Bruker NMR 400 MHz) using trimethyl silane (TMS) as an internal standard, and chemical shifts are reported in terms of ppm, *δ* values. The Elico Melting Point device was used to measure the melting point (0 °C). The reaction mixture was monitored by thin-layer chromatography (TLC) using silica gel 60 F254-coated with an appropriate solvent system. The separation and isolation of mixture components were carried out in column chromatography in an *n*-hexane and ethyl acetate with an appropriate ratio. Mass spectroscopy (MS) was recorded on Shimadzu GC-MS and the sample purity was confirmed by HPLC system, Shimadzu – LC-2030C 3D with Prominence-I pump, Autosampler: PDA detector maintains a flow rate of 1 mL min^−1^ and the acetonitrile: acetic acid in Millipore water was chosen as mobile phase. The percentage of elemental analysis (C, H, N) was performed on the PerkinElmer 240 analyzer.

### 2.2 Molecular docking study

The entire computational investigation was carried out using the Linux-Ubuntu 20.04.6 platform using several bioinformatics software and tools.^30,31^ Initially, all designed coumarin derivatives were converted to a three-dimensional structure in 3D file format using Open Babel and optimized using Avogadro software to get reliable and accurate binding interactions. Simultaneously, according to our ligand structure, we have selected three bacterial DNA gyrase Bs of *E. coli* (PDB ID: 7P2M), *A. baumannii* (PDB ID: 7PQI), *S. aureus* (PDB ID: 5D7R), dihydropteroate synthase (DHPS) of *S. aureus* (PDB ID: 6CLU), and a biofilm-associated target enzyme of *K. pneumoniae*, FabG (PDB ID: 6T77). As a standard antibacterial, novobiocin^32^ was used in the docking study. Followed by standardized molecular docking studies with manually defined grid boxes within their active site residues, PyRx 0.8 and AutoDock 4.2 software were used for the virtual screening and docking study.^30,33^ Further, out of ten docking poses, the one with the lowest-generated docking or binding energy (kcal mol^−1^) is considered the most potential docking pose against the respective target. The protein-ligand interactions were studied with Discovery Studio Visualizer software.^34,35^

### 2.3 Molecular dynamics (MD) simulation

Towards further stability and kinetic behaviors study through MD-simulation, we have selected the docking complex of ‘EC_GyrB-BACPN’ as the most potential ligand based on docking score, along with ‘EC_GyrB-Novobiocin’ as the standard antibacterial to compare. We performed MD simulation for 100 ns using the GROMACS-2022 software using the AMBER99SB-ILDN force field.^36,37^ The ACPYPE server was used to generate the ligand topologies along with the TIP3P3 water-filled model during MD simulation. We added solvent molecules to the system, neutralized it by adding further Na^+^ ions, and further performed the energy minimization step using the 50 000 steepest descent method along with a Fourier grid at 1.2 nm computational load for the Particle Mesh Ewald (PME). Following the energy minimization step, both complexes underwent NVT and NPT equilibrations to achieve system equilibrium over a 100 ps time scale. After completing the final MD step, we used MD trajectories to calculate the RMSD (root mean square deviation) and RMSF (root mean square fluctuation) plots using gmxrms and RMSF, respectively, and further plotted individual energy plots of RMSD (both backbone protein and ligand), RMSF, Rg, and the number of H-bonds for analyses.^38,39^

### 2.4 Synthesis

#### 2.4.1 Synthesis of Schiff based coumarin congeners (5a–5i)

**Scheme 1:**
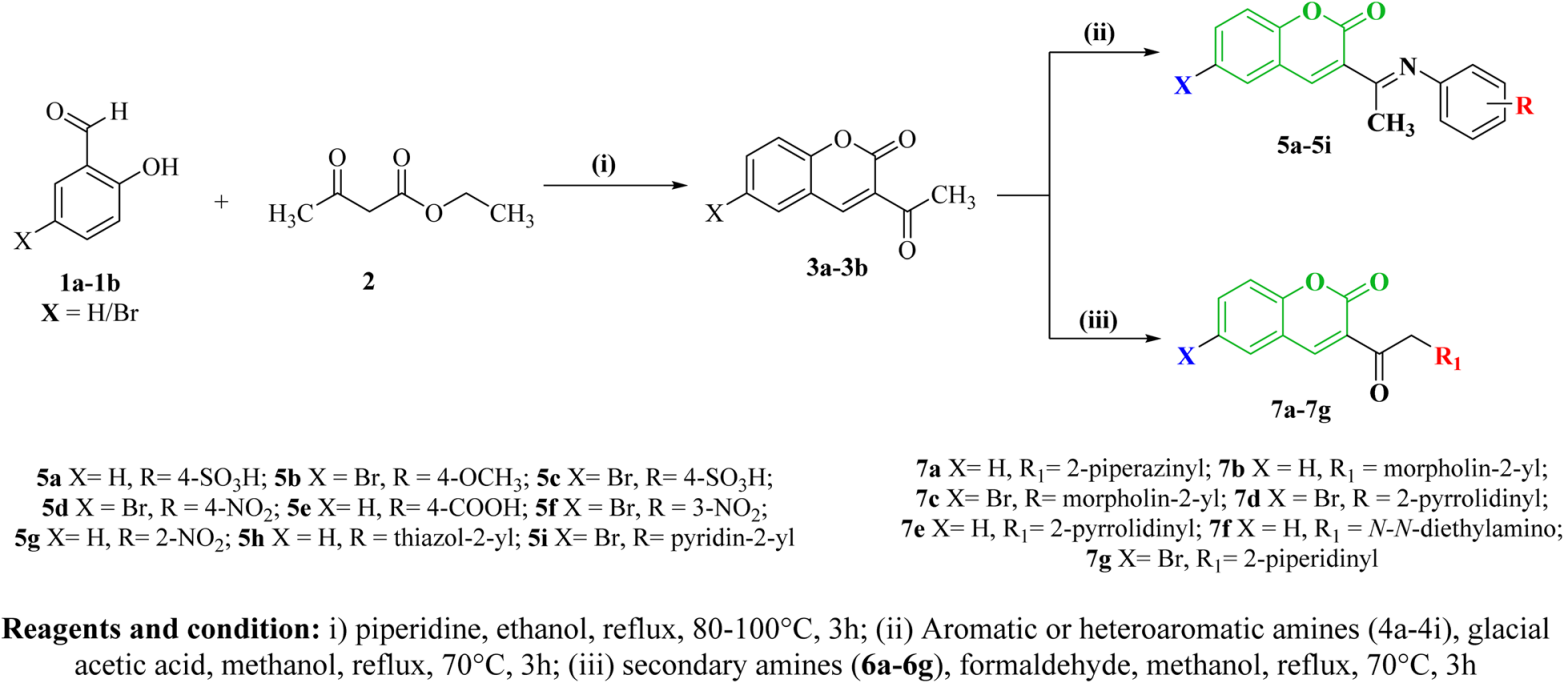


Equimolar concentration of individually substituted 3-acetyl coumarins (3a–3b) and aromatic amines (4a–4i) were stirred until completely dissolved in ethanol, followed by few drops of glacial acetic acid. The homogeneous mixture was refluxed at 100 °C for 2–4 h to obtain 5a–5i (Scheme 1). The reaction progress was monitored *via* TLC using ethyl acetate/cyclohexane solvent system. After completion of the reaction, the mixture was poured into ice-cold water, to obtain the precipitate, followed by filtration, and recrystallization using hot ethanol.^23^

#### 2.4.2. Synthesis of Mannich-based coumarin congeners (7a–7g)

A methanolic solution containing equimolar concentrations of substituted coumarins (3a–3b), formaldehyde, and various individual secondary amines (6a–6g), was refluxed about 3–5 h at 80 °C (Scheme 1). The progression of the reaction was monitored using TLC with solvent system containing ethyl acetate/cyclohexane. Upon completion of reaction, the reaction mixture was cooled and poured into ice-cold water, then left overnight in the refrigerator. The resulting solid mass was filtered, dried with anhydrous calcium chloride, and subjected to recrystallization from ethanol.^40^

### 2.5 Antimicrobial activity

#### 2.5.1. Agar well diffusion method

The antibacterial activity of both series of synthesized coumarin congeners, 5a–5i, and 7a–7g, was assessed using the Agar well diffusion method against the *S. aureus* and the MDR *Klebsiella pneumoanie* strains. Before screening, the individual bacterial strains were cultured in Muller–Hinton broth (MHB) and incubated overnight at 37 °C. The culture (0.5 McFarland standard) was spread onto sterilized Muller–Hinton Agar (MHA) Petri plates. Each aseptic well was loaded with 80 μL of test samples, previously dissolved in DMSO solvent at a concentration of 100 μg mL^−1^. *E. coli* (ATCC2592) was used as the control strain and ciprofloxacin antibiotic served as the positive control, while dimethyl sulfoxide (DMSO) solvent was used as the negative control. Subsequently, each plate containing the bacterial isolate was incubated for 24 hours at 37 °C ± 2 °C. Following incubation, the zone of inhibition was measured in terms of millimeters (mm) scale.^23^

#### 2.5.2. Microbroth dilution method

The Minimum Inhibitory Concentration (MIC) of the newly developed compound was determined using the microdilution method with 96 well micro-titreplate (flat bottom; polystyrene, Eppendorf). Following the screening of the zone of inhibition, the potent antibacterial candidates 5d and 5f were further evaluated for their MIC values against *S. aureus* and MDR *K. pneumoniae* (KPATCC13883). *E. coli* (ATCC25922) was used as the control strain, and ciprofloxacin antibiotic was used as standard. The serial dilution experiment commenced by dispensing a 100 μL aliquot of the test sample from its stock solution (1024 μg mL^−1^ in 10% DMSO) into the first well containing 100 μL of media (MHB), establishing the highest concentration of the sample (512 μg mL^−1^). Subsequently, successive dilutions were performed by transferring the solution from the first well to the eleventh well and the aliquot (100 μL from the eleventh well was discarded) (1 μg mL^−1^). The twelfth well in the 96-well titer plate did not contain the test sample serving as control following this, 100 μL of the inoculum (10^7^ CFU mL^−1^) was added to each well and incubated at 37 °C for 24 h. The MIC of the test samples was determined by observing the colour change in the wells, indicative of bacterial growth inhibition after the addition of 5 μL of indicator (2, 3, 5-triphenyl tetrazolium chloride, 0.5%).^41,42^

#### 2.5.3. Scanning electron microscopy for observing antibiofilm activity

The antibiofilm activity of the compound was evaluated using scanning electron microscopy (SEM). A sterile glass coverslip (1 × 1 cm) was inserted into each tube containing 5 mL of MDR *K. pneumoniae* isolate culture (∼1 × 10^6^ CFU mL^−1^) grown overnight in LB broth. The tubes were then incubated horizontally at 37 °C for 72 h statically in the presence and absence of the compound (5d & 5f). The *Klebsiella pneumonia* KPATCC 13883 was taken as a control strain to check the antibiofilm activity of compounds 5d & 5f. The coverslips were then washed in 0.1 M phosphate-buffered saline (PBS) and fixed for 24 hours at room temperature with 2.5% glutaraldehyde prepared in 0.1 M PBS. Following fixation, the coverslips were cleaned with 0.1 M PBS, air-dried, and subjected to a graded series of dehydration in ethanol solution (10%, 30%, 50%, 70%, 80%, 90%, and absolute ethanol) for 15 minutes each. Subsequently, the materials were subjected to scanning electron microscopy (SEM) using an EVO® 18 (Carl Zeiss, Germany).^43^

## 3. Results and discussion

### 3.1 Chemistry

Two series of coumarin derivatives (5a–5i) and (7a–7g), based on Schiff base and Mannich reactions, were virtually designed using pharmacophore modeling guided by organic reaction principles. These compounds are anticipated to possess potential antibacterial properties, targeting specific bacterial sites. Specifically, our selection of bacterial targets aligns with the known activity of coumarin-based antibiotics like Novobiocin, which inhibits bacterial DNA gyrase. Consequently, these designed candidates are anticipated to act on the DNA gyrase target. Initially, all the candidates are subjected to docking and MD simulations with DNA gyrase target of various bacterial strains. From these simulations, the most promising candidates were selected based on their molecular docking scores with the respective targets of bacterial strains. Subsequently, here in current study, a retrosynthetic analysis of the potent docked candidates was executed. The docking scores unveiled that sixteen compounds demonstrated enhanced binding affinity with the targets. Remarkably, among the coumarinyl Schiff's bases, compounds 5d and 5f featuring 4-nitrophenyl and 3-nitrophenyl conjugated to 6-bromo-3-acetyl-coumarin *via* an azomethine linker, displayed the best binding energy scores of −8.4 and −8.7 kcal mol^−1^ against Gram-negative bacterial DNA gyrase B respectively. Meanwhile, in the Mannich base series, compounds 7d and 7g showcased the highest docking scores of −7.6 and −8.8 kcal mol^−1^ with *K. pneumoniae* and *E. coli* bacterial DNA gyraseB. These compounds, chemically substituted with pyrrolidinomethylated and piperidinylmethylate groups at the α-carbon of acetyl coumarin, demonstrated better binding interactions with bacterial DNAgyrase (Table 3).

The schematic synthesis is depicted in Scheme 1. Coumarinyl-Schiff's bases (5a–5i) were synthesized through the condensation of aromatic primary amines (4a–4i) with derivatives containing an active methyl group, such as 3-acetyl-coumarin (3a–3b), under mild conditions *via* a nucleophilic addition reaction.^43^ Similarly, Mannich base conjugated coumarins were synthesized by mixing derivatives of 3-acetyl-coumarin (3a–3b) with formaldehyde and the respective secondary amines (6a–6g), preferably in hydrochloride salt form.^44^ An intermediate 3-acetyl-coumarin (3a–3b) was synthesized by base catalyzed cyclic condensation of corresponding salicylaldehyde (1a–1b) with ethyl acetoacetate 2.^45^ Additionally, the physicochemical parameters of these sixteen compounds were assessed according to Lipinski's rule of five. It was observed that all the compounds met the criteria and remained within their respective limits in terms of hydrogen acceptor count, hydrogen donor count, total polar surface area, octanol/water coefficient, and molecular weight.^46^ The results of both physiochemical parameters and molecular docking are illustrated in Tables 1 & 3. The prediction of the pharmacokinetic profile of our designed compounds was performed by pre-ADMET and their results are depicted in Table 1. The ADMET profiles encompass various parameters *viz*. blood–brain barrier (BBB), Caco-2 permeability (the Caco-2 cells for the expectation of oral medication retention technique), human digestive retention (amount of bioavailability and ingestion), skin penetrability and toxicity LD_50_. These predicted data suggested that coumarinyl candidates came under toxicity classes 4 and 5 that indicates for the ideal lead candidates which further evaluation an *in vitro* investigation. The obtained compounds underwent structural confirmation through various spectral studies, encompassing the detection of functional groups by FTIR spectroscopy, analysis of the hydrocarbon skeleton environment *via* NMR spectroscopy, determination of molecular weight through fragmented base ion peak detected by mass spectrometry, and calculation of elemental percentages to ascertain empirical formulas using elemental analysis.

**Table 1:** ADME prediction of the newly synthesized coumarin congeners 5a–5i & 7a–7g

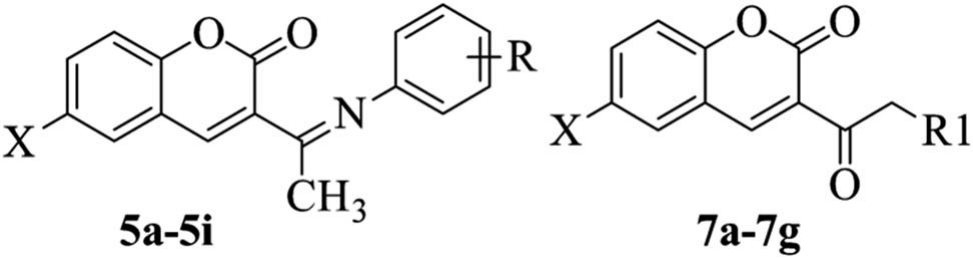							
Compound	R/R_1_	BBB	Caco2	HIA	Skin per	LD_50_ (mg kg^−1^)	Toxicity class
5a	4-SO_3_H	0.07666	0.47667	98.6464	−1.8887	1000	4
5b	4-OCH_3_	0.41541	43.8391	97.5053	−2.9894	1000	4
5c	2-NO_2_	0.03102	18.1869	99.1931	−2.8492	1000	4
5d	4-NO_2_	0.18618	18.3579	99.1931	−2.8727	1000	4
5e	4-COOH	1.06189	20.2941	98.8184	−3.1568	1000	4
5f	3-NO_2_	0.05879	18.3392	99.1931	−2.8712	1000	4
5g	2-NO_2_	0.30933	21.4908	98.0185	−2.9571	1000	4
5h	Thiazol-2yl	0.22991	5.13507	96.1816	−3.9691	5000	5
5i	Pyridin-2-yl	0.52272	14.0585	97.2938	−3.6295	1000	4
7a	Piperazinyl	0.02869	15.8007	92.9511	−4.3108	2500	5
7b	Morpholin-2-yl	0.1304	50.5753	98.2016	−3.9345	2500	5
7c	Morpholin-4-yl	0.04542	33.6558	97.4843	−3.8893	2500	5
7d	Pyrrolidinyl	0.28157	28.3168	95.9774	−3.8131	2500	5
7e	Pyrrolidinyl	0.07742	21.034	92.1695	−4.3119	2500	5
7f	*N*,*N*-Diethylamino	0.02231	45.7292	98.1587	−3.3982	5000	5
7g	Piperidinyl	0.40059	31.4439	96.0452	−3.6701	2500	5

### 3.2 Spectral analysis

FT-IR spectral data were analyzed to provide additional data regarding the functional groups present in the desired structure. Across all compounds, specific frequencies appeared in the FT-IR at around 1737 cm^−1^, indicating the presence of carbonyl pyrone functionality, and in the range of 1623–1604 cm^−1^, indicative of unsaturated olefin functionality at the 3,4 positions within the coumarin ring system. In the Schiff-base series the compounds 5a–5i, the frequencies at a range between 1675–1646, and 2952–2930 cm^−1^ were contributed for the stretching vibrations of azomethine and methylene respectively. All the compounds appeared to moderate peaks at a range of 1275–1265 cm^−1^ nearly, which is associated with ether C–O stretching of the pyrone lactone system.

In compound 5d, the absorption bands were observed at 1395 and 1180 cm^−1^, indicative of the presence of the nitro group within the structure. Additionally, an absorption band was noted at 1285 cm^−1^, corresponding to the C–O stretching vibration. Initially, the FT-IR spectrum of 3-acetyl coumarin revealed two carbonyl stretching bands. Following the reaction, one of the carbonyl bands disappeared, giving rise to a new absorption peak associated with the formation of a Schiff base azomethine group. This structural transformation was further confirmed through analysis of both FT-IR and ^13^C NMR spectra. The compound 5f which contains a nitro group in the *meta* position herein distinguished from compound 5d with para-nitro in terms of frequencies appeared in FT-IR at 857 and 880 cm^−1^. In compound 5a, the FTIR spectra revealed distinct absorption peaks corresponding to the asymmetrical and symmetrical stretching vibrations of the sulfonyl group. These vibrations were observed at frequencies of 1363 cm^−1^ and 1158 cm^−1^, respectively. In the series of Mannich bases 7a–7g, all compounds underwent aminomethylation at the α-carbon of the acetyl group of coumarin. In this context, two distinct carbonyl stretching bands were identified through FTIR analysis. Specifically, the stretching vibrations were observed within the ranges of 1733–1715 cm^−1^ and 1676–1669 cm^−1^, assigned to the carbonyl functionalities of the pyrone and the free carbonyl of the acetyl ketone system, respectively. The hydrocarbon skeletons of all the targeted Schiff's or Mannich bases was verified through Nuclear Magnetic Resonance (NMR) spectroscopy. Specifically, in all Schiff's bases, de-shielding singlet protons were observed at approximately *δ* 2.54 ppm, attributable to the acetyl methyl protons. Furthermore, in the ^13^C NMR spectra, the methyl carbon resonated at around *δ* 19.01 ppm across all the confirmed structures. In addition, compound 5b exhibited sharp singlet signals at approximately *δ* 3.83 ppm, which can be attributed to the methyl protons of the methoxy group and the same carbon reflected in ^13^C NMR at *δ* 40.627 ppm. In all synthesized compounds, the pyrone carbonyl peak was reflected at *δ* 158.234 ppm. The Schiff's base congeners derived from 6-bromo-3-acetyl-coumarin 2b, namely 5b, 5c, 5d, 5f, and 5i, displayed sharp aromatic shielded singlet signals in the region of approximately *δ* 8.06–8.64 ppm and *δ* 7.76–7.86 ppm. These signals correspond to the protons at the 4th and 5th positions of the coumarin ring. In compounds 5a–5i, the carbon of the azomethine group was observed within a range of *δ* 158.454–161.084 ppm in the ^13^C NMR spectra. The compounds 5a–5i and 7a–7g have been showing sharp singlet signals at nearly equal to *δ* 3.59 ppm assigning to methyl protons, which in ^13^C NMR, reflected at *δ* 19.01 ppm in all the above-confirmed structures. The compounds Schiff's-base coumarin congeners (5a–5i) appeared at a broad singlet signal in the region ∼*δ* 7.4–8.5 ppm which is indicated as 4th position hydrogen of coumarin nucleus whereas proton information for the compounds 7a–7g has been specifying for singlet aromatic proton peak at *δ* 7.93 and *δ* 7.67 ppm respectively, whereas the compounds displaying another singlet peak at ∼*δ* 8.6 ppm concerning coumarinyl H-4 proton whose parallel carbon peak appeared at ∼*δ* 133.77 ppm. The Mannich base 7a–7g contains two aromatic protons in the region between *δ* 7.46–7.95 ppm, whereas the bromo-substituted derivatives 5d & 5f exhibit signals for the fourteen aromatic protons in the region between *δ* 7.24–7.66 ppm. The relevant spectra of all obtained synthesized compounds are depicted in ESI Fig. 5a–5i and 7a–7g.† Moreover, the synthesized molecules have not yet been reported earlier. All the compounds' electronic spectra were scanned in methanol and displayed bands that are assigned for π–π* and n–π* transitions state at 285 nm and 315 nm respectively. The molecular weight of all the synthesized compounds was determined by Electrospray Ionization-Mass spectroscopy (ESI-HRMS) in terms of *m*/*z* value. The ESI-HRMS spectra of the prepared compounds (5a–5g) and (7a–7i) revealed the highest fragmented ion peaks strongly assigned to their prediction molecular formulae. The compound 5d had shown a molecular ion peak at 386.68, which strongly indicated the predicted molecular formula for C_17_H_11_BrN_2_O_4_. From the HPLC chromatograms, it has been noticed that the compound 5d is highly pure by a percentage area of 100% with a retention time of 17.325 min; and the Inertsil ODS-3 (C-18) analytical column was used with a 55 : 45 v/v ratio of HPLC grade water and acetonitrile. The chromatogram has been depicted in supplementary Fig. S68.†

### 3.3 Antimicrobial assessment

The majority of the derivatives displayed varying degrees of antibacterial efficacy against Gram-positive bacteria, including *Klebsiella pneumoniae*, and *Staphylococcus aureus*, with *Escherichia coli* used as a standard. However, they also exhibited a range of responses from moderate to resistant effects specifically against *K. pneumoniae* and *S. aureus*. All the synthesized coumarin congeners 5a–5i, and 7a–7g have been shown moderate to good zone of inhibition the compounds that show maximum zone of inhibition. Furthermore, the most potential compounds were performed determination of their MIC against the strains mentioned above. The zone of inhibition of compounds 5d was found at 24, 24, and 23 mm against *S. aureus* and MDR *Klebsiella pneumonia* and *E. coli* respectively whereas the 5f had shown 25, 22, and 22 mm and also comparable to standard Ciprofloxacin and Novobiocin. The compound 5d had shown their respective MIC values 4, 4 and 1 μg mL^−1^ similarly, another analog 5f had shown their respective MIC 4, 8 and 1 μg mL^−1^ against *S. aureus* and MDR *Klebsiella*. Next potent candidate 5b had shown their inhibitory concentrations at 16 and 32 μg mL^−1^ against both *S. aureus* and MDR *Klebsiella pneumoniae* respectively. Structure activity relationship (SARs) of the synthesized compounds suggested that the compounds with substituted nitro groups attached to the phenyl ring at para or meta positions linked with 6-bromo-3-acetyl-coumarin showed enhanced antibacterial activity. *Ortho* nitrophenyl attached to unsubstituted 3-acetyl coumarin *via* linkage of azomethine exhibited moderate activity against *S. aureus* but good activity against MDR *Klebsiella. pneumoniae*. Schiff's base coumarins were generally more potent than Mannich bases derived coumarins. Compound 5e showed resistance against all tested strains. The results of the tested compounds are depicted in Table 4.

### 3.4 Spectral characterization of Schiff's base and mannich base coumarin congeners

#### 3.4.1. 4-((1-(2-Oxo-2*H*-chromen-3-yl) ethylidene) amino) benzenesulfonic acid (5a)

Condensation of an equimolar concentration of ethanolic solution of 3-acetyl coumarin with sulfanilic acid obtained precipitate mass in yellowish white powder; yield: 78%; white powder; UV-visible (*λ*_max_, CH_3_OH): 306 nm; IR (ATR, *γ*, cm^−1^): 2827 (CH_2_ str.), 1732 (C=O str.), 1665 (C=N str.), 1604 (C=C str.), 1556 (CH-Ar str.), 1358 1192 (SO_2_ str.), 1284 (C–O str.), 827 755 (1,4-disubt. Ar); ^1^H NMR (DMSO-*d*_6_*δ*ppm, 400 MHz); 7.41–7.93 (d, 2H, pheny lH), 8.64 (s, 1H, coumarinyl H-4), 7.95 (d, 1H, coumarinyl H-5, *J* = 1.6 Hz), 7.72 (t, 1H, coumarinyl H-6, *J* = 8.14 Hz), 7.74 (t, 1H, coumarinyl H-7, *J* = 8.85 Hz), 7.95 (d, 1H, coumarinyl H-8, *J* = 10 Hz), 3.41 (s, 1H, SO_3_H), 2.58 (s, 3H, CH̲_3_); ^13^C NMR (400 MHz, DMSO-*d*_6_); 195.53, 158.88, 155.06, 147.50, 134.93, 131.23, 131.23, 127.42, 127.34, 125.39, 125.38, 124.87, 124.67, 121.36, 118.61, 116.56, 23.06; analysis for C_17_H_13_NO_2_S; calcd%: C, 69.13; H, 4.44; N, 4.74; S, 10.85; found%: C, 69.84; H, 3.72; N, 4.56. S, 10.55; ESI-HRMS (*m*/*z*): anal. calcd. for C_17_H_13_NO_2_S [M + H]^+^ 295.89; found: 296.05 (M + 1).

#### 3.4.2. 6-Bromo-3-(1-((2-methoxyphenyl)imino)ethyl)-2*H*-chromen-2-one (5b)

Condensation of an equimolar concentration of ethanolic solution of 6-bromo-3-acetyl coumarin with 2-anisidine obtained precipitate mass in light greenish powder; yield: 78%; white powder; UV-visible (*λ*_max_, CH_3_OH): 308 nm; IR (ATR, *γ*, cm^−1^): 3006 (Ar CH str.), 2831 (CH_2_ str.), 1731 (C=O str.), 1675 (C=N str.), 1607 (C=C str.), 1557 (CH Ar), 1291 (C–O str.), 827 (1,2-disubt. Ar); ^1^H NMR (DMSO-*d*_6_ δppm, 400 MHz); 7.09–7.96 (m, 4H, pheny lH), 8.66 (s, 1H, coumarinyl H-4), 7.96 (s, 1H, coumarinyl H-5), 7.94 (*d*, 1H, coumarinyl H-7, *J* = 8.85 Hz), 7.74 (d, 1H, coumarinyl H-8, *J* = 10 Hz), 3.69 (s, 1H, OCH_3_), 2.51 (s, 3H, CH̲_3_); ^13^C NMR (400 MHz, DMSO-*d*_6_); 195.56, 158.90, 155.08, 147.51, 134.94, 131.24, 126.59, 125.39, 124.90, 123.08, 121.76, 121.57, 118.63, 116.93, 116.57, 115.54, 115.14, 115.04, 114.93, 114.26, 40.41, 27.25; analysis for C_18_H_14_BrNO_3_; calcd%: C, 58.03; H, 3.74; N, 3.76; Br, 21.37; found%: C, 58.84; H, 4.02; N, 3.66. Br, 20.92; ESI-HRMS (*m*/*z*): anal. calcd. for C_18_H_14_BrNO_3_ [M + H]+371.26; found: 372.05 (M + 1).

#### 3.4.3. 6-Bromo-3-(1-((2-nitrophenyl)imino)ethyl)-2*H*-chromen-2-one (5c)

Condensation of an equimolar concentration of ethanolic solution of 6-bromo-3-acetyl coumarin with 2-nitroaniline obtained precipitate mass in yellowish white powder; yield: 78%; white powder; UV-visible (*λ*_max_, CH_3_OH): 290 nm; IR (ATR, *γ*, cm^−1^): 3029 (Ar CH str.), 2926 (CH_2_ str.), 1738 (C=O str.), 1674 (C=N str.), 1611 (C=C str.), 1554 (CH Ar), 1353, 1158 (SO_2_ str.), 1264 (C–O str.), 879 (1,4-disubt. Ar); ^1^H NMR (DMSO-*d*_6_*δ*ppm, 400 MHz); 7.22–7.66 (m, 4H, pheny lH), 8.66 (s, 1H, coumarinyl H-4), 7.95 (s, 1H, coumarinyl H-5), 7.97 (d, 1H, coumarinyl H-7, *J* = 8.88 Hz), 7.48 (d, 1H, coumarinyl H-8, *J* = 8.87 Hz), 2.51 (s, 3H, CH̲_3_); ^13^C NMR (400 MHz, DMSO-*D*_6_); 195.58, 158.90, 155.08, 147.51, 134.95, 131.24, 127.52, 125.41, 124.92, 121.73, 118.64, 116.5830.50; analysis for C_17_H_11_BrN_2_O_4_; calcd%: C, 52.84; H, 3.02; N, 7.26; Br, 20.62; found%: C, 52.62; H, 2.98; N, 7.16. Br, 20.55; ESI-HRMS (*m*/*z*): anal. calcd. for C_17_H_11_BrN_2_O_4_ [M + H]^+^ 385.89; found: 386.05 (M + 1).

#### 3.4.4. 6-Bromo-3-(1-((4-nitrophenyl)imino)ethyl)-2*H*-chromen-2-one (5d)

Condensation of an equimolar concentration of ethanolic solution of 6-bromo-3-acetyl coumarin with 4-nitroaniline obtained precipitate mass in creamish white powder; yield: 78%; white powder; UV-visible (*λ*_max_, CH_3_OH): 275 nm; IR (ATR, *γ*, cm^−1^): 3354 (ArCHstr.), 2884 (CH_2_ str.), 1733 (C=O str.), 1646 (C=N str.), 1627 (C=C str.), 1582 (CH Ar), 1395, 1180 (NO_2_ str.), 1285 (C–O str.), 880 (1,4-disubt. Ar); ^1^H NMR (DMSO-*d*_6_*δ*ppm, 400 MHz); 6.96–7.86 (m, 4H, pheny lH), 8.16 (s, 1H, coumarinyl H-4), 7.73 (s, 1H, coumarinyl H-5), 7.66 (d, 1H, coumarinyl H-7, *J* = 8.88 Hz), 7.64 (d, 1H, coumarinyl H-8, *J* = 8.87 Hz), 2.68 (s, 3H, CH̲_3_); ^13^C NMR (400 MHz, DMSO-*D*_6_); 170.95, 160.56, 156.16, 138.39, 136.11, 132.60, 126.84, 120.11, 115.63, 112.83, 110.30, 19.00; analysis for C_17_H_11_BrN_2_O_4_; calcd%: C, 52.64; H, 3.02; N, 7.26; Br, 20.62; found%: C, 52.62; H, 2.98; N, 7.15. Br, 20.45; ESI-HRMS (*m*/*z*): anal. calcd. for C_17_H_11_BrN_2_O_4_ [M + H]^+^ 385.79; found: 386.65 (M + 1).

#### 3.4.5. 4-(((2-Oxo-2*H*-chromen-3-yl)methylene)amino)benzoic acid (5e)

Condensation of an equimolar concentration of ethanolic solution of 3-acetyl coumarin with 4-aminobenzoic acid obtained precipitate mass in creamish white powder; yield: 78%; white powder; UV-visible (*λ*_max_, CH_3_OH): 289 nm; IR (ATR, *γ*, cm^−1^): 3029 (Ar CH str.), 2852 (CH_2_ str.), 1738 (C=O str.), 1675 (C=O str.), 1611 (C=N str.), 1605 (C=C str.), 1556 (CH Ar), 1264 (C–O str.), 855 (1,4-disubt. Ar.); ^1^H NMR (DMSO-*d*_6_*δ*ppm, 400 MHz); 11.20 (s, 1H, OH), 7.61–7.93 (m, 4H, pheny lH), 8.64 (s, 1H, coumarinyl H-4), 7.95 (s, 1H, coumarinyl H-5), 7.74 (m, 1H, coumarinyl H-6, *J* = 8.88 Hz), 7.62 (m, 1H, coumarinyl H-7, *J* = 8.88 Hz), 7.39 (d, 1H, coumarinyl H-8, *J* = 8.87 Hz), 2.51 (s, 3H, CH̲_3_); ^13^C NMR (400 MHz, DMSO-*D*_6_); 195.54, 158.89, 155.06, 147.50, 134.93, 131.67, 131.23, 125.39, 124.88, 118.62, 116.56, 113.02, 19.01; analysis for C_18_H_13_NO_4_; calcd.%: C, 70.34; H, 4.24; N, 4.66; found%: C, 69.82; H, 4.18; N, 4.15; ESI-HRMS (*m*/*z*): anal. calcd. for C_18_H_13_NO_4_ [M + H]^+^ 307.68; found: 308.65 (M + 1).

#### 3.4.6. 6-Bromo-3-(1-((3-nitrophenyl)imino)ethyl)-2*H*-chromen-2-one (5f)

Condensation of an equimolar concentration of ethanolic solution of 6-bromo-3-acetyl coumarin with 3-nitroaniline obtained precipitate mass in yellowish white powder; yield: 78%; white powder; UV-visible (*λ*_max_, CH_3_OH): 297 nm; IR (ATR, *γ*, cm^−1^): 2817 (CH_2_ str.), 1731 (C=O str.), 1675 (C=N str.), 1603 (C=C str.), 1556 (CH Ar), 1354, 1180 (NO_2_ str.), 1233 (C–O str.), 857 (1,3-disubt. Ar.); ^1^H NMR (DMSO-*d*_6_*δ*ppm, 400 MHz); 7.27–7.86 (m, 4H, pheny lH), 8.07 (s, 1H, coumarinyl H-4), 7.64 (s, 1H, coumarinyl H-5), 7.72 (d, 1H, coumarinyl H-7, *J* = 8.88 Hz), 7.23 (d, 1H, coumarinyl H-8, *J* = 8.87 Hz), 2.51 (s, 3H, CH̲_3_); ^13^C NMR (400 MHz, DMSO-*D*_6_); 170. 99, 160.59, 150.52, 149.20, 138.33, 132.60, 130.33, 120.42, 120.06, 115.64, 110.27, 110.25, 107.53, 18.98; analysis for C_17_H_11_BrN_2_O_4_; calcd%: C, 52.62; H, 3.02; N, 7.16; Br, 20.62; found%: C, 52.52; H, 2.98; N, 7.19. Br, 20.45; ESI-HRMS (*m*/*z*): anal. calcd. for C_17_H_11_BrN_2_O_4_ [M + H]^+^ 385.79; found: 386.65 (M + 1).

#### 3.4.7. 3-(1-((2-Nitrophenyl)imino)ethyl)-2*H*-chromen-2-one (5g)

Condensation of an equimolar concentration of ethanolic solution of 3-acetyl coumarin with 2-nitroaniline obtained precipitate mass in yellowish white powder; yield: 78%; white powder; UV-visible (*λ*_max_, CH_3_OH): 297 nm; IR (ATR, *γ*, cm^−1^): 3029 (Ar CH str.), 2930 (CH_2_ str.), 1738 (C=O str.), 1674 (C=N str.), 1623 (C=C str.), 1556 (CH Ar), 1345, 1158 (NO_2_ str.), 1251 (C–O str.), 870 (1,2-disubt. Ar); ^1^H NMR (DMSO-*d*_6_*δ*ppm, 400 MHz); 7.04–7.96 (m, 4H, pheny lH), 8.67 (s, 1H, coumarinyl H-4), 7.75 (s, 1H, coumarinyl H-5), 7.39 (m, 1H, coumarinyl H-6), 7.46 (d, 1H, coumarinyl H-7, *J* = 8.88 Hz), 7.48 (d, 1H, coumarinyl H-8, *J* = 8.87 Hz), 2.54 (s, 3H, CH̲_3_); ^13^C NMR (400 MHz, DMSO-*D*_6_); 155.30, 147.77, 146.35, 136.23, 136.15, 131.29, 125.81, 125.42, 119.69, 119.32, 116.90, 115.91, 115.77, 19.00; analysis for C_17_H_12_N_2_O_4_; calcd%: C, 66.23; H, 3.92; N, 9.10; found%: C, 66.52; H, 4.18; N, 9.19.; ESI-HRMS (*m*/*z*): anal. calcd. for C_17_H_12_N_2_O_4_ [M + H]^+^ 308.79; found: 309.65 (M + 1).

#### 3.4.8. 3-(2-((4,5-Dihydrothiazol-5-yl)imino)acetyl)-2*H*-chromen-2-one (5h)

Condensation of an equimolar concentration of ethanolic solution of 3-acetyl coumarin with 2-aminthiazole obtained precipitate mass in yellowish white powder; yield: 78%; white powder; UV-visible (*λ*_max_, CH_3_OH): 287 nm; IR (ATR, *γ*, cm^−1^): 3029 (Ar CH str.), 2926 (CH_2_ str.), 1737 (C=O str.), 1674 (C=N str.), 1611 (C=C str.), 1554 (CH Ar), 1353, 1158 (SO_2_ str.), 1264 (C–O str.), 879 (1,4-disubt. Ar); ^1^H NMR (DMSO-*d*_6_*δ*ppm, 400 MHz); 8.66 (s, 1H, thiazole H-3), 7.96 (s, 1H, thiazole H-5), 7.94 (s, 1H, coumarinyl H-4), 7.77 (s, 1H, coumarinyl H-5), 7.75 (s, 1H, coumarinyl H-6), 7.73 (s, 1H, coumarinyl H-7), 7.48 (s, 1H, coumarinyl H-4), 7.42 (s, 1H, coumarinyl H-4), 7.40 (s, 1H, coumarinyl H-4), 2.51 (s, 3H, CH̲_3_); ^13^C NMR (400 MHz, DMSO-*D*_6_); 195.61, 158.91, 155.07, 147.51, 134.96, 131.24, 125.42, 134.93, 118.63, 116.58, 30.49; analysis for C_14_H_10_N_2_O_3_S; calcd%: C, 58.74; H, 3.55; N, 9.52; found%: C, 59.21; H, 4.11; N, 10.25; ESI-HRMS (*m*/*z*): anal. calcd. for C_14_H_10_N_2_O_3_S [M + H]^+^ 286.41; found: 287.35 (M + 1).

#### 3.4.9. 6-Bromo-3-(1-(pyridin-2-ylimino)ethyl)-2*H*-chromen-2-one (5i)

Condensation of an equimolar concentration of ethanolic solution of 6-bromo-3-acetyl coumarin with 2-aminopyridine obtained precipitate mass in yellowish white powder; yield: 78%; white powder; UV-visible (*λ*_max_, CH_3_OH): 306 nm; IR (ATR, *γ*, cm^−1^): 3029 (Ar CH str.), 2926 (CH_2_ str.), 1737 (C=O str.), 1674 (C=N str.), 1611 (C=C str.), 1554 (CH Ar), 1264 (C–O str.), 879 (1,4-disubt. Ar); ^1^H NMR (DMSO-*d*_6_ δppm, 400 MHz); 8.60 (s, 1H, coumarinyl H-4), 7.90 (s, 1H, coumarinyl H-5), 7.43 (s, 1H, coumarinyl H-6), 7.88 (s, 1H, coumarinyl H-7), 7.46 (s, 1H, coumarinyl H-8), 2.55 (s, 3H, CH̲_3_); ^13^C NMR (400 MHz, DMSO-*D*_6_); 195.45, 158.45, 154.09, 146.09, 137.05, 133.00, 125.91, 120.53, 118.88, 116.82, 40.89, 40.61, 40.40, 39.35, 30; analysis for C_15_H_16_N_2_O_3_; analysis for C_16_H_11_BrN_2_O_2_; calcd%: C, 56.21; H, 3.81; N, 8.14; found%: C, 57.41; H, 4.02; N, 9.22; ESI-HRMS (*m*/*z*): anal. calcd. for C_16_H_11_BrN_2_O_2_ [M + H]^+^ 342.80; found: 343.51 (M + 1).

#### 3.4.10. 3-(2-(Piperazin-2-yl)acetyl)-2*H*-chromen-2-one (7a)

Condensation of an equimolar concentration of methanolic solution of 3-acetyl-coumarin and piperazine with formaldehyde in presence of dilute HCl obtained yellowish whitepowder; yield: 78%; white powder; UV-visible (*λ*_max_, CH_3_OH): 286 nm; IR (ATR, *γ*, cm^−1^): 3382 (Ar CH str.), 2922 (CH_2_ str.), 1715 (C=O str.), 1673 (C=N str.), 1607 (C=C str.), 1551 (CH Ar), 1205 (C–N str.); ^1^H NMR (DMSO-*d*_6_*δ*ppm, 400 MHz); 8.65 (s, 1H, coumarinyl H-4), 7.96 (s, 1H, coumarinyl H-5), 7.42 (s, 1H, coumarinyl H-6), 7.75 (s, 1H, coumarinyl H-7), 7.47 (s, 1H, coumarinyl H-8), 2.55 (s, 3H, CH̲_3_); ^13^C NMR (400 MHz, DMSO-*D*_6_); 195.63, 158.90, 155.07, 147.50, 134.96, 131.23, 125.42, 124.94, 118.62, 116.58, 40.86, 40.58, 40.37, 39.32, 30.46; analysis for C_15_H_16_N_2_O_3_; calcd%: C, 66.23; H, 5.95; N, 10.25; found%: C, 66.52; H, 5.98; N, 10.29.; ESI-HRMS (*m*/*z*): anal. calcd. for C_15_H_16_N_2_O_3_ [M + H]^+^ 272.79; found: 272.47 (M + 1).

#### 3.4.11. 3-(2-(Morpholin-2-yl)acetyl)-2*H*-chromen-2-one (7b)

Condensation of an equimolar concentration of methanolic solution of 3-acetyl-coumarin and morpholine with formaldehyde in presence of dilute HCl obtained yellowish white powder; yield: 78%; white powder; UV-visible (*λ*_max_, CH_3_OH): 292 nm; IR (ATR, *γ*, cm^−1^): 3344 (Ar CH str.), 2853 (CH_2_ str.), 1732 (C=O str.), 1676 (C=N str.), 1606 (C=C str.), 1556 (CH Ar), 1264 (C–O str.); ^1^H NMR (DMSO-*d*_6_*δ*ppm, 400 MHz); 8.65 (s, 1H, coumarinyl H-4), 7.94 (s, 1H, coumarinyl H-5), 7.42 (s, 1H, coumarinyl H-6), 7.75 (s, 1H, coumarinyl H-7), 7.47 (s, 1H, coumarinyl H-8), 2.59 (m, 3H, CH̲_3_), 2.51 (s, 2H, CH̲_2_), 1.22 (s, 2H, CH̲_2_); ^13^C NMR (400 MHz, DMSO-*D*_6_); 195.59, 158.89, 155.07, 147.49, 134.94, 131.23, 125.41, 124.92, 118.62, 116.57, 40.61, 40.40, 39.57, 39.36, 30.46; analysis for C_15_H_15_NO_4_; calcd%: C, 65.93; H, 5.55; N, 5.25; found%: C, 66.98; H, 5.64; N, 5.68.; ESI-HRMS (*m*/*z*): anal. calcd. for C_15_H_15_NO_4_ [M +H]^+^ 273.79; found: 273.47 (M + 1).

#### 3.4.12. 6-Bromo-3-(2-(morpholin-2-yl)acetyl)-2*H*-chromen-2-one (7c)

Condensation of an equimolar concentration of methanolic solution of 6-bromo-3-acetyl-coumarin and morpholine with formaldehyde in presence of dilute HCl obtained yellowish white powder; yield: 78%; white powder; UV-visible (*λ*_max_, CH_3_OH): 284 nm; IR (ATR, *γ*, cm^−1^): 3338 (Ar CH str.), 2924 (CH_2_ str.), 1733 (C=O str.), 1675 (C=N str.), 1607 (C=C str.), 1549 (CH Ar), 1278 (C–O str); ^1^H NMR (DMSO-*d*_6_*δ*ppm, 400 MHz); 8.60 (s, 1H, coumarinyl H-4), 7.90 (s, 1H, coumarinyl H-5), 7.59 (s, 1H, coumarinyl H-7), 7.44 (s, 1H, coumarinyl H-8), 3.36 (s, 1H, CH̲_3_), 2.58 (m, 2H, CH̲_2_), 2.50 (m, 3H, CH̲_3_), 1.24 (s, 1H, CH̲_2_); ^13^C NMR (400 MHz, DMSO-*D*_6_); 195.55, 158.88, 156.06, 146.08, 137.05, 133.00, 120.54, 118.89, 116.82, 40.61, 40.40, 40.20, 39.36, 30.45; analysis for C_15_H_14_BrNO_4_; calcd%: C, 51.54; H, 4.17; N, 3.82; found%: C, 51.94; H, 4.74; N, 3.97.; ESI-HRMS (*m*/*z*): anal. calcd. for C_15_H_14_BrNO_4_ [M + H]^+^ 350.51; found: 351.47 (M + 1).

#### 3.4.13. 6-Bromo-3-(2-(pyrrolidin-2-yl)acetyl)-2*H*-chromen-2-one (7d)

Condensation of an equimolar concentration of methanolic solution of 6-bromo-3-acetyl-coumarin and pyrrolidine with formaldehyde in presence of dilute HCl obtained yellowish whitepowder; yield: 78%; white powder; UV-visible (*λ*_max_, CH_3_OH): 284 nm; IR (ATR, *γ*, cm^−1^): 3356 (Ar CH str.), 2843 (CH_2_ str.), 1716 (C=O str.), 1669 (C=N str.), 1624 (C=C str.), 1201 (C–N str.); ^1^H NMR (DMSO-*d*_6_ δppm, 400 MHz); 8.54 (s, 1H, coumarinyl H-4), 8.63 (s, 1H, coumarinyl H-5), 7.89 (s, 1H, coumarinyl H-7), 7.44 (s, 1H, coumarinyl H-8), 3.61 (s, 1H, CH̲_2_), 2.55 (s, 1H, CH̲_3_), 1.23 (s, 2H, CH̲_2_); ^13^C NMR (400 MHz, DMSO-*D*_6_); 158.45, 154.09, 146.08, 137.05, 132.99, 125.92, 120.53, 118.88, 116.81, 40.61, 40.40, 40.19, 39.96, 39.77, 30.44; analysis for C_15_H_14_BrNO_3_; calcd%: C, 53.54; H, 4.19; N, 4.21; found%: C, 53.94; H, 4.74; N, 4.97.; ESI-HRMS (*m*/*z*): anal. calcd. for C_15_H_14_BrNO_3_[M + H]^+^ 335.51; found: 336.47 (M + 1).

#### 3.4.14. 3-(2-(Pyrrolidin-2-yl)acetyl)-2*H*-chromen-2-one (7e)

Condensation of an equimolar concentration of methanolic solution of 3-acetyl-coumarin and pyrrolidine with formaldehyde in presence of dilute HCl obtained yellowish white powder; yield: 78%; white powder; UV-visible (*λ*_max_, CH_3_OH): 306 nm; IR (ATR, *γ*, cm^−1^): 3322 (Ar CH str.), 2969 (CH_2_ str.), 1712 (C=O str.), 1682 (C=N str.), 1606 (C=C str.), 1227 (C–N str.); ^1^H NMR (DMSO-*d*_6_ δppm, 400 MHz); 7.59 (s, 1H, coumarinyl H-5), 7.45 (s, 1H, coumarinyl H-7), 7.38 (s, 1H, coumarinyl H-8), 7.36 (s, 1H, coumarinyl H-6), 7.21 (s, 1H, coumarinyl H-4), 3.75 (s, 1H, CH̲_2_), 2.51 (s, 1H, CH̲_3_), 1.28 (s, 1H, CH̲_2_), 1.24 (s, 1H, CH̲_3_); ^13^C NMR (400 MHz, DMSO-*D*_6_); 160.30, 138.91, 130.80, 120.42, 111.31, 116.81, 40.88, 40.59, 40.39, 40.18, 39.97, 39.76, 39.55, 39.34, 22.87; analysis for C_15_H_15_NO_3_; calcd%: C, 70.24; H, 5.67; N, 5.22; found%: C, 70.98; H, 5.94; N, 5.69.; ESI-HRMS (*m*/*z*): anal. calcd. for C_15_H_15_NO_3_ [M + H]^+^ 258.51; found: 259.47 (M + 1).

#### 3.4.15. 3-(Diethylglycyl)-2*H*-chromen-2-one (7f)

Condensation of an equimolar concentration of methanolic solution of 3-acetyl-coumarin and *N*,*N*-diethylamine with formaldehyde in presence of dilute HCl obtained yellowish white powder yield: 78%; white powder; UV-visible (*λ*_max_, CH_3_OH): 310 nm; IR (ATR, *γ*, cm^−1^): 3336 (Ar CH str.), 2855 (CH_2_ str.), 1738 (C=O str.), 1674 (C=N str.), 1612 (C=C str.), 1556 (CH Ar), 1264 (C–O str.); ^1^H NMR (DMSO-*d*_6_ δppm, 400 MHz); 8.64 (s, 1H, coumarinyl H-4), 7.95 (s, 1H, coumarinyl H-5), 7.74 (s, 1H, coumarinyl H-6), 7.93 (s, 1H, coumarinyl H-7), 7.76 (s, 1H, coumarinyl H-8), 3.38 (s, 6H, CH̲_3_), 2.58 (m, 4H, CH̲_2_), 2.55 (s, 3H, CH̲_3_); ^13^C NMR (400 MHz, DMSO-*D*_6_); 195.58, 158.89, 155.06, 147.50, 134.94, 131.22, 125.40, 124.89, 118.61, 116.56, 40.87, 40.59, 40.38, 39.34, 30.; analysis for C_15_H_17_NO_3_; calcd%: C, 69.54; H, 6.19; N, 5.28; found%: C, 69.88; H, 6.91; N, 5.99.; ESI-HRMS (*m*/*z*): anal. calcd. for C_15_H_17_NO_3_ [M + H]^+^ 259.51; found: 259.97 (M + 1).

#### 3.4.16. 6-Bromo -3-(2-(piperidin-2-yl)acetyl)-2*H*-chromen-2-one (7g)

Condensation of an equimolar concentration of methanolic solution of 6-bromo-3-acetyl-coumarin and piperidine with formaldehyde in presence of dilute HCl obtained yellowish white powder; yield: 78%; white powder; UV-visible (*λ*_max_, CH_3_OH): 309 nm; IR (ATR, *γ*, cm^−1^): 3314 (Ar CH str.), 2932 (CH_2_ str.), 1714 (C=O str.), 1679 (C=N str.), 1605 (C=C str.), 1224 (C–N str.); ^1^H NMR (DMSO-*d*_6_*δ*ppm, 400 MHz); 8.60 (s, 1H, coumarinyl H-4), 8.21 (s, 1H, coumarinyl H-5), 7.66 (s, 1H, coumarinyl H-7), 7.24 (s, 1H, coumarinyl H-8), 3.37 (s, 2H, CH̲_2_), 3.15 (m, 2H, CH̲_3_), 3.11 (s, 2H, CH̲_3_), 2.58 (m, 3H, CH̲_3_), 2.19 (m, 2H, CH̲_3_), 1.86 (s, 2H, CH̲_2_); ^13^C NMR (400 MHz, DMSO-*D*_6_); 195.44, 190.16, 160.34, 158.46, 154.09, 146.10, 138.93, 137.05, 133.00, 130.93, 125.89, 120.53, 120.39, 118.87, 116.82, 40.60, 30.46; analysis for C_16_H_16_BrNO_3_; calcd%: C, 54.15; H, 4.87; N, 4.22; found%: C, 55.01; H, 5.12; N, 5.12; ESI-HRMS (*m*/*z*): anal. calcd. for C_16_H_16_BrNO_3_ [M + H]^+^ 350.21; found: 351.23 (M + 1).

### 3.5 Antimicrobial activity

The majority of the derivatives displayed varying degrees of antibacterial efficacy against Gram-positive bacteria, including *Klebsiella pneumoniae*, and *Staphylococcus aureus*, with *Escherichia coli* used as a standard. However, they also exhibited a range of responses from moderate to resistant effects specifically against *K. pneumoniae* and *S. aureus*. Notably, compound 5d, featuring a 4-nitrophenyl moiety within its coumarin ring structure, exhibited heightened antibacterial activity against *K. pneumoniae*, with an MIC value of 16 μg mL^−1^. Conversely, it displayed a milder antibacterial effect against *S. aureus*, with an MIC value of 8 μg mL^−1^. Similarly, compound 5f showed MIC values of 16 μg mL^−1^ against *K. pneumoniae* and 12 μg mL^−1^ against *S. aureus*. The results from the assessment of compounds 5d and 5f against both strains underscored *Staphylococcus aureus* as the most affected strain, demonstrating significant antibacterial activity. Moreover, the standard ciprofloxacin, when subjected to the same testing conditions against these two strains, exhibited MIC values of 16 μg mL^−1^ and 8 μg mL^−1^, respectively.

### 3.6 Antibiofilm activity

**Fig. 1:**
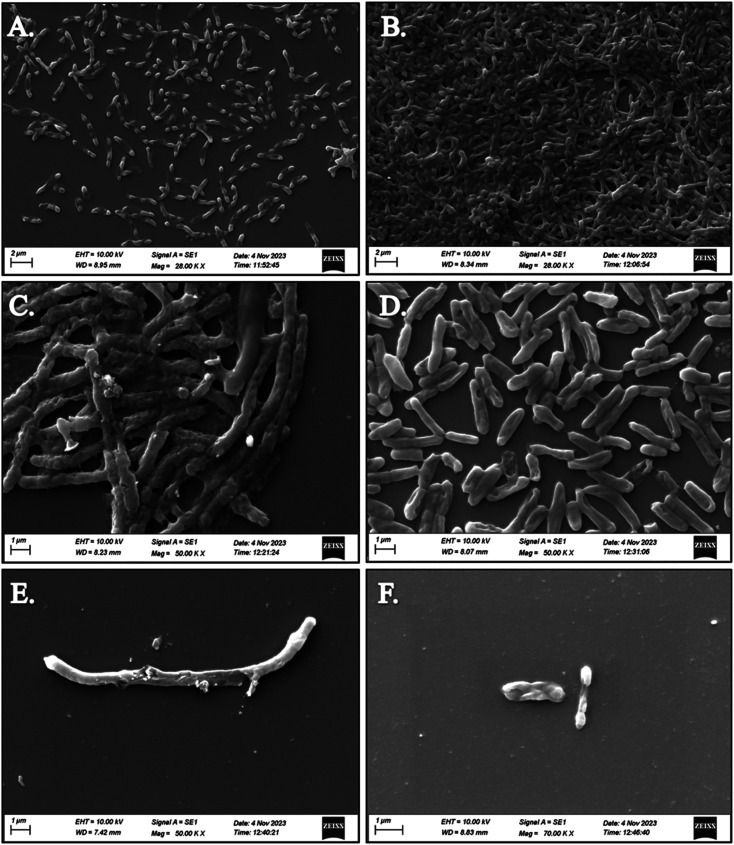
SEM image of *K. pneumoniae* and *S. aureus* with Compound 5d & 5f (A) K. *Pneumoniae* standard (B) compound 5d-treated with *K. pneumoniae*, (C) Compound 5f-treated with *K. pneumoniae* (D) *S. aureus* standard (E) compound 5d-treated with *S. aureus*, (F) compound 5f-treated with *S. aureus*.

SEM analysis revealed significant disruptions in the biofilm architecture of both *Klebsiella pneumoniae* and *Staphylococcus aureus* upon treatment with compounds 5d and 5f. Compared to untreated biofilms, those treated with the compounds exhibited reduced biomass and altered surface morphology, indicative of biofilm inhibition. Moreover, the disruption of biofilm integrity was more pronounced in the presence of *S. aureus* treated with compounds 5d and 5f, suggesting their enhanced efficacy against both bacterial strains. The observed disruption of biofilm structures by compounds 5d and 5f highlights their potential as effective agents for combating biofilm-associated infections caused by *K. pneumoniae* and *S. aureus*. The SEM images of both strains treated with compounds 5d and 5f depict the superior antibiofilm activity against *S. aureus* compared to *K. pneumoniae*, as shown in Fig. 1. By targeting biofilm formation, these compounds offer a promising strategy to overcome antibiotic resistance and improve treatment outcomes in bacterial infections.

### 3.7 Molecular docking study

**Fig. 2:**
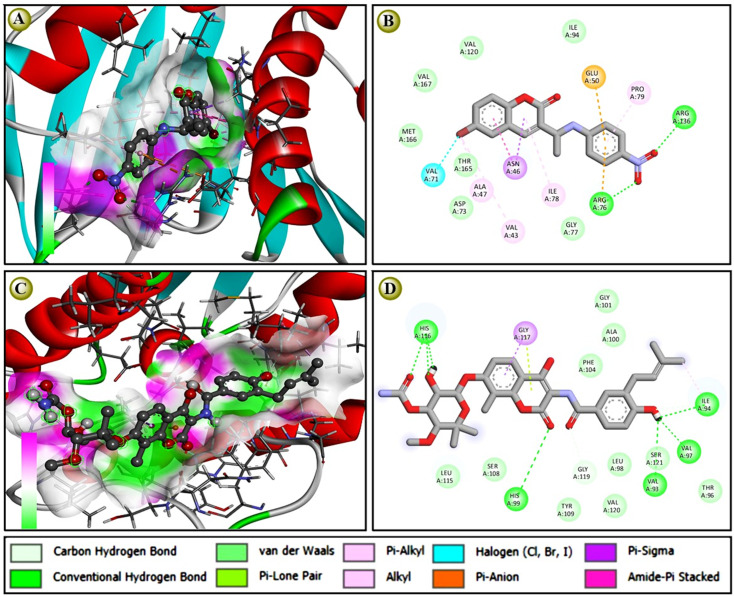
The protein-ligand interaction of most potential candidate with standard antibiotic during molecular docking study: (A), 3D interaction study of EC_GyrB with 5d; (B), 2D interaction study of EC_GyrB with 5d; (C), 3D interaction study of EC_GyrB with novobiocin; and (D) 2D interaction study of EC_GyrB with novobiocin. Images were generated and presented using BIOVIA-Discovery Studio Visualizer 2021 and ChemDraw 2021 software, respectively.

The molecular docking score of designed coumarin derivatives along with standard Novobiocin against Four selected targets is depicted in Table 3. Based on the molecular docking, the most coumarin derivatives showed their binding energy within the range of −6 to −8 kcal mol^−1^. Notable, the compound 5d (−8.5), the next highest 5f, 5g, 5m, and 5o; 5c (−8.2); 5a, 5e, and 5i (−8.1) are some of the most potential candidates against *E. coli* gyrase B with a docking score of ≤ −8.0 kcal mol^−1^. Similarly, 5d (−7.8), 5f (−7.6), and 5c (−7.0) are potential candidates with docking scores ≤ −7.0 kcal mol^−1^ against *S. aureus* gyrase B (Table 3), 5d (−8.5); 5f (−8.3); 7c (−8.1); and 5j and 5k (−8.0) have potential against *A. baumannii* gyrase B. Similarly, against *S. aureus* gyrase B and biofilm-associated target enzyme FabG (*K. pneumoniae*), both 5d (−7.8 and −8.3) and 5f (−7.9 and −8.5) are also on the lead candidates, where the standard novobiocin showed a docking score within the score −8.1 to −8.7 against all target enzymes. Although the compounds 5d and 5f had a potential docking score against all four target enzymes that was nearly equal to the standard antibioticl anticipated *in vitro* antibacterial reports (Table 4). A protein-ligand interaction study revealed that novobiocin showed seven hydrogen bond interactions with a few van der Waals pi-alkyl and pi–loan pair interactions against DNA gyrase B of *E. coli*, while 5d showed two hydrogen bonds, five pi-alkyl, pi-sigma, and amide pi-stacked, pi-anion/cation, along with a halogen bond interaction against the same target Fig. 2. The protein-ligand interaction also suggested that due to the higher number of h-bonds, novobiocin showed a comparatively higher binding affinity than 5d; however, the docking score of the proposed derivative, which was near equal to the standard, suggested that the proposed derivatives have such multimodal antibacterial target-specific activity that they could be used as potential antibacterial.

### 3.8 Molecular dynamics (MD) simulation

**Fig. 3:**
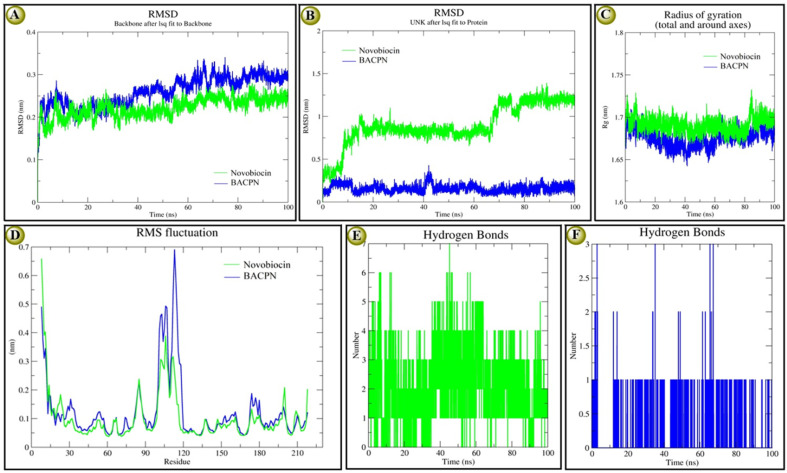
Conformational stability of most EC_GyrB-5d and EC_GyrB-novobiocin docking complexes at 100 ns MD simulation in individual colour plots: (A), overlaid RMDS-plots of EC_GyrB-5d (blue colour) and EC_GyrB-novobiocin (green colour) of backbone proteins; (B), overlaid RMDS-plots of ligand 5d and novobiocin in the docking complex; (C), overlaid Rg-plots of EC_GyrB-5d and EC_GyrB-novobiocin; (D), overlaid RMSF-plots of EC_GyrB-5d and EC_GyrB-novobiocin along with molecular stability based on H-bond interactions (E), represents H-bond interactions of novobiocin; and (F), represents H-bond interactions of 5d with EC_GyrB.

The molecular stability reports based on the investigated RMSD, RMSF, Rg, and H-bond plots of both EC_GyrB-5d and EC_GyrB-novobiocin docking complexes at 100 ns are recorded in Fig. 3. According to backbone protein RMSD plots, both complexes showed similar types of deviation, especially novobiocin, which deviated comparatively at a lower range (0.10 to 0.15 nm), while 5d deviated at 0.10 to > 0.30 nm. Both novobiocin (1 to 37 ns) and 5d (1 to 18 ns) showed a higher deviation at the initial stage. However, later, novobiocin showed comparatively stable 5d, and at the end of the 100 ns, both showed similar stability Fig. 3A. On the other hand, the overlaid ligand-RMSD plots indicated that 5d was more stable than novobiocin Fig. 3B. After 20 ns and except for 40 to 42 ns, 5d was highly stable, while before 10 ns and after 58 to 60 ns, novobiocin shifted to a different level and maintained a highly stable range from 90 to 100 ns similar to 5dFig. 3B. The Rg-plots of both docking complexes indicated that even though both showed similar trends, novobiocin showed higher stability than 5d within 100 ns Fig. 3C. The overlaid RMSF plot also indicated that 5d had a comparatively higher deviation than novobiocin in a similar trend. Briefly, the interactive residues within 92 and 120, both ligands, show higher instability and deviation Fig. 3D. In terms of stability in terms of the number of H-bond interactions, we found that at the initial stage, novobiocin showed four to six H-bonds, and around 44 ns formed seven H-bonds Fig. 3E. Similarly, 5d showed only one H-bond at the initial stage, showed the highest three H-bonds at three different time points, and overall maintained one or two H-bonds. At the end of the 100 ns, 5d showed one H-bond and novobiocin showed two H-bonds, indicating novobiocin was comparatively more stable against *E. coli* gyrase B. The MD simulation supported the presented docking score and interaction results, and overall, it indicated 5d was nearly as stable as novobiocin.

### 3.9 Physicochemical properties

The candidates accommodate Schiff's base and Mannich base coumarin congeners (5a–5i) and (7a–7g) were noted to be in great concurrence with every one of the boundaries of Lipinski's Standard, but the mixtures containing tetra-subbed have viewed as in their higher cutoff for sub-atomic weight and *c* log *P* esteem. The compounds disregarding the standard anyway have been found to have great concurrence with the harmfulness forecast, thusly the hydrophilicity and hydrogen acceptor boundaries of a necessary competitor could be changed by adding proper adjuvant or vehicle at the hour of organization. The RO5 boundaries have been determined by the web-based accessible databases *viz.*, Molinspiration (http://www.molinspiration.com/) and Molsoft (http://molsoft.com/mprop/) and reevaluated through the ChemDraw programming, which was very much delineated in Table 2. The hypothetical computation of absorption, distribution, metabolism, and elimination, end of blended Schiff's base and Mannich base coumarin congeners was performed utilizing pre-ADMET https://preadmet.webservice.bmdrc.org/adme/ including blood–brain barrier (BBB), Caco-2 permeability (the Caco-2 cells for cell penetrability into human gastrointestinal cell boundary), human gastrointestinal retention (produces permeability apportion of bioavailability and absorption) and skin permeability, likewise, the deadly portion LD_50_ for the potent Schiff's base and Mannich base coumarin congeners have been anticipated alongside the harmfulness class going from 150 to 1500, not entirely set in stone by ProTox (http://tox.charite.de/tox/) recorded in Table 1. Every one of the candidates showed a protected profile while the mixtures bearing nitro group joined to the coumarinyl showed the most secure profile, which could be a characterized approach for the compound 5d which showed early antimicrobial and antibiofilm against different bacterial strains to be a lead candidate for impending antimicrobial disclosure journey.

**Table 2:** Lipinski rule of five of the newly synthesized coumarin congeners 5a–5g & 7a–7i

Compound	R	MW	HA	HB	*c* log *P*	tpsA
5a	4-SO_3_H	343.35	18	1	4.26	105.32
5b	4-OCH_3_	372.21	17	0	4.7	51.8
5c	2-NO_2_	387.19	15	0	5.13	88.39
5d	4-NO_2_	387.18	14	0	5.13	88.39
5e	4-COOH	307.34	4	1	3.63	79.87
5f	3-NO_2_	387.18	14	0	5.13	88.39
5g	2-NO_2_	308.08	5	0	4.39	88.39
5h	Thiazol-2yl	270.31	13	0	3.39	83.7
5i	Pyridin-2-yl	264.28	3	0	3.33	55.46
7a	Piperazinyl	271.31	3	1	2.84	59.31
7b	Morpholin-2-yl	273.28	19	1	1.68	68.54
7c	Morpholin-4-yl	352.18	18	1	2.45	68.54
7d	Pyrrolidinyl	336.18	17	1	2.31	59.31
7e	Pyrrolidinyl	257.28	18	1	2.45	59.31
7f	*N*,*N*-Diethylamino	259.31	20	0	2.32	50.74
7g	Piperidinyl	350.21	19	1	3.6	59.31

**Table 3:** Molecular docking scores (kCal/mol) of newly designed coumarin congeners5a–5g & 7a–7i^a^

Sl. no.	Proposed coumarin derivatives	EC_GyrB (PDB ID: 7P2M)	SA_GyrB (PDB ID: 5D7R)	AB_GyrB (PDB ID: 7PQI)	KP_FabG (PDB ID: 6T77)
1	5a	−8.1	−6.8	−7.7	−7.5
2	5b	−7.5	−6.5	−7.3	−7.9
3	5c	−8.2	−7.0	−8.0	−8.1
4	5d	**−8.5**	**−7.8**	**−8.3**	**−8.5**
5	5e	−8.1	−6.5	−7.9	−7.0
6	5f	**−8.4**	**−7.6**	**−8.3**	**−8.3**
7	5g	−8.4	−6.7	−7.6	−7.6
8	5h	−7.5	−5.7	−6.9	−7.9
9	5i	−8.1	−6.0	−8.1	−7.7
10	5j	−7.4	−6.6	−7.5	−8.0
11	5k	−7.9	−6.7	−7.6	−8.0
12	5l	−7.9	−7.0	−7.8	−7.2
13	5m	−8.2	−6.5	−8.0	−7.9
14	5n	−7.3	−6.1	−7.8	−6.6
15	5o	−8.4	−6.8	−8.5	−7.6
16	7a	−8.1	−6.1	−7.9	−7.4
17	7b	−7.5	−5.7	−7.2	−7.5
18	7c	−7.0	−6.1	−7.8	−8.1
19	7d	−7.6	−6.2	−7.3	−7.3
20	7e	−8.1	−6.5	−7.7	−7.5
21	7f	−6.5	−5.0	−6.5	−7.0
22	7g	−7.4	−5.9	−7.2	−8.8
23	7h	−7.3	−5.8	−7.2	−6.5
24	7i	−7.8	−5.8	−7.3	−7.3
25	7j	−6.9	−5.6	−6.7	−7.0
26	7k	−6.8	−5.4	−6.5	−6.9
27	7l	−7.2	−5.2	−6.9	−6.9
28	Novobiocin	−8.6	−8.1	−8.3	−8.7

a Compound 5d exhibits the highest binding score (−8.5) among all the compounds.

**Table 4:** Antimicrobial assessment of the coumarin derivatives 5a–5i, 7a–7g^a^

Compound	*K. pneumoniae*		*S. aureus*		*E. coli*	
	IZD (mm)	MIC (μg mL^−1^)	IZD (mm)	MIC (μg mL^−1^)	IZD (mm)	MIC (μg mL^−1^)
5a	12	128	11	128	10	64
5b	**20**	**16**	**19**	**32**	**18**	**4**
5c	12	64	10	128	14	128
5d	**23**	**4**	**23**	**4**	**24**	**1**
5e	12	128	—	—	12	—
5f	**25**	**4**	**22**	**8**	**22**	**1**
5g	10	—	12	—	12	—
5h	12	—	13	—	—	—
5i	12	128	15	128	12	128
7a	10	128	14	128	12	—
7b	11	—	13	128	18	—
7c	12	—	14	—	16	—
7d	13	—	12	—	12	—
7e	12	—	11	—	11	—
7f	12	—	12	64	10	—
7g	10	—	10	—	12	—
**Ciprofloxacin**	**28**	**4**	**32**	**2**	**28**	**1**
**Novobiocin**	**29**	**2**	**28**	**6**	**28**	**1**

a IZD: inhibition zone diameter; average IZD: 20–25 mm (good); >15 < 20 mm (moderate) and <15 mm (poor or inactive/resistant).

### 3.10 HOMO LUMO analysis

**Fig. 4:**
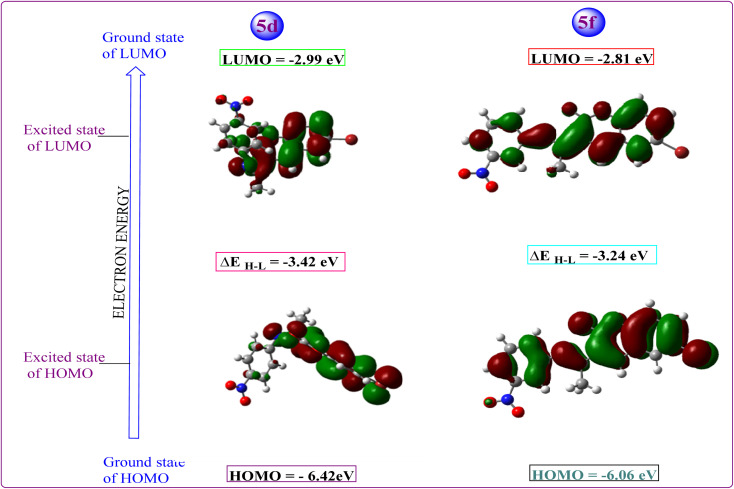
HOMO LUMO and energy gap of the potent candidates.

All hypothetical computations regarding stability were performed using Gaussian 09. Among all synthesized candidates, the potent candidates, 5d & 5f, were fully optimized without imposing any symmetry constraints employing the Becke three-parameter exchange functional combined with the Lee–Yang–Parr (B3LYP) correlation functional at the 6-311 + G(d,p) level. Electronic properties such as total energy (*E*), energies of the highest occupied molecular orbitals (E_HOMO_), and the lowest unoccupied molecular orbitals (E_LUMO_) were investigated. Notably, an inhibitory particle exhibits a high HOMO energy, as demonstrated by the calculated values. The HOMO and LUMO plots of the potent compounds are depicted in Fig. 4 where the HOMO acts as an electron donor while the LUMO serves as an electron acceptor. The energies of the HOMO and LUMO are fundamental quantum chemical descriptors, providing insights into the reactivity, shape, and binding behavior of molecules, as well as molecular substituents and fragments. Specifically, the calculated HOMO–LUMO energy gap for compound 5d is 3.42 eV and 5f 3.24 eV, respectively. It is observed that molecules with smaller energy gaps are more polarizable and tend to exhibit higher chemical reactivity, often categorized as “soft” molecules. Furthermore, this investigation explores the charge transfer property and charge distribution probability of the molecules, which are intricately linked to their pharmacological properties. The molecular electrostatic potential surface (MESP) serves as a tool to identify neutral, positive, and negative electrostatic potential domains through color grading, providing insights into the relative polarity of compounds. Specifically, red regions denote negative electrostatic potential corresponding to nucleophilic centers, while blue regions represent positive electrostatic surfaces, defining electrophilic centers within the system in 3D-dimensional charge distribution.

Furthermore, the molecular electrostatic potential (MESP) plot of the potent coumarin ligands 5d & 5f reveals a distinct region of high electronegativity surrounding the oxygen atom, indicative of nucleophilic centers. The isosurface computed in MESP directly correlates with the total electron density, with varying electrostatic potential values depicted by different colors on the surface, following the order: red < orange < yellow < green < blue.

**Fig. 5:**
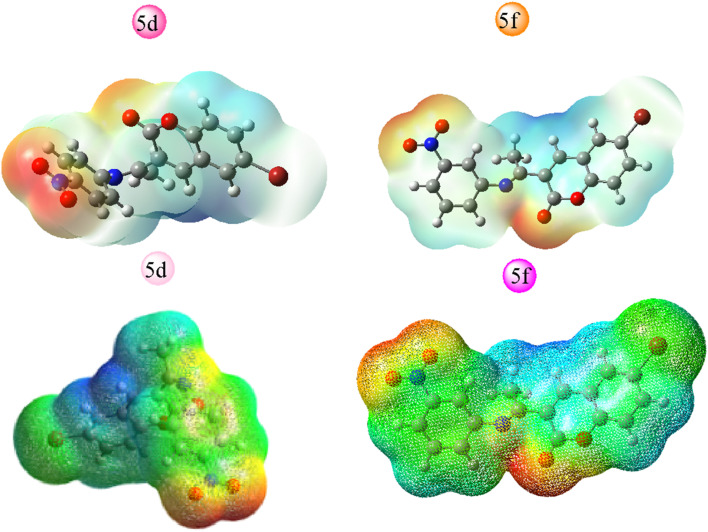
MESP plot of potent candidates.

This comprehensive analysis provides valuable insights into the molecular properties and potential interactions of the coumarin-derived compound, as well as the localization of different functional residues within its structure, and the figure depicted in Fig. 5. These findings suggest potential pharmacological behaviors of the compound.

## 4. Conclusion

In conclusion, the study focused on synthesized coumarin derivatives with inserted azomethine and amino methylated groups in an individual two distinct series of compounds exhibiting moderate to significant antibacterial properties. Through *in silico* investigation of designed compounds by molecular docking, molecular dynamics simulations, structural confirmation of synthesized compounds, and antibacterial assays, among them the compounds 5d and 5f emerged as potent candidates against *Staphylococcus aureus* and multidrug-resistant *Klebsiella pneumonia* and their efficacy was comparable to standard antibiotic Ciprofloxacin. They have a strong binding affinity to bacterial DNA gyrase and biofilm-producing bacterial strains. Moreover, the stability predicted toxicity and pharmacokinetic profiles of these compounds were evidenced favorable drug-ability. Collectively, these findings underscore the potential of compounds 5d and 5f as multifaceted antibacterial agents, paving the way for further pharmacological evaluations and eventually clinical applications in combating drug-resistant bacterial infections.

## Data availability

All the data are original and will be available on prior permission of the authors. The data supporting this article have been included as part of the ESI.†

## Conflicts of interest

The authors state that they do not have any identifiable conflicting financial interests or personal associations that might have seemed to impact the research presented in this paper.

## Supplementary Material

RA-014-D4RA05756B-s001
