# Supplementary material for: Multimodal antibacterial potency of newly designed and synthesized Schiff's/Mannich based coumarin derivatives: potential inhibitors of bacterial DNA gyrase and biofilm production
Source: RSC Adv. 2024 Oct 7;14(43):31633–47. doi: 10.1039/d4ra05756b (PMC11457008; doi:10.1039/d4ra05756b)
Supplement: RA-014-D4RA05756B-s001 [file RA-014-D4RA05756B-s001.pdf]

Supplementary Information (SI) for RSC Advances.
This journal is © The Royal Society of Chemistry 2024

Supplementary materials

Multimodal Antibacterial Potency of Novel Schiff / Mannich Based Coumarin Derivatives Targeting Bacterial DNA Gyrase and Biofilm Inhibition: An *In-Vitro* and *In-Silico* Investigations.

Kakarla Pakeeraiah[a], Pragyan Paramita Swain[b], Alaka Sahoo[a,c,d], Preetesh Kumar Panda[a], Monalisa Mahapatra[a], Suvadeep Mal[a], Rajesh Kumar Sahoo[b], Pratap Kumar Sahu*[e], Sudhir Kumar Paidesetty*[a]

[a] Medicinal Chemistry Research Laboratory, School of Pharmaceutical Sciences, Siksha 'O' Anusandhan Deemed to be University, Bhubaneswar 751003, Odisha, India.

[b] Centre for Biotechnology, School of Pharmaceutical Sciences, Siksha 'O' Anusandhan Deemed to be University, Bhubaneswar 751003, Odisha, India.

[c]Department of Skin & VD, Institute of Medical Sciences&SUM Hospital, Siksha 'O' Anusandhan Deemed to be University, Bhubaneswar 751003, Odisha, India.

[d] Research and Development Division, Salixiras Research Private Limited, Bhubaneswar, Odisha, India.

[e] Department of Pharmacology, School of Pharmaceutical Sciences, Siksha 'O' Anusandhan Deemed to be University, Bhubaneswar 751003, Odisha, India.

***Corresponding authors: psudhirkumar@soa.ac.in; pratapsahu@soa.ac.in**

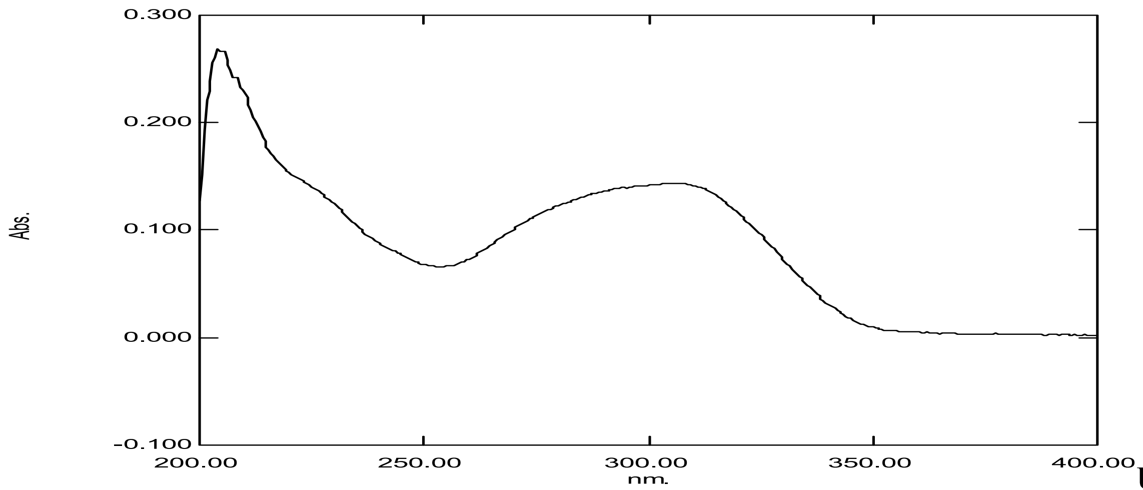

UV-
Fig. S1. Visible spectra of compound 5a

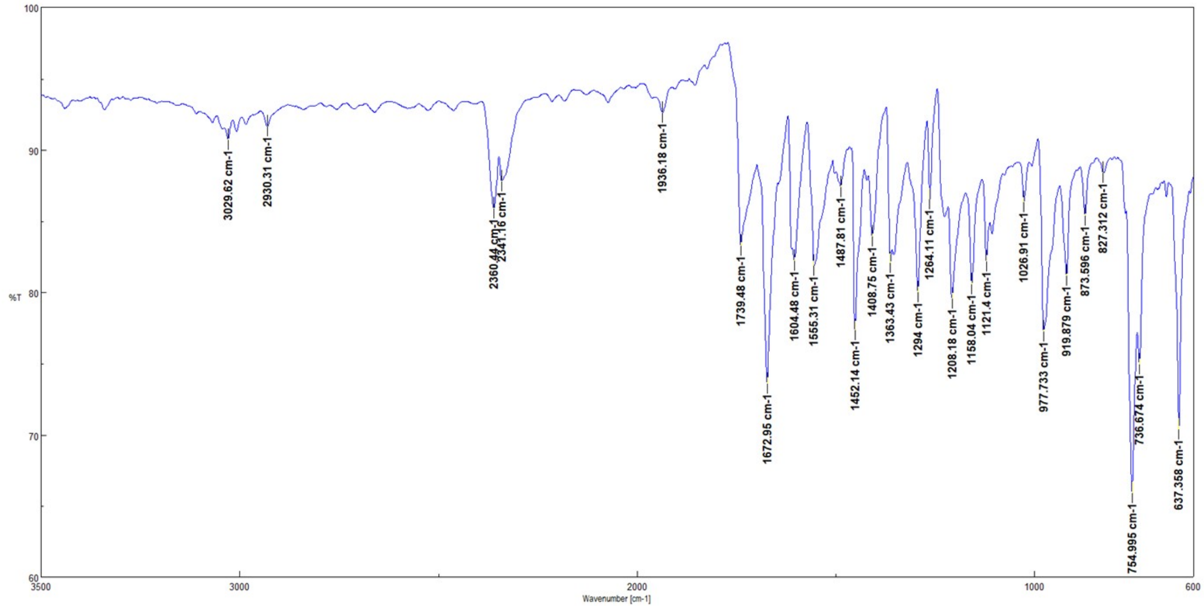

Fig. S2. FTIR Spectra of compound 5a

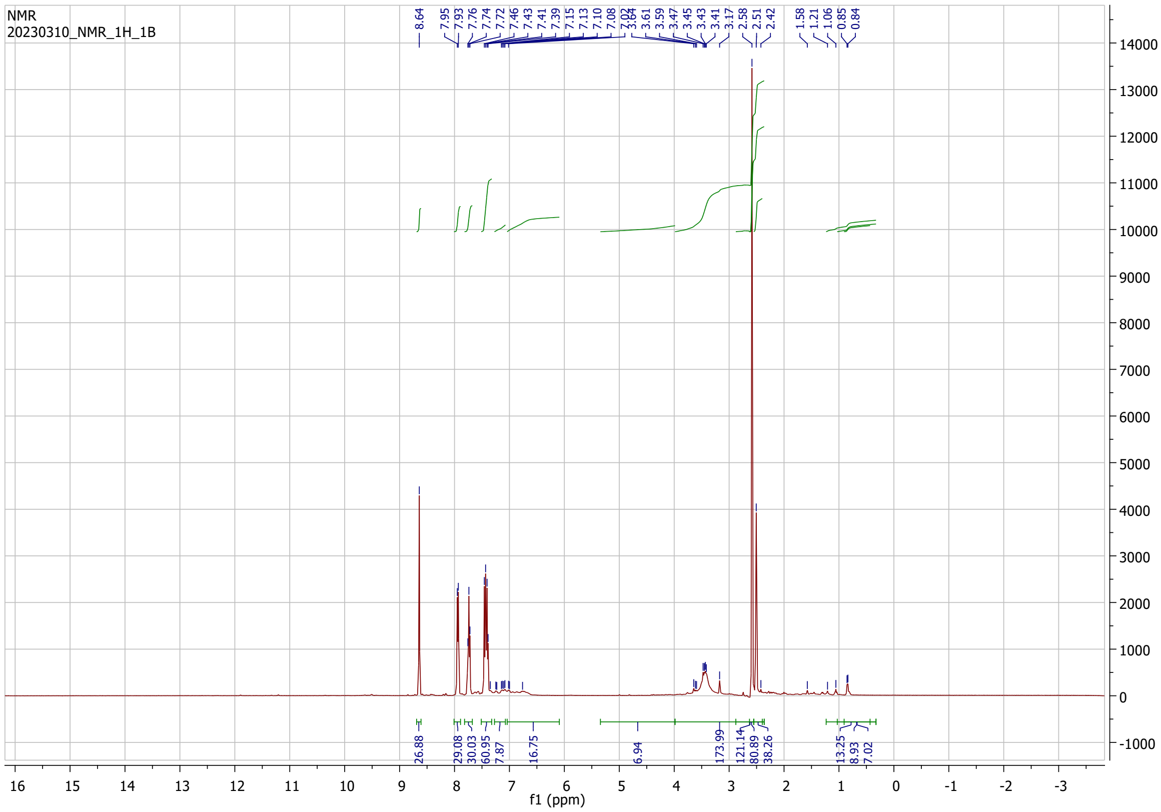

Fig. S3. ^{1}H NMR Spectra of compound 5a

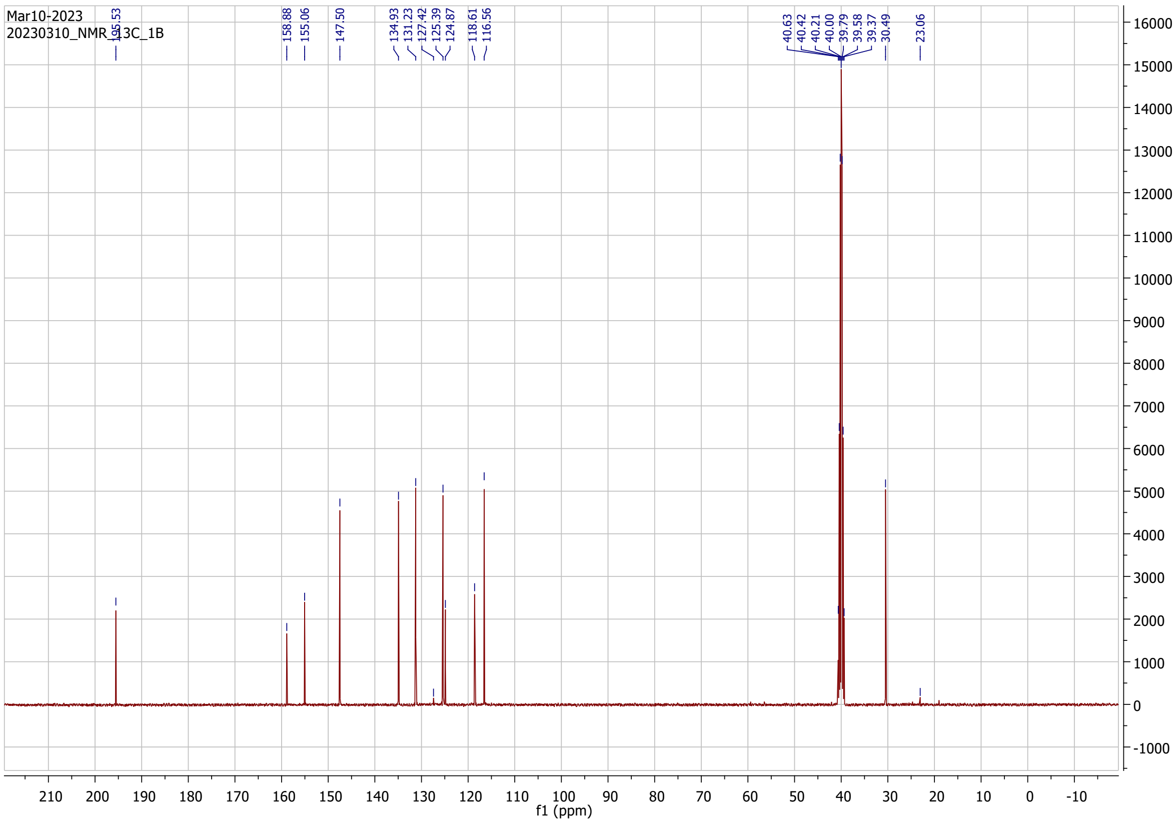

Fig. S4. ^{13}C NMR Spectra of compound 5a

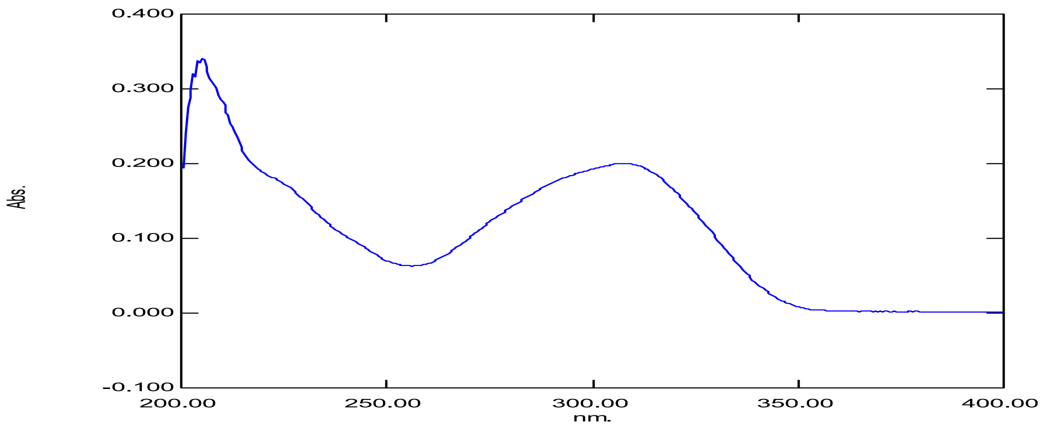

Fig. S5. UV-Visible spectra of compound 5b

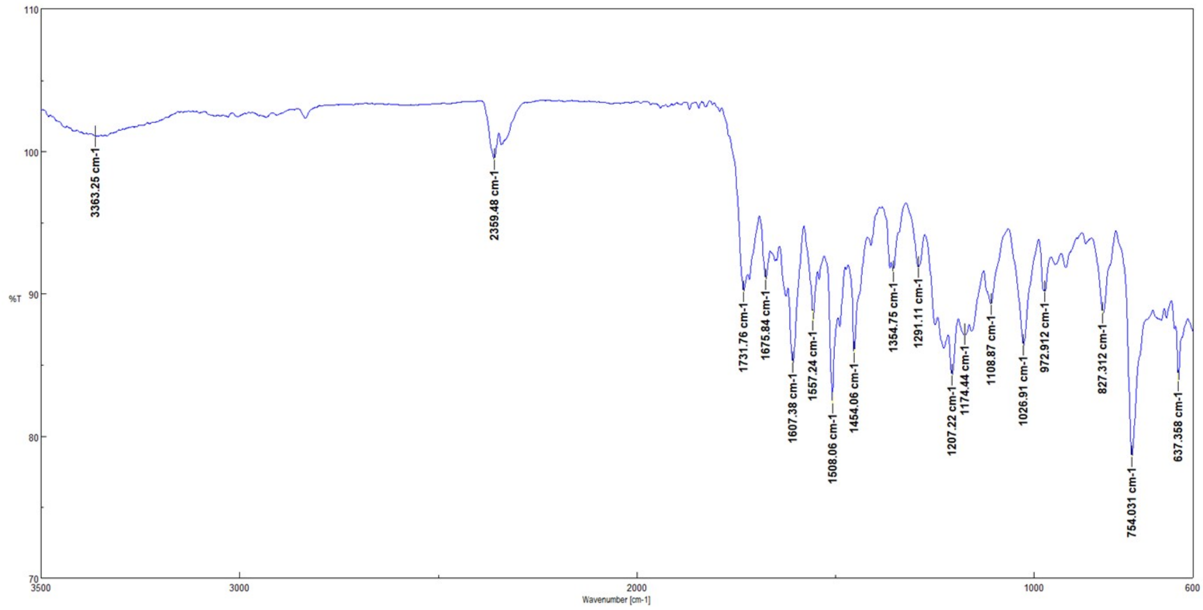

Fig. S6. FTIR Spectra of compound 5b

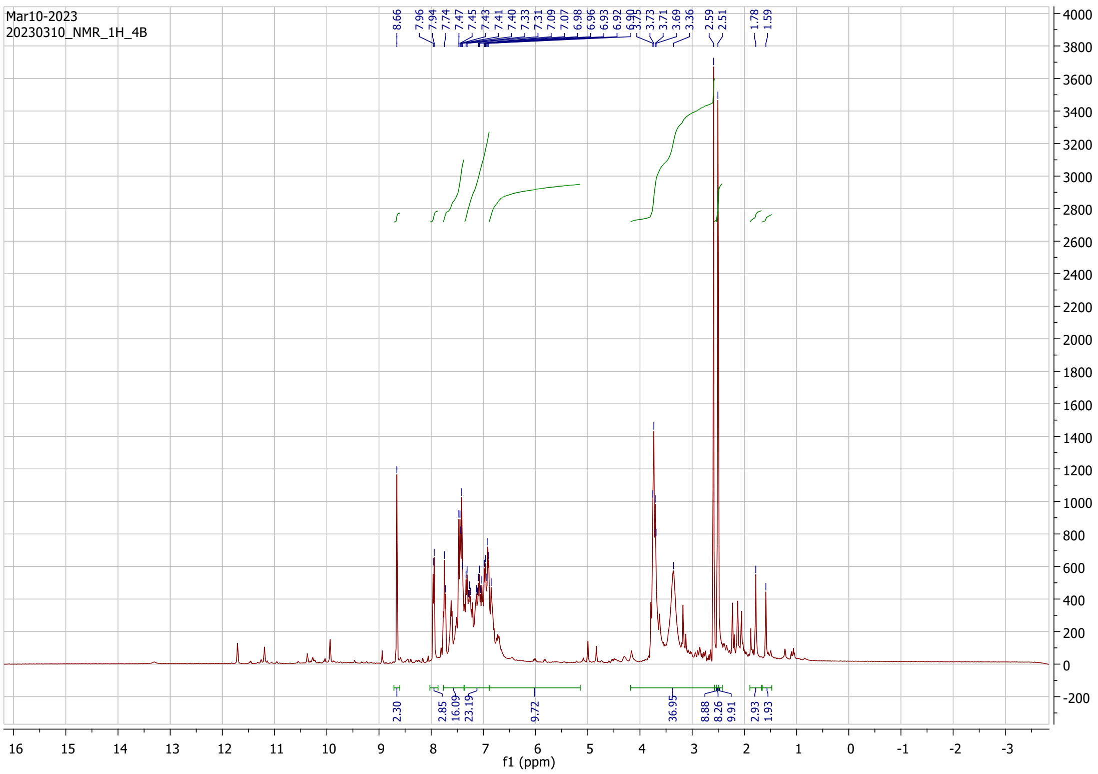

Fig. S7. ^{1}H NMR Spectra of compound 5b

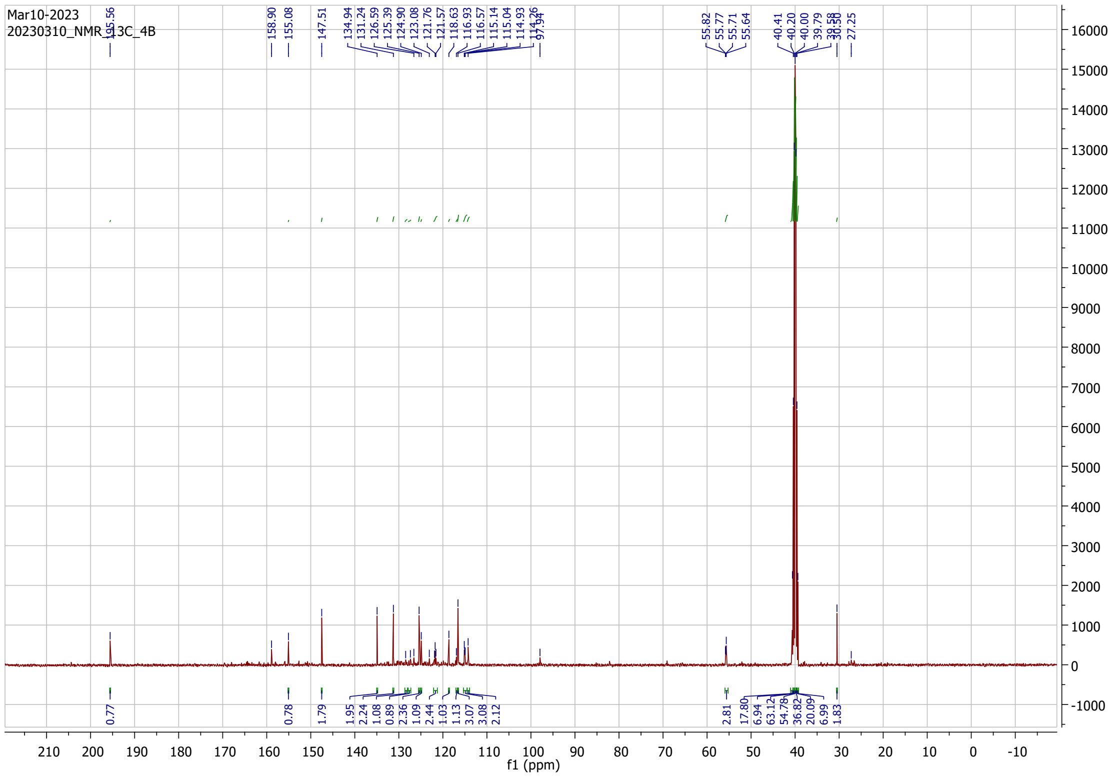

Fig. S8. ^{13}C NMR Spectra of compound 5b

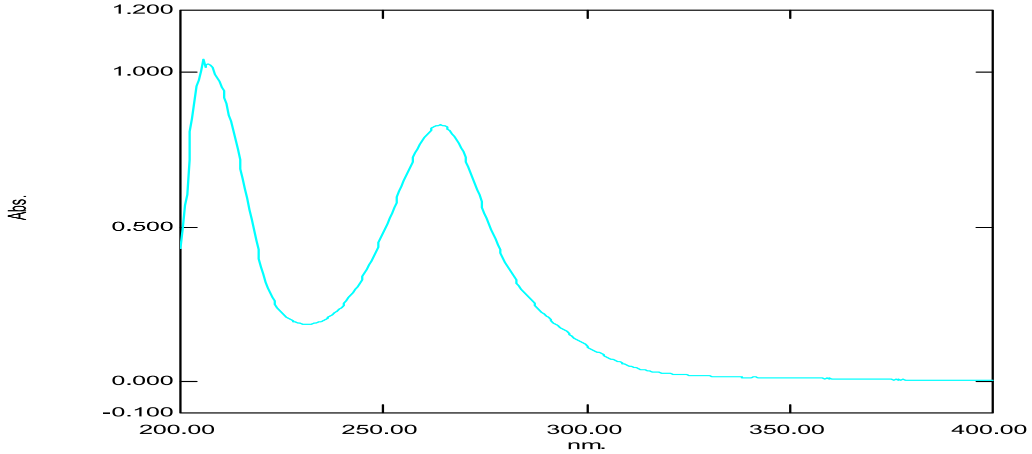

Fig. S9. UV-Visible spectra of compound 5c

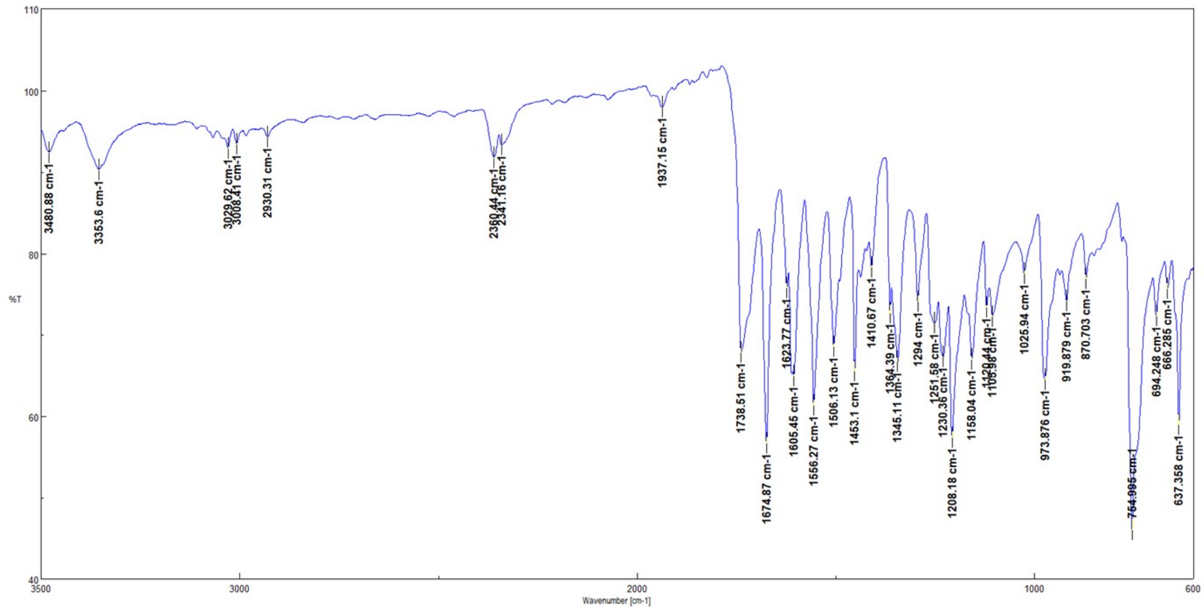

Fig. S10. FTIR Spectra of compound 5c

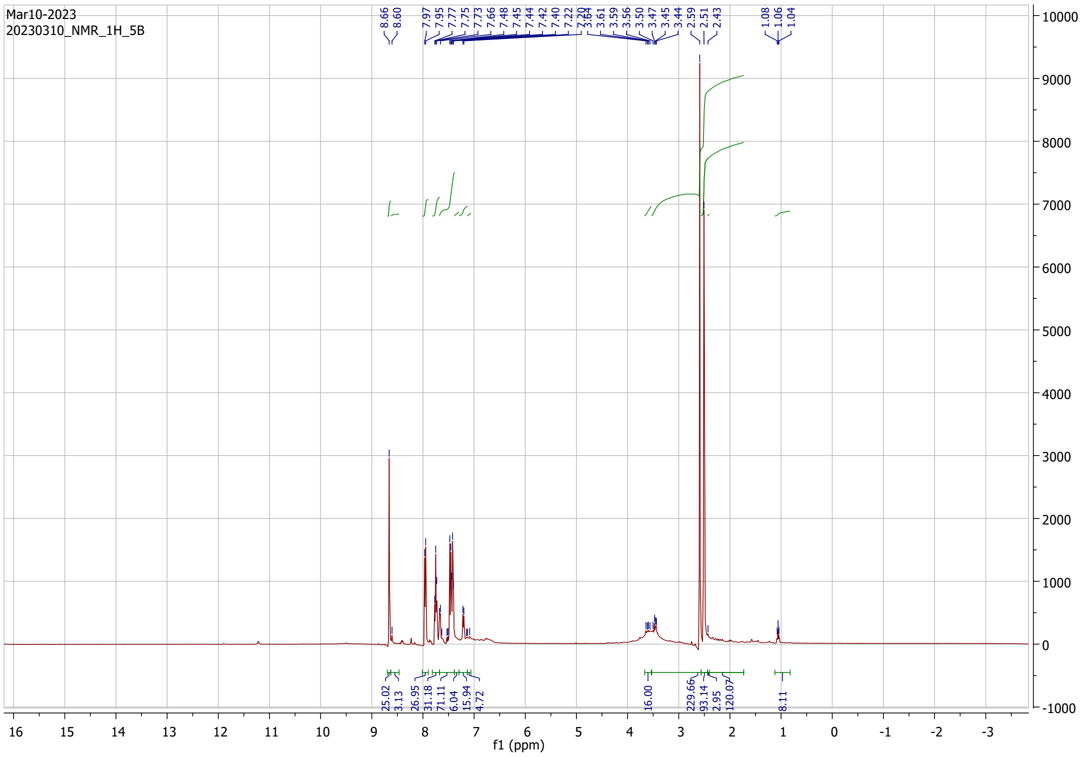

Fig. S11. ^{1}H NMR Spectra of compound 5c

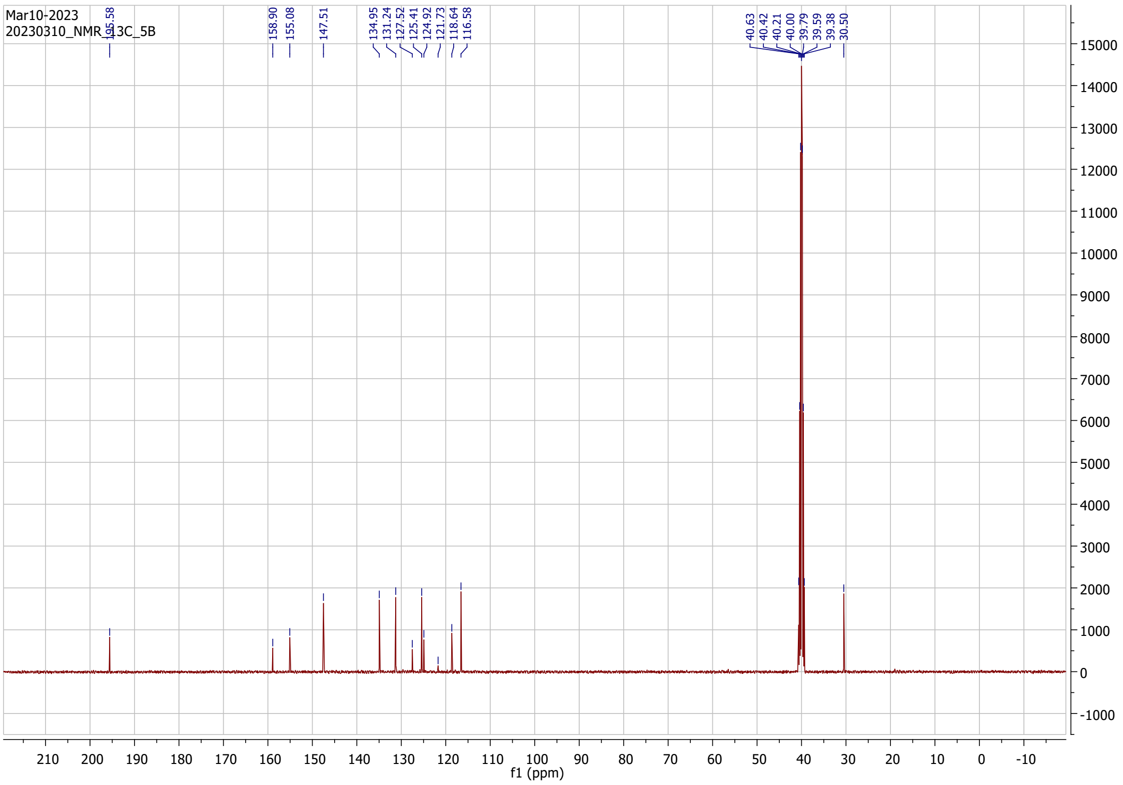

Fig. S12. ^{13}C NMR Spectra of compound 5c

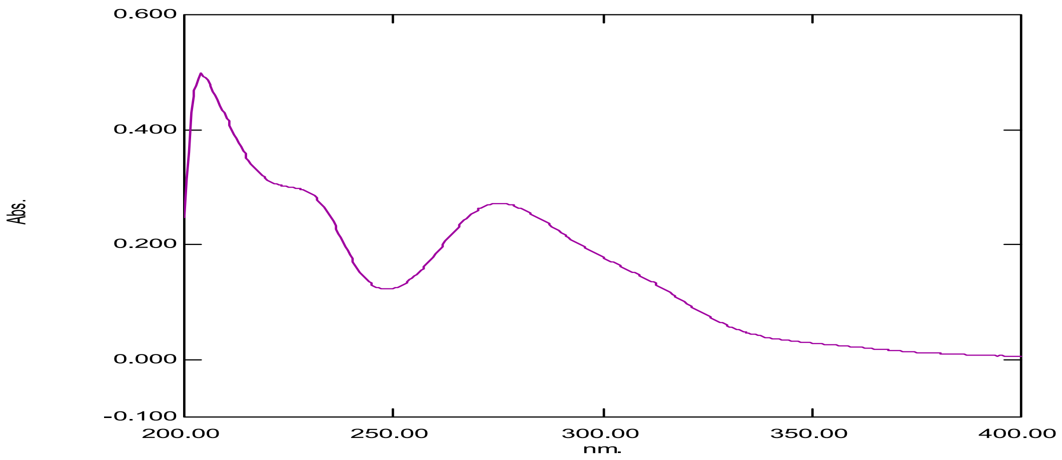

Fig. S13. UV-Visible spectra of compound 5d

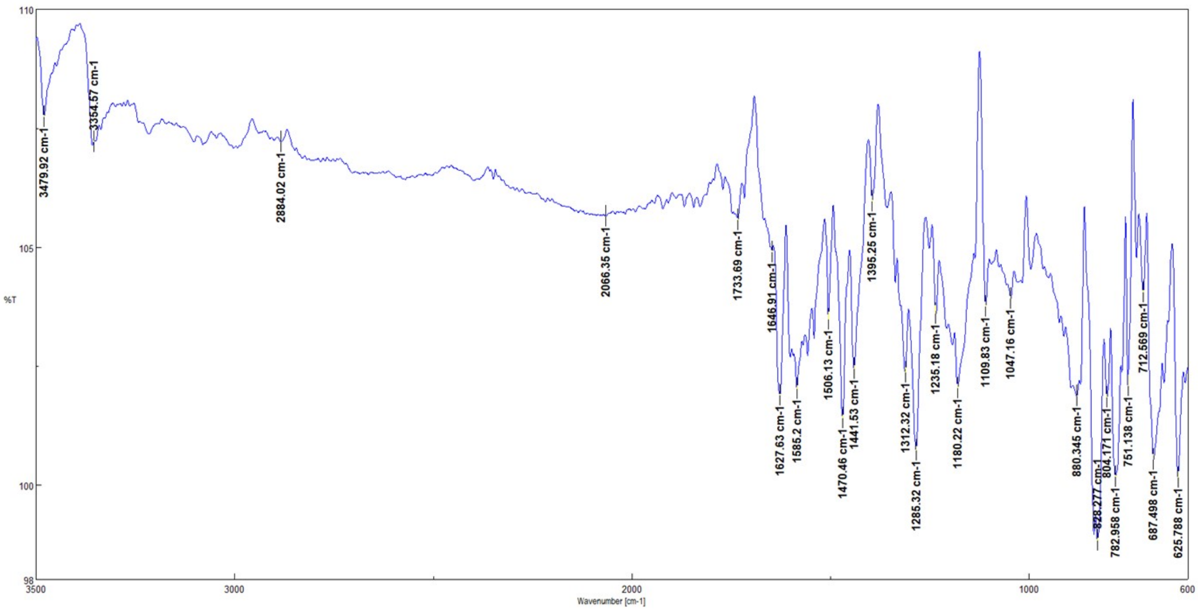

Fig. S14. FTIR Spectra of compound 5d

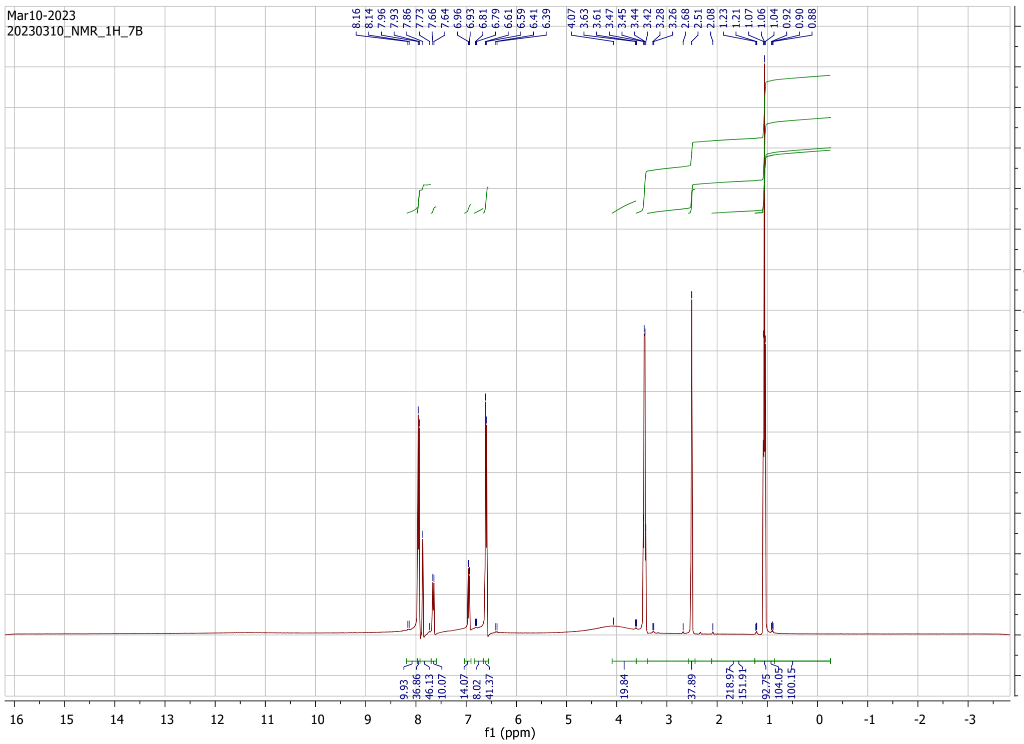

Fig. S15. ^{1}H NMR Spectra of compound 5d

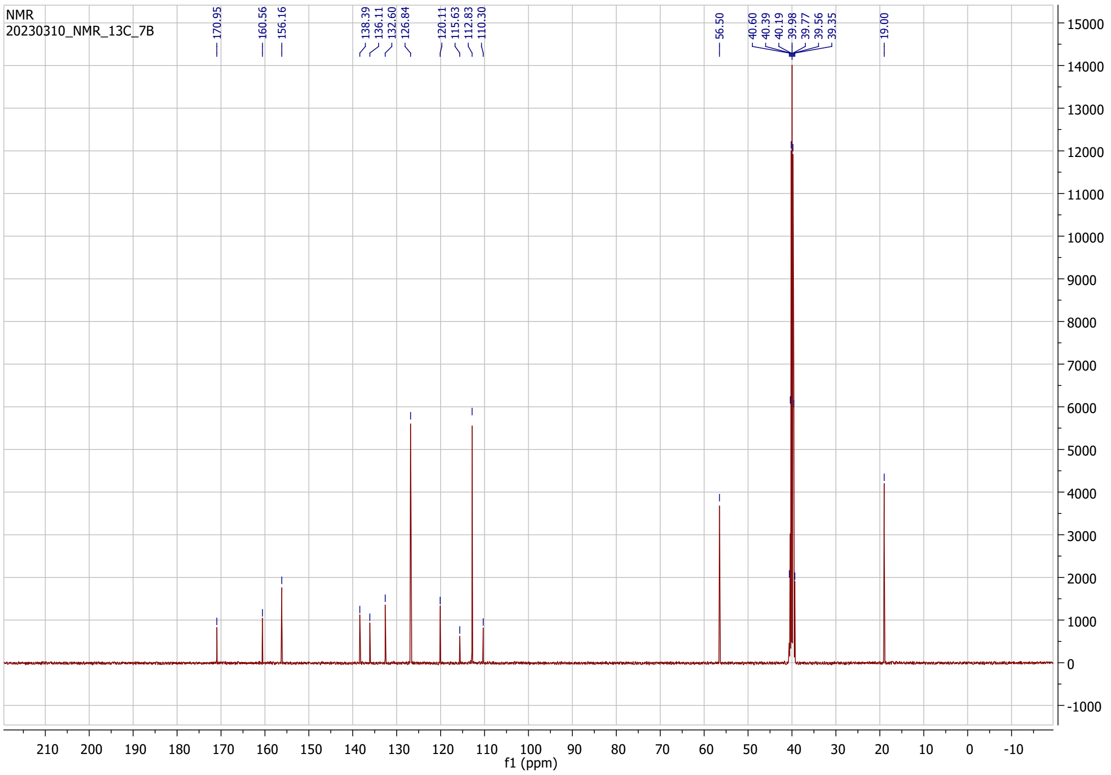

Fig. S16. ^{13}C NMR Spectra of compound 5d

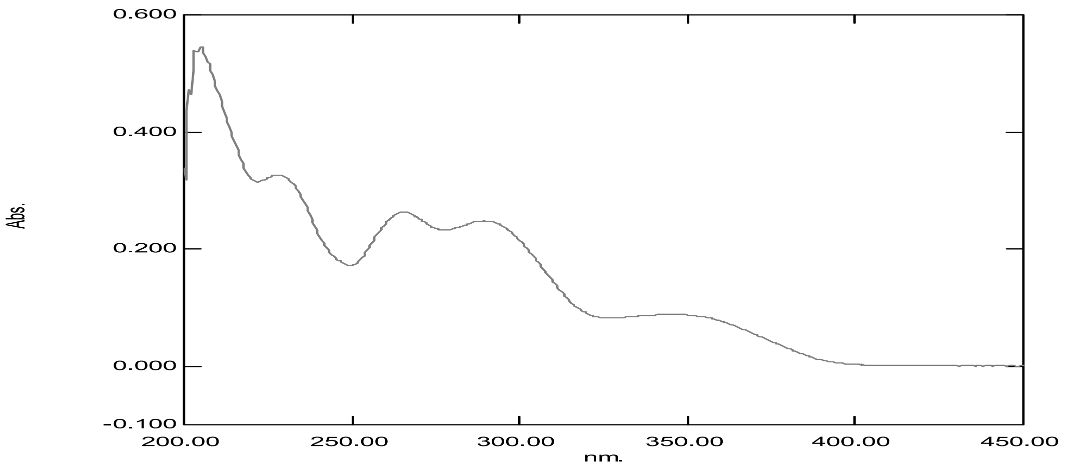

Fig. S17. UV-Visible spectra of compound 5e

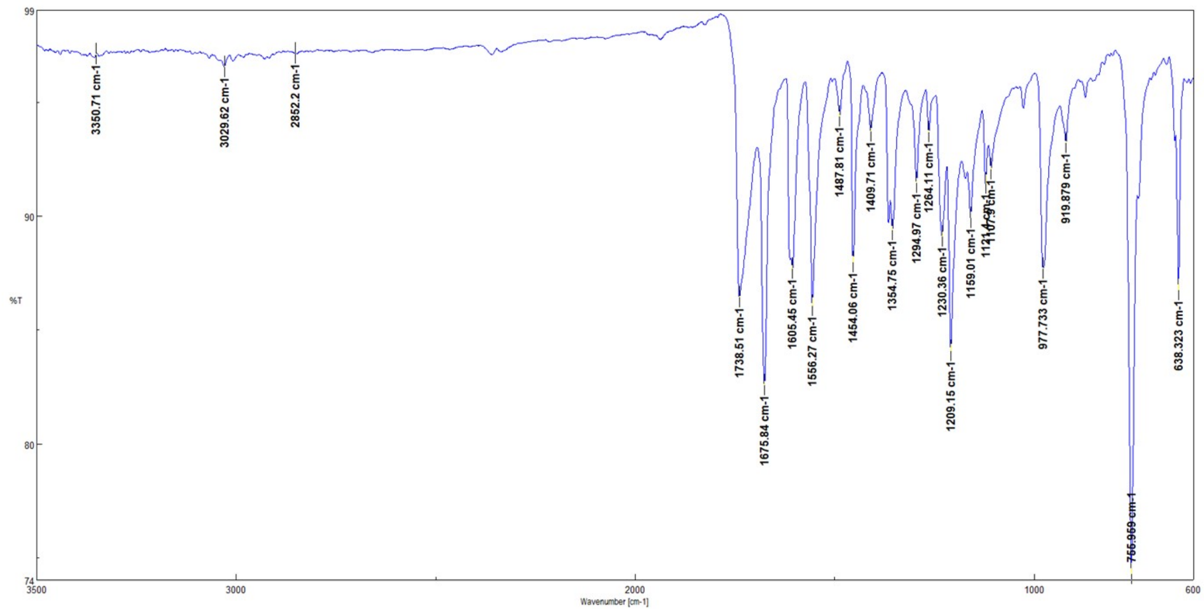

Fig. S18. FTIR Spectra of compound 5e

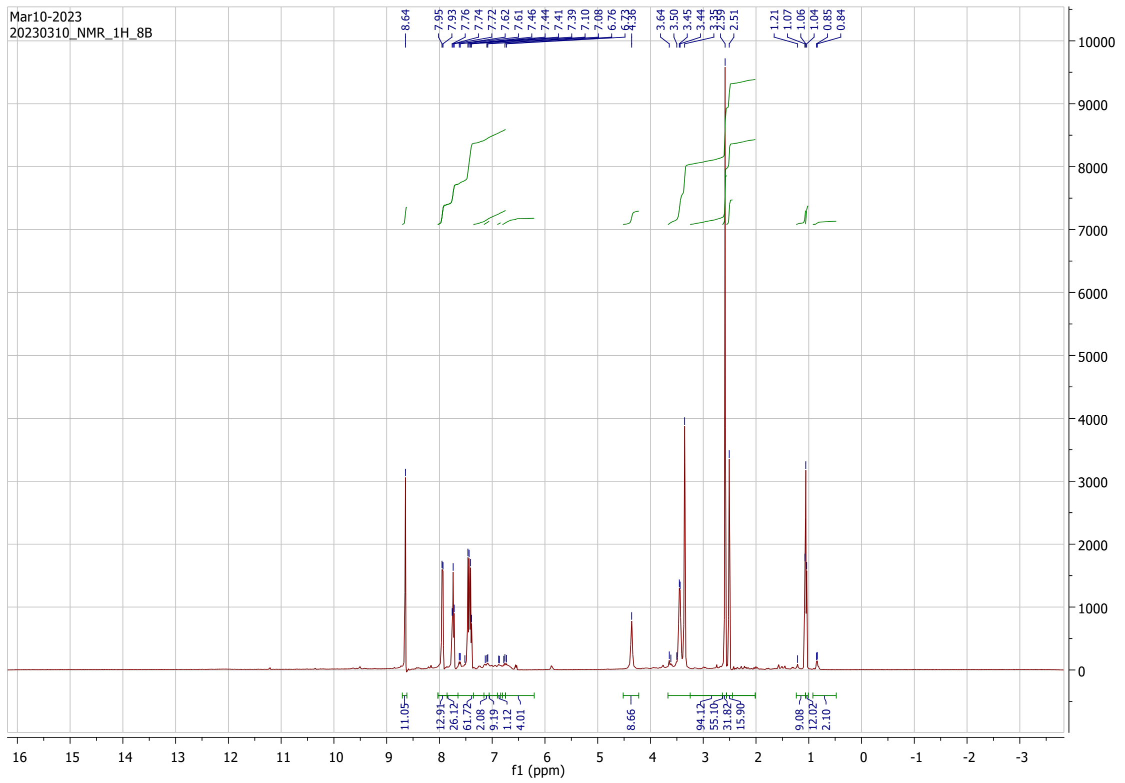

Fig. S19. ^{1}H NMR Spectra of compound 5e

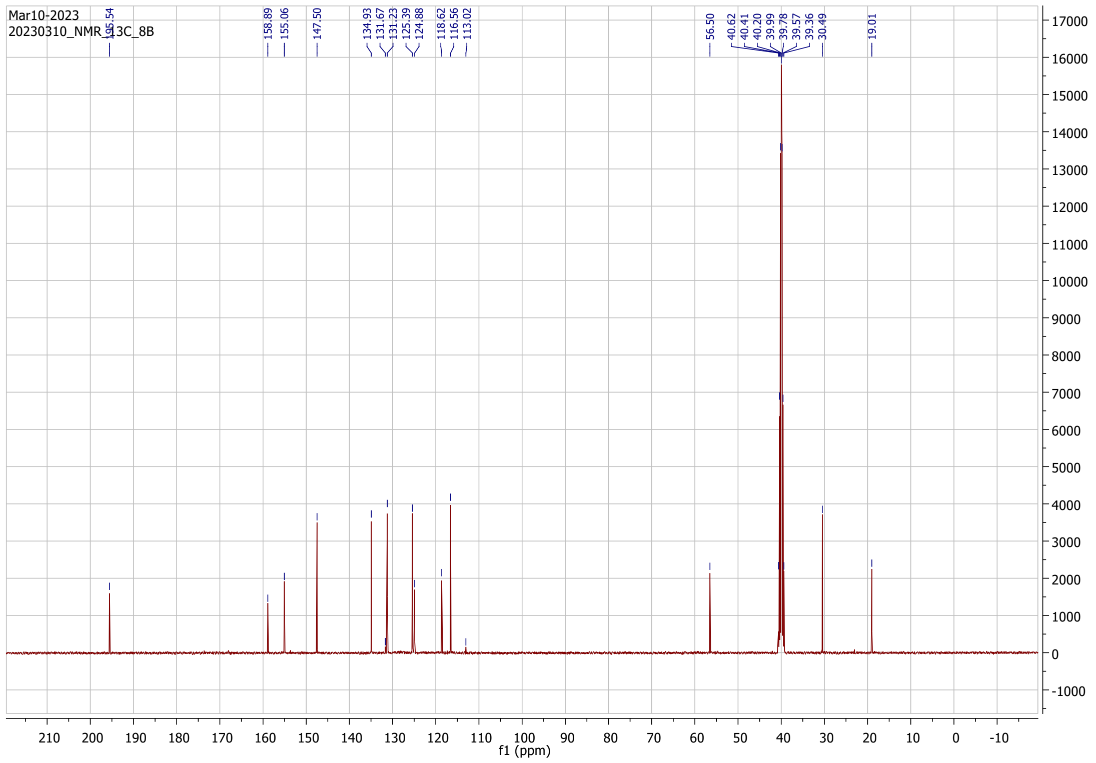

Fig. S20. ^{13}C NMR Spectra of compound 5e

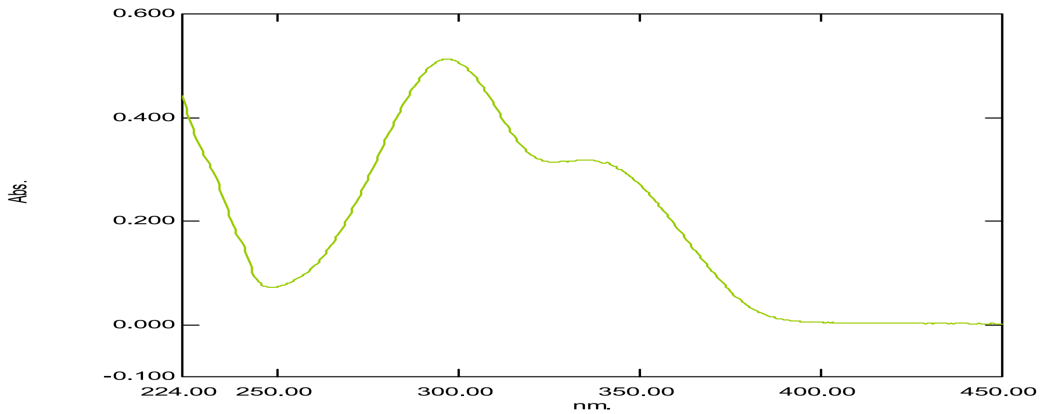

Fig. S21. UV-Visible spectra of compound 5f

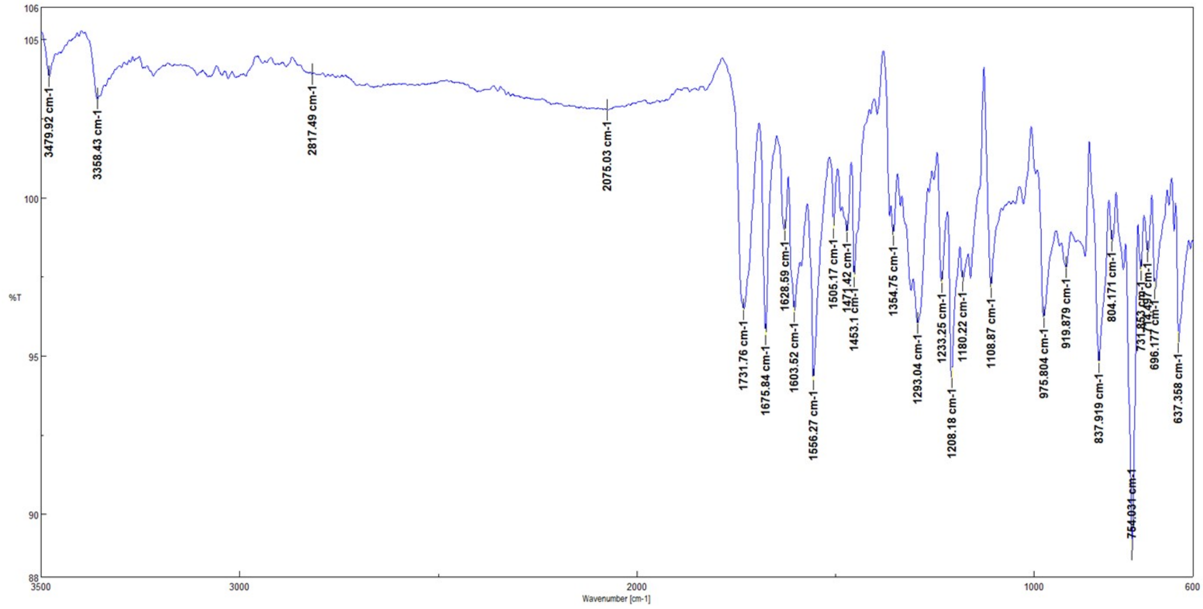

Fig. S22. FTIR Spectra of compound 5f

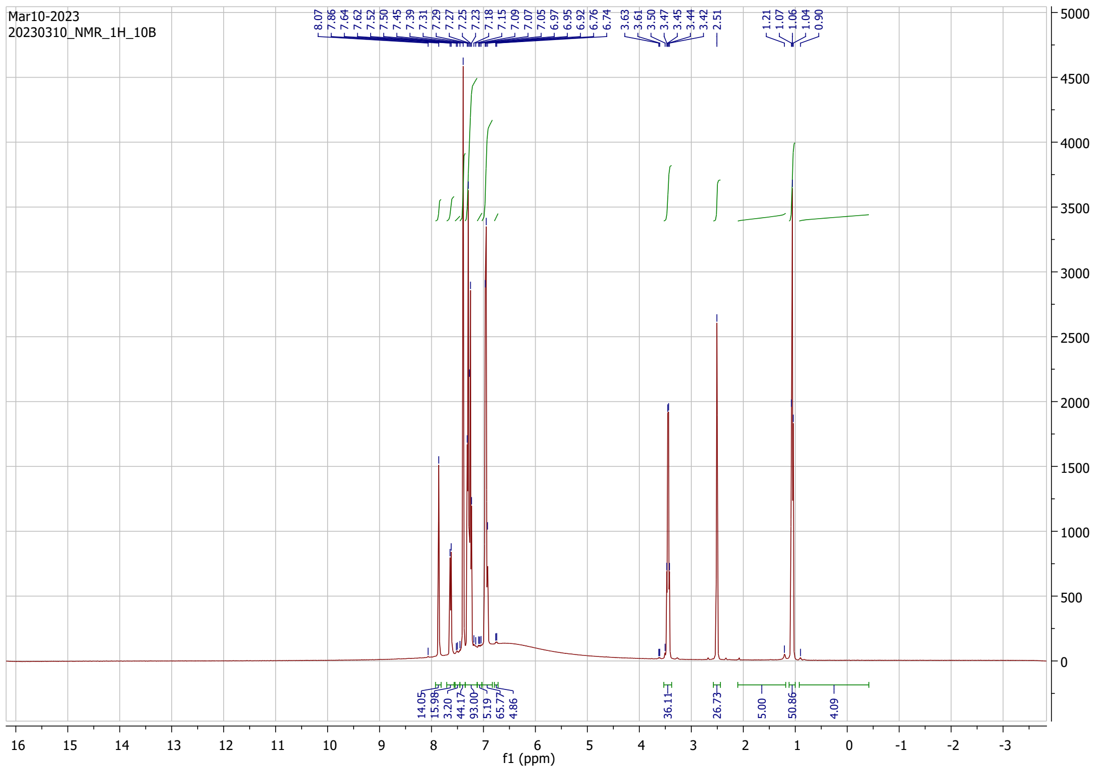

Fig. S23. ^{1}H NMR Spectra of compound 5f

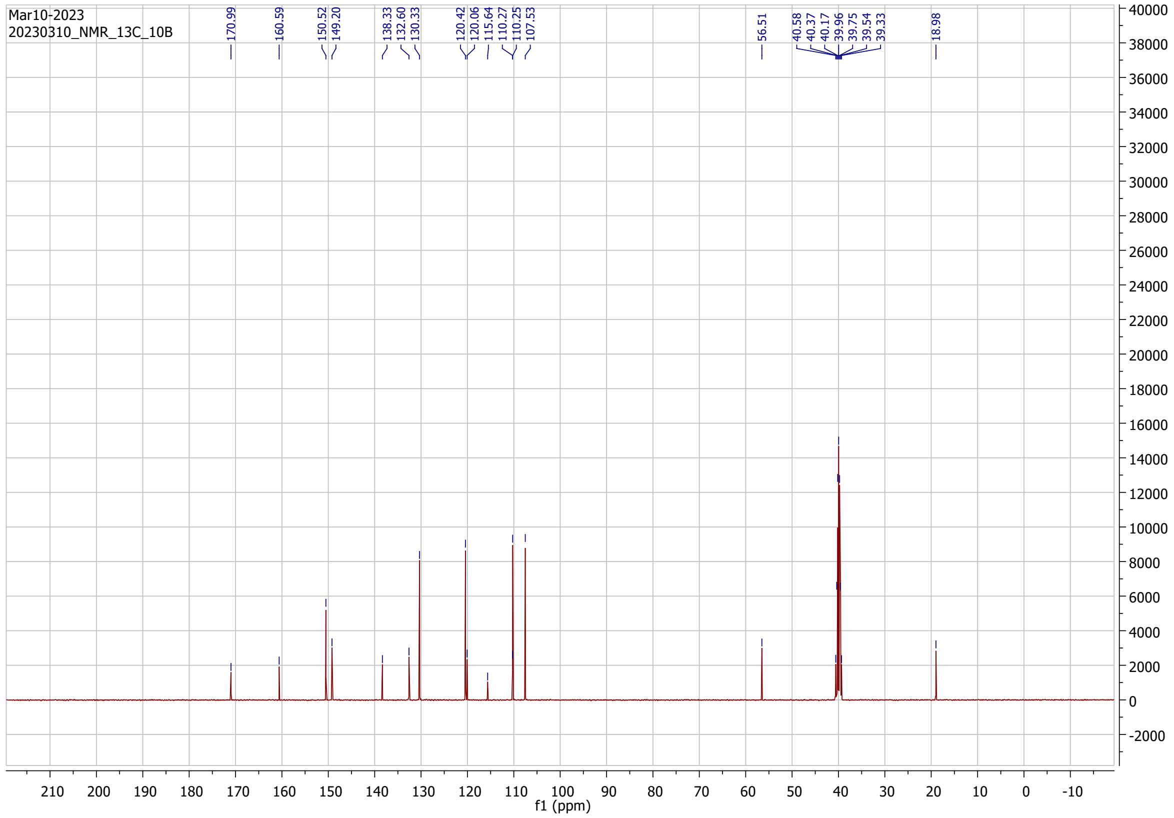

Fig. S24. ^{13}C NMR Spectra of compound 5f

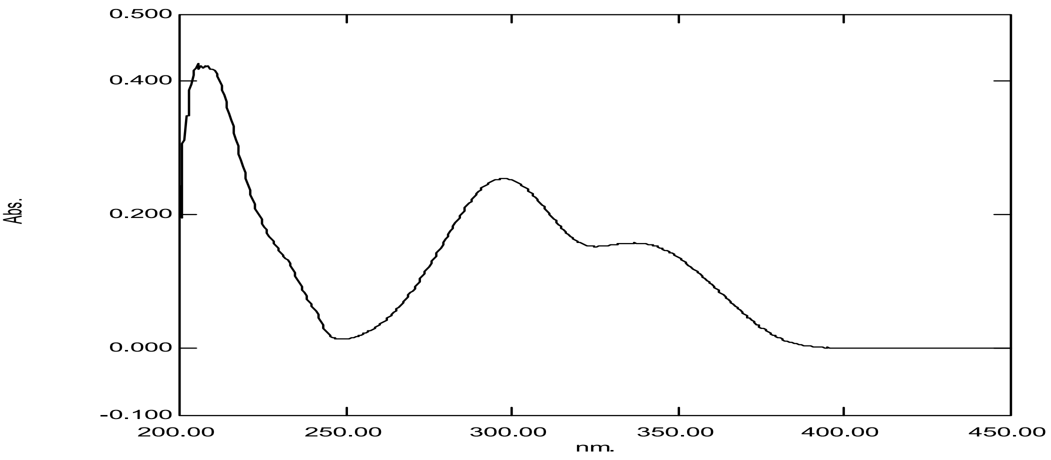

Fig. S25. UV-Visible spectra of compound 5g

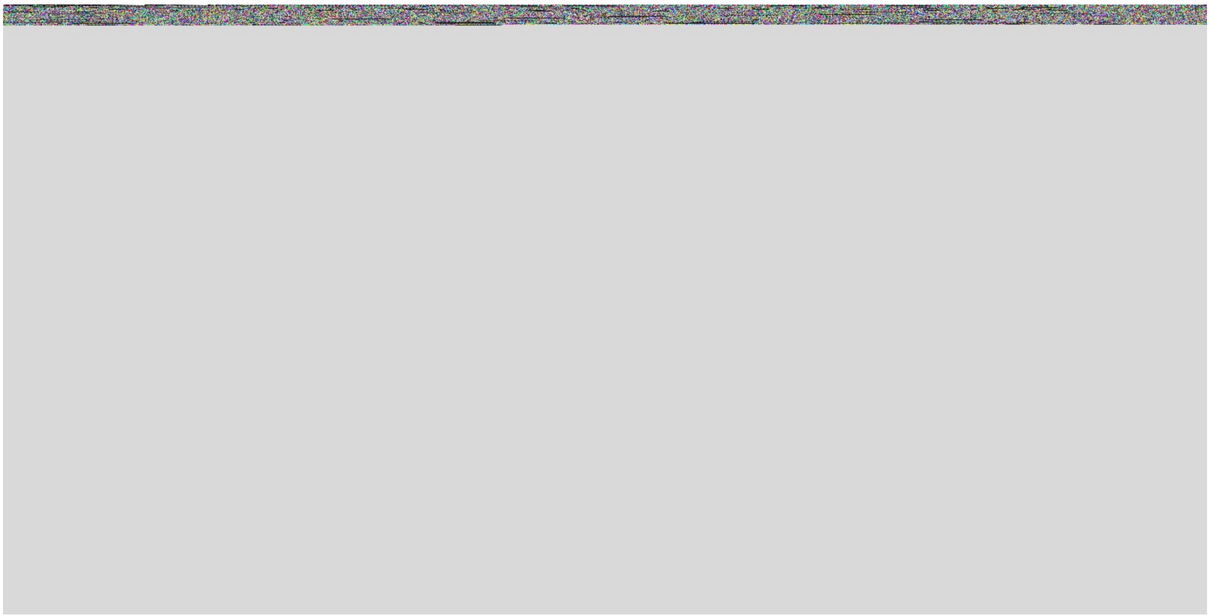

Fig. S26. FTIR Spectra of compound 5g

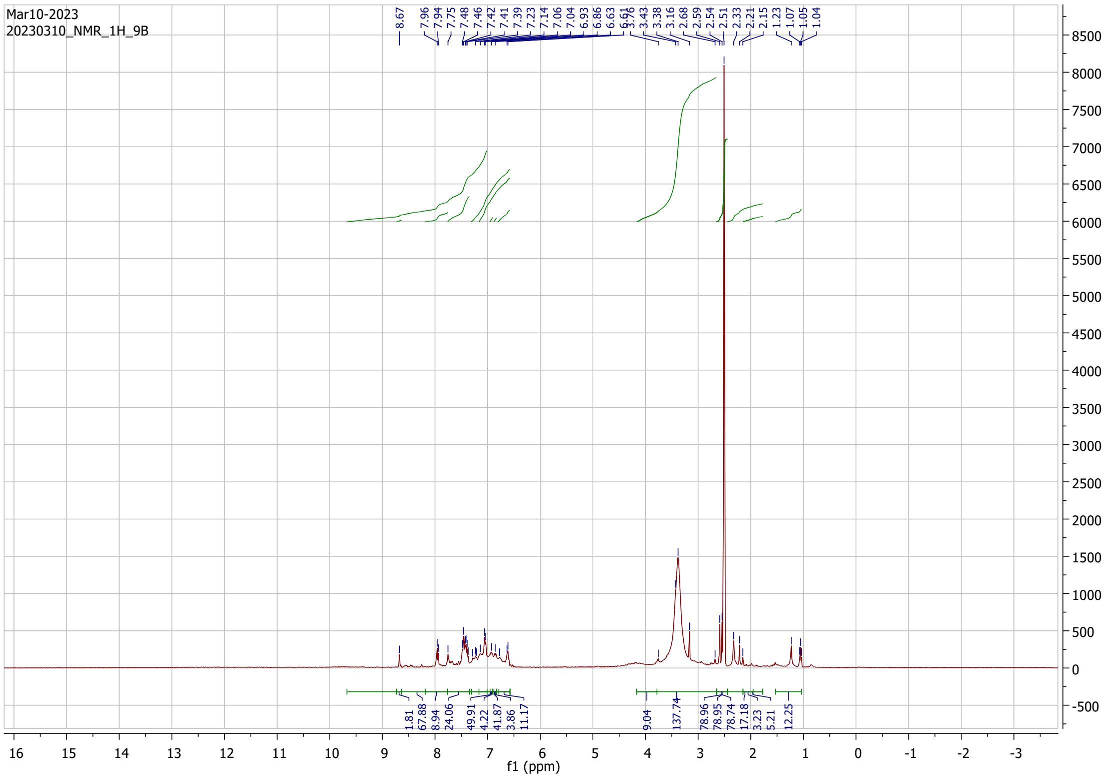

Fig. S27. ^{1}H NMR Spectra of compound 5g

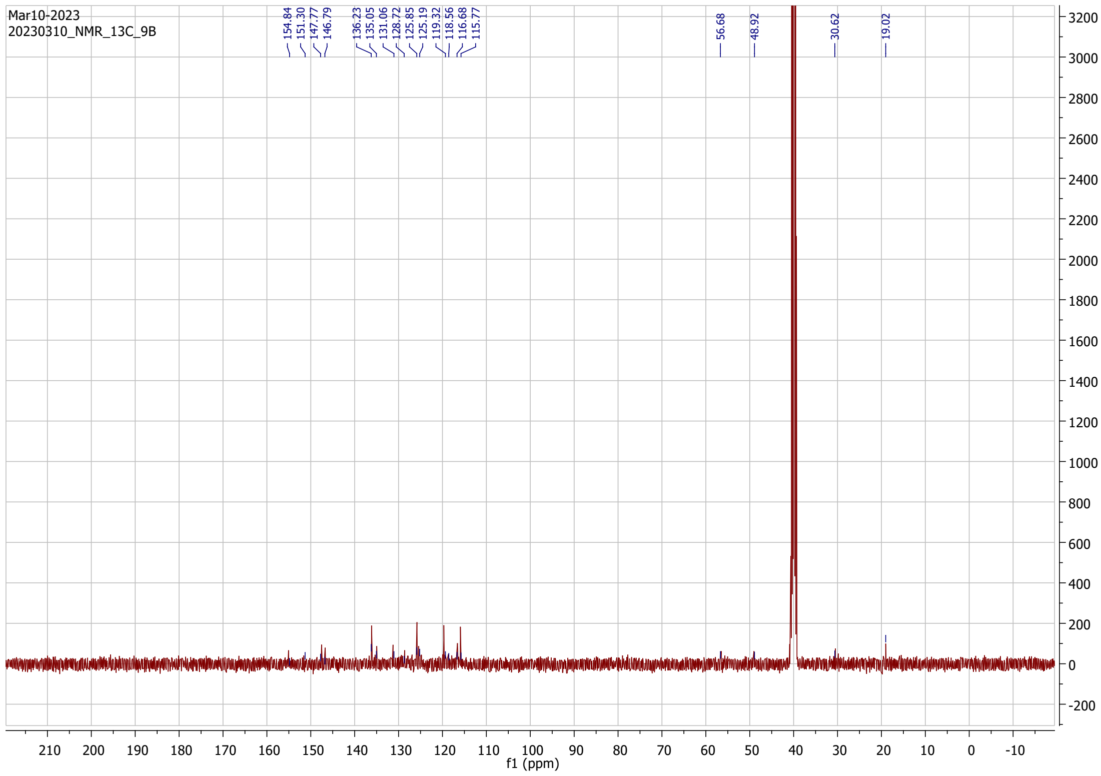

Fig. S28 ^{13}C NMR Spectra of compound 5g

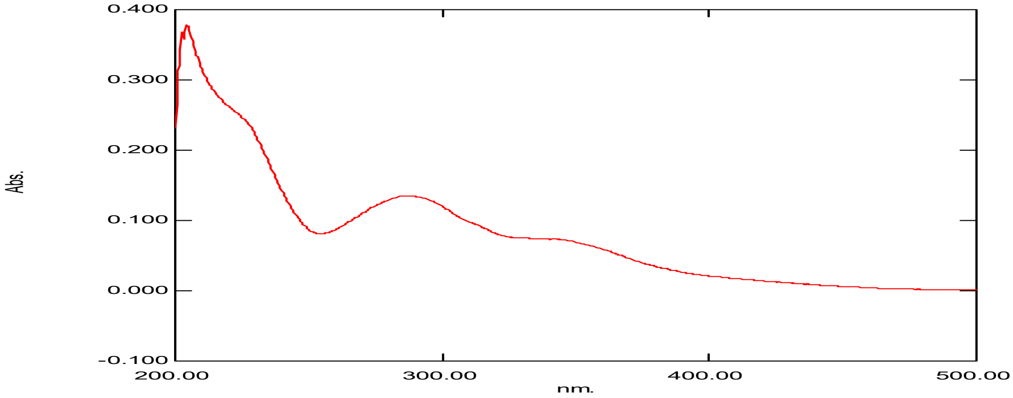

Fig. S29. UV-Visible spectra of compound 5h

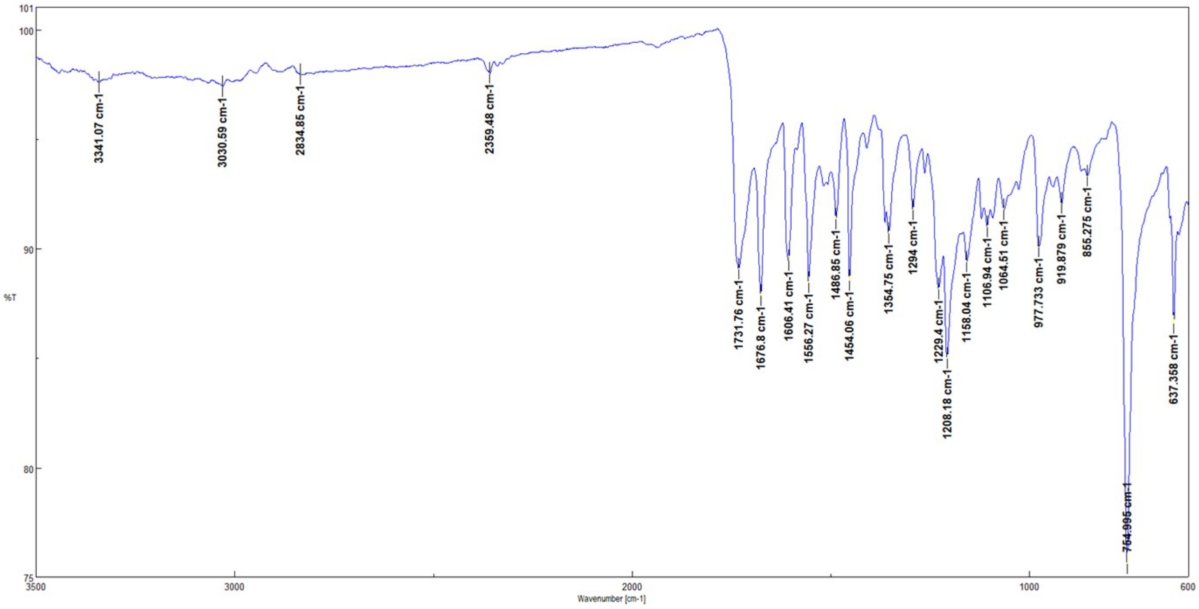

Fig. S30. FTIR Spectra of compound 5h

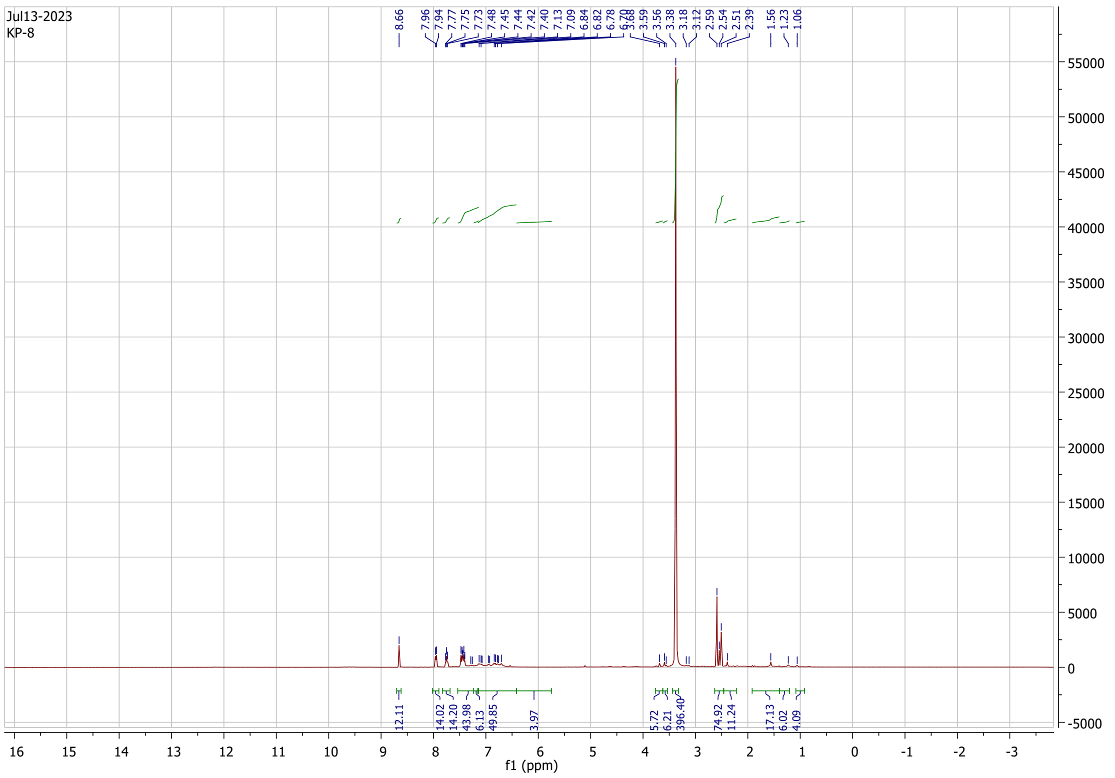

Fig. S31. ^{1}H NMR Spectra of compound 5h

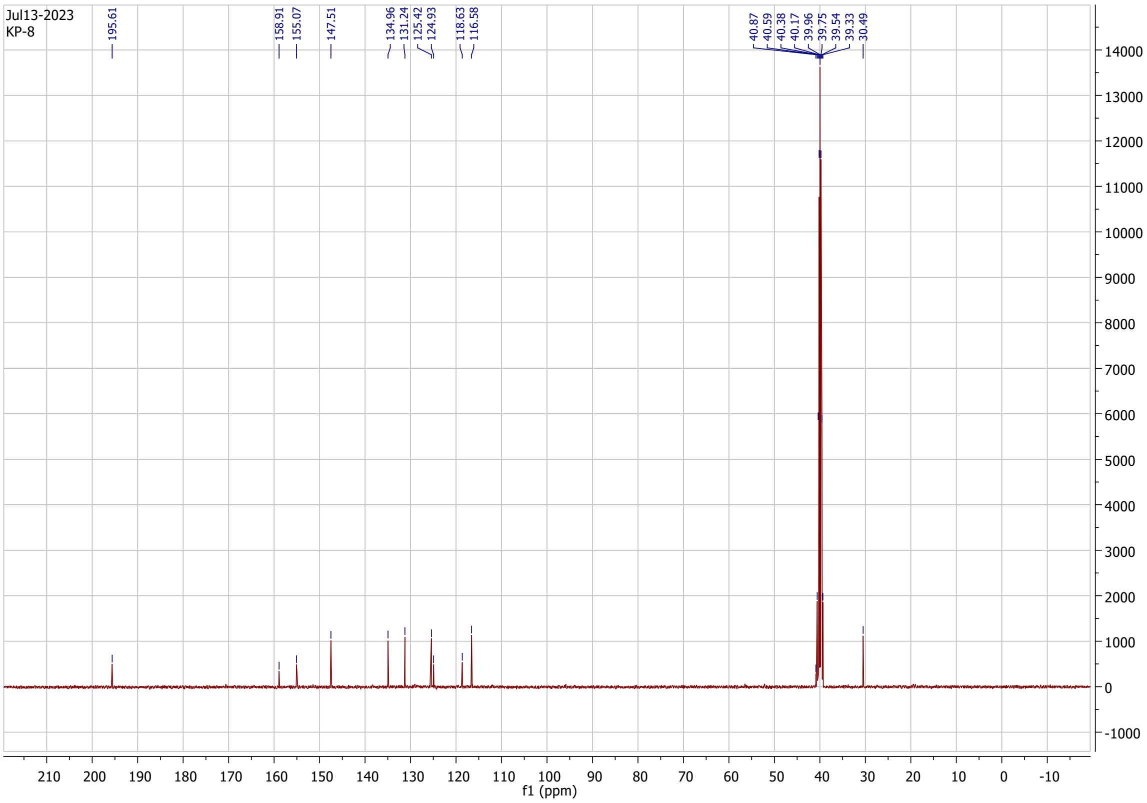

Fig. S32. ^{13}C NMR Spectra of compound 5h

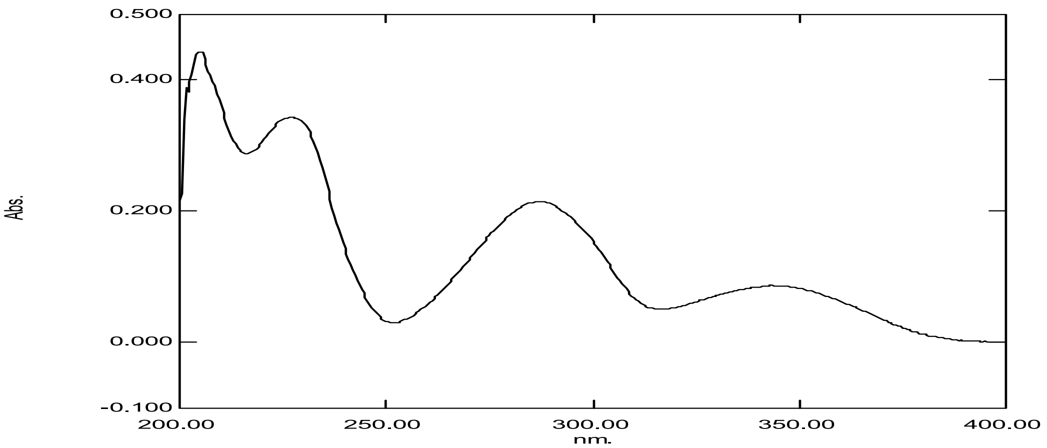

Fig. S33. UV-Visible spectra of compound 5i

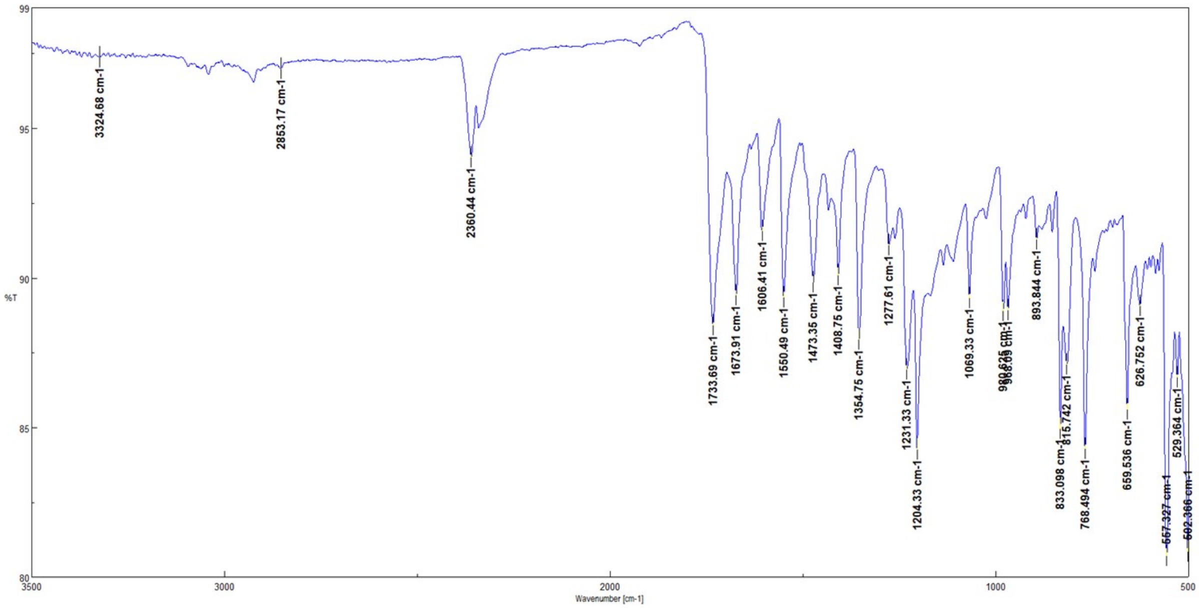

Fig. S34. FTIR Spectra of compound 5i

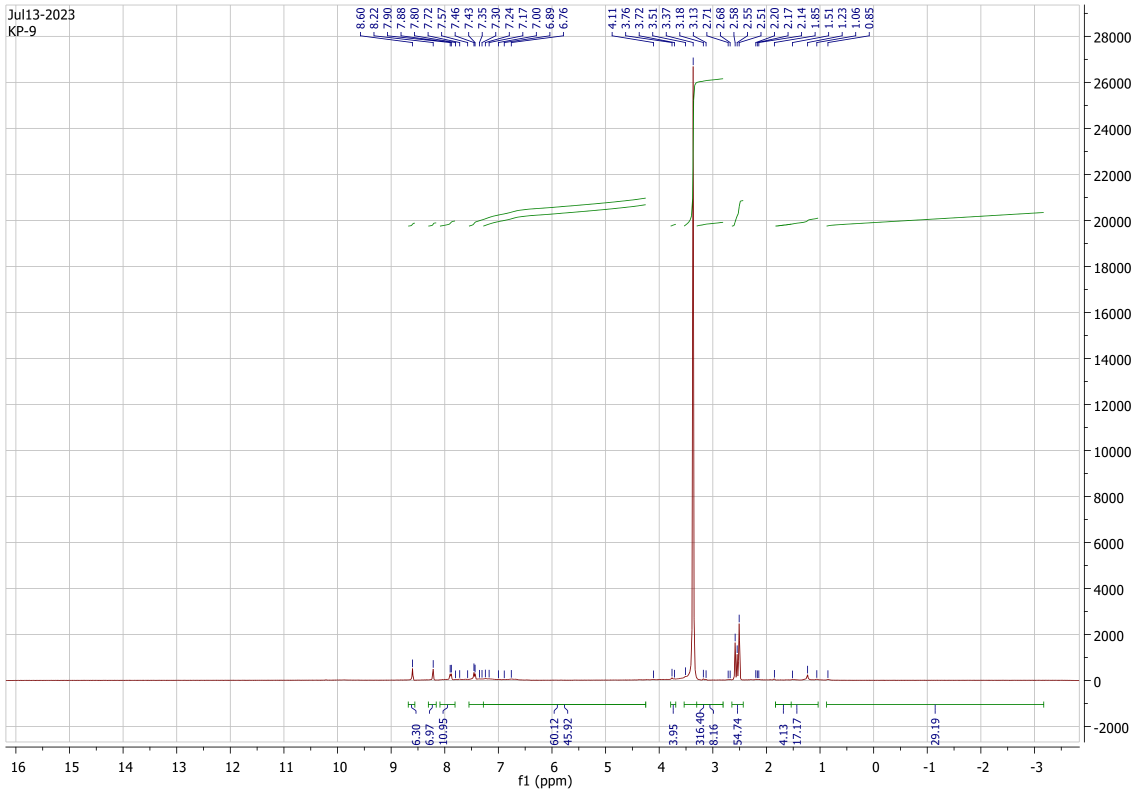

Fig. S35. ¹H NMR Spectra of compound 5i

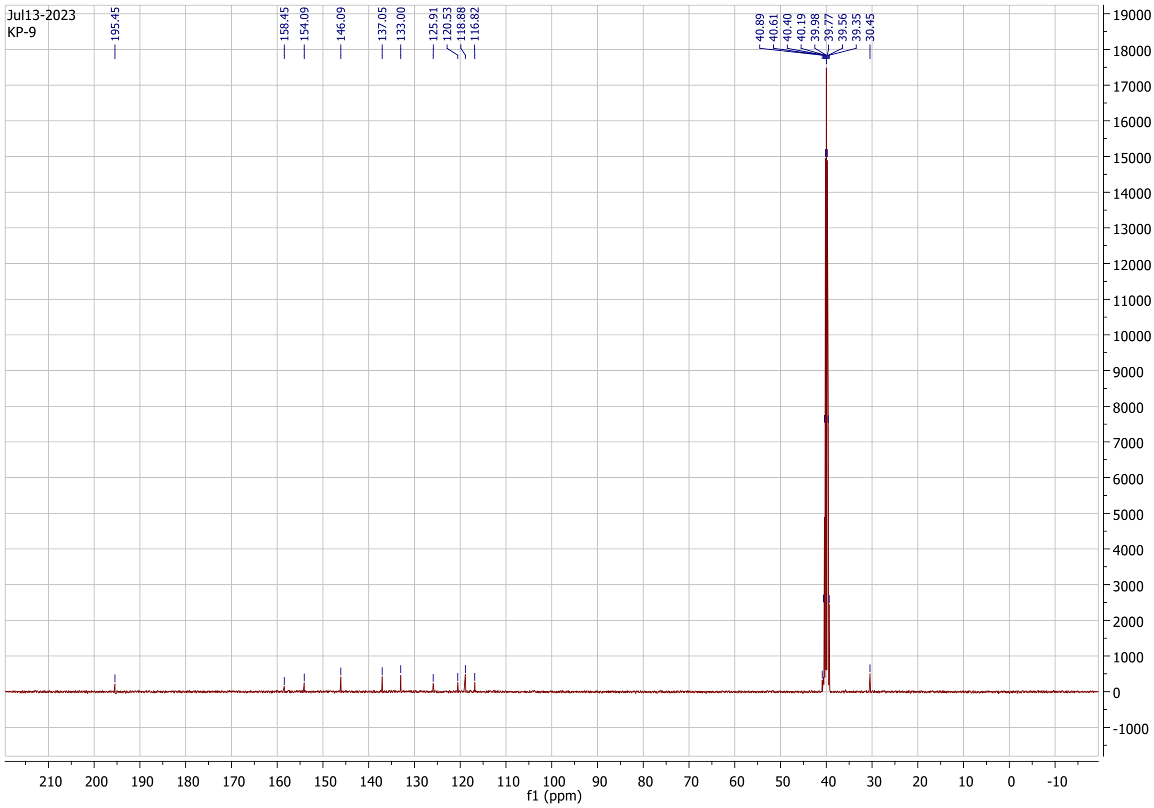

Fig. S36. ¹³C NMR Spectra of compound 5i

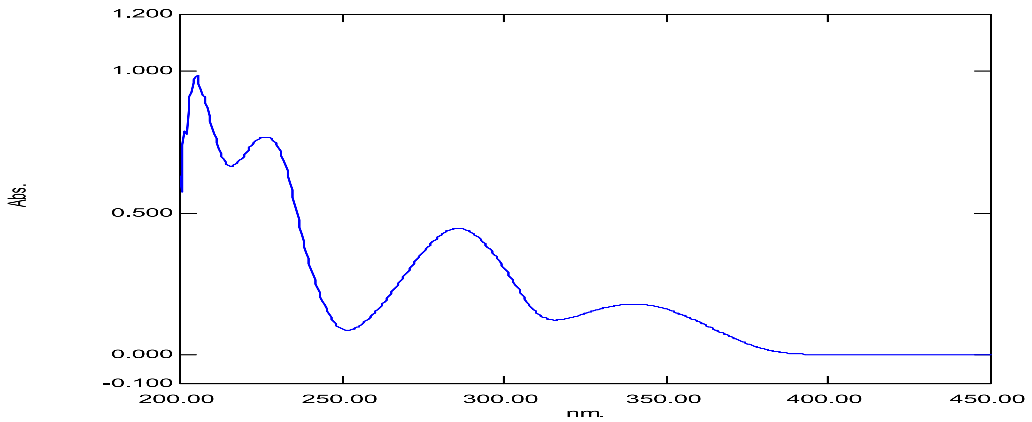

Fig. S37. UV-Visible spectra of compound 7a

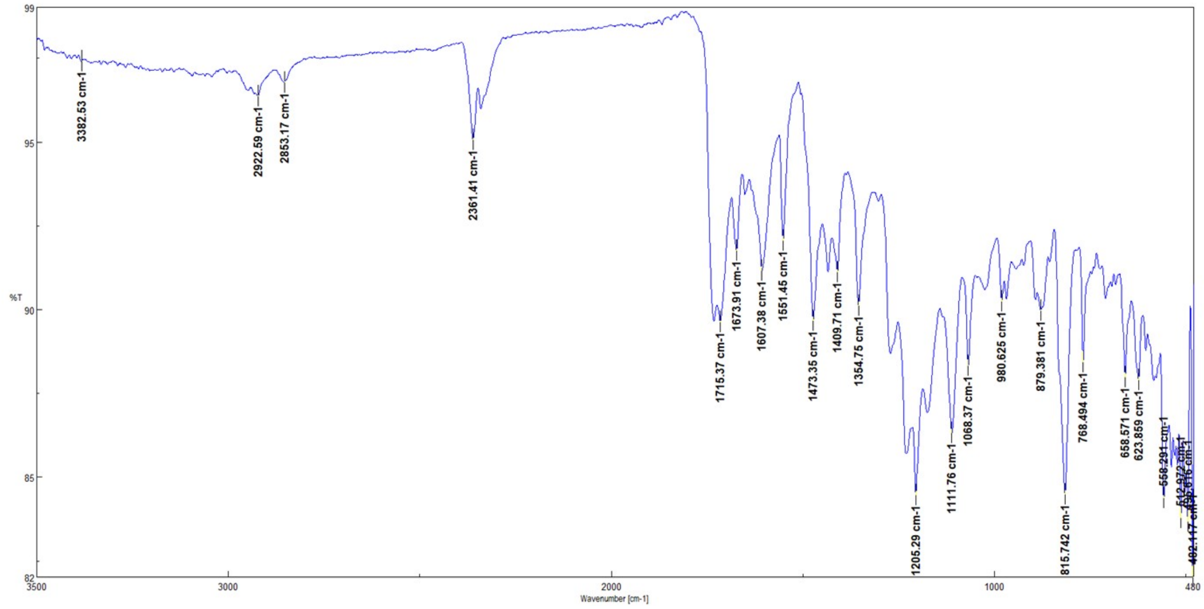

Fig. S38. FT-IR Spectra of compound 7a

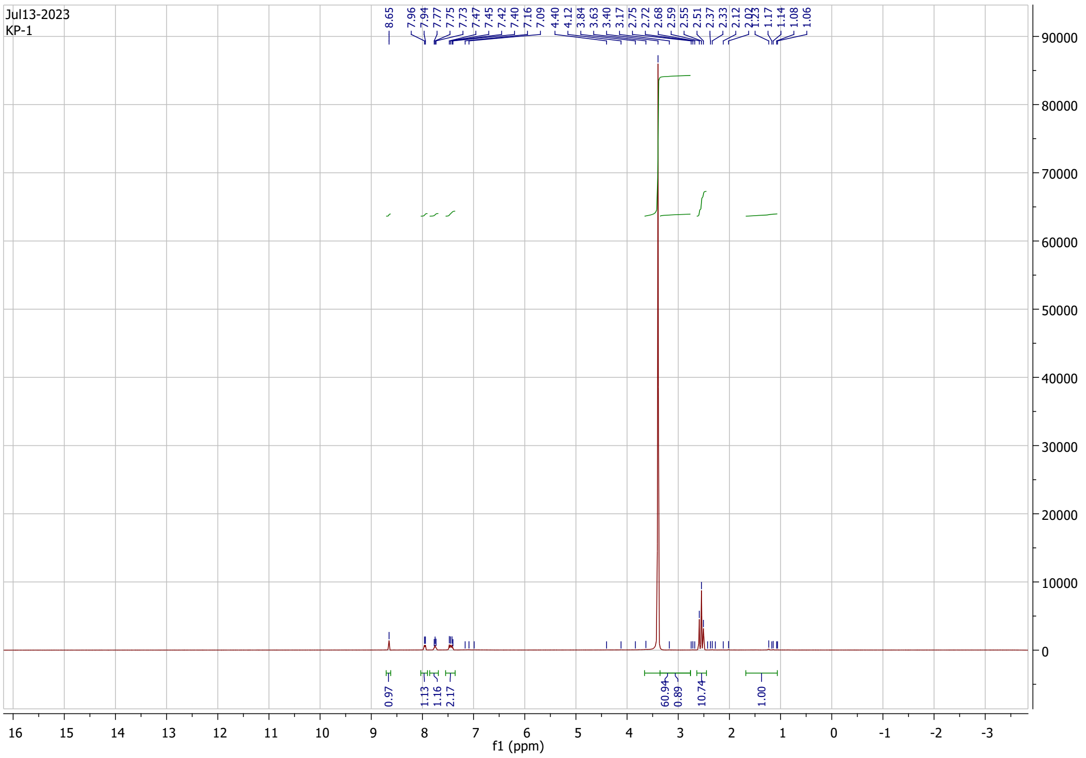

Fig. S39. ^{1}H NMR Spectra of compound 7a

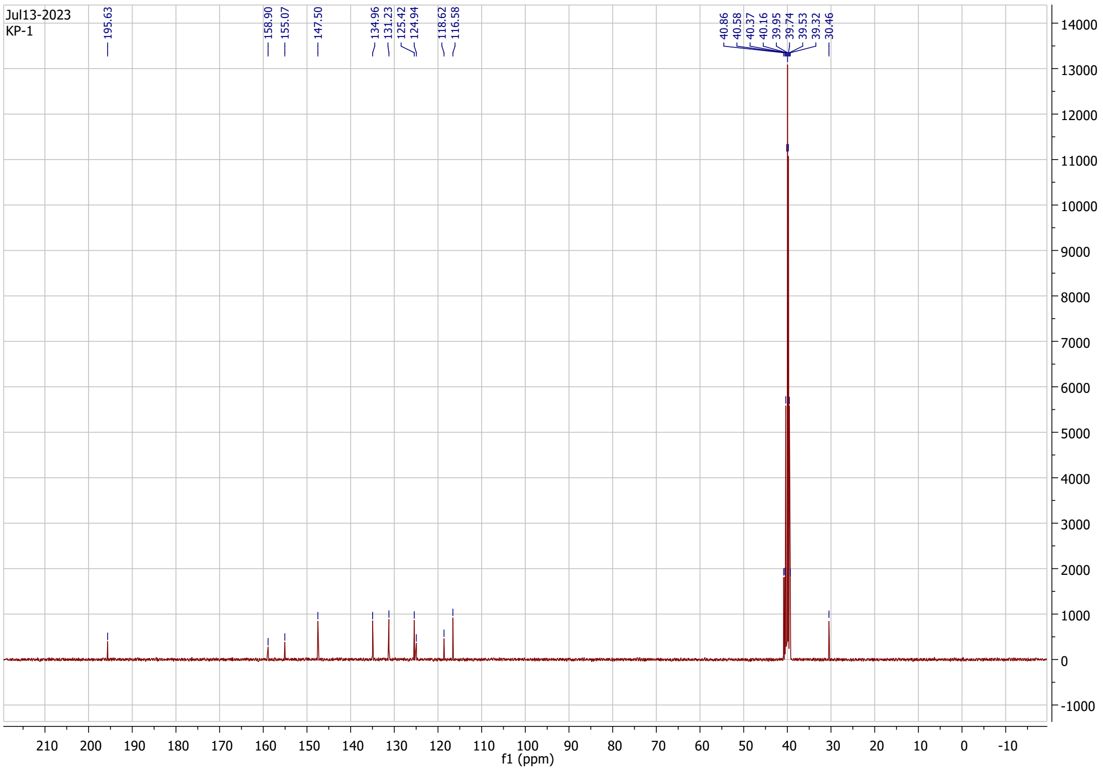

Fig. S40. ^{13}C NMR Spectra of compound 7a

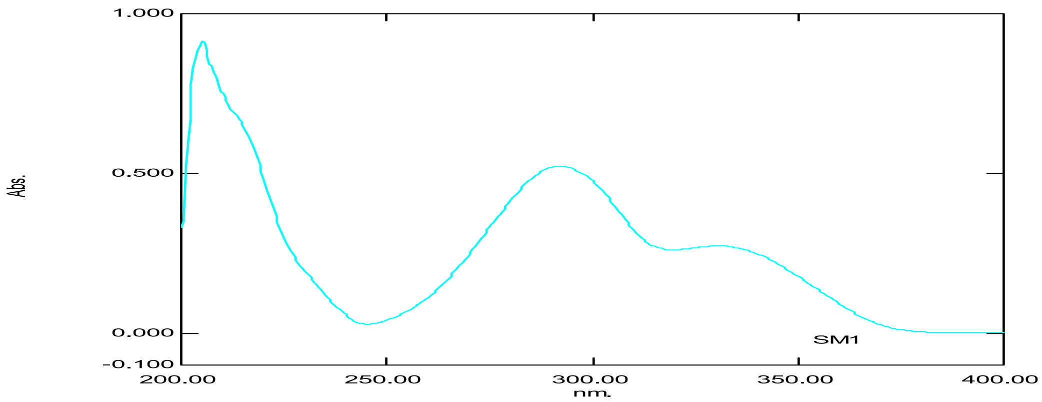

Fig. S41. UV-Visible spectra of compound 7b

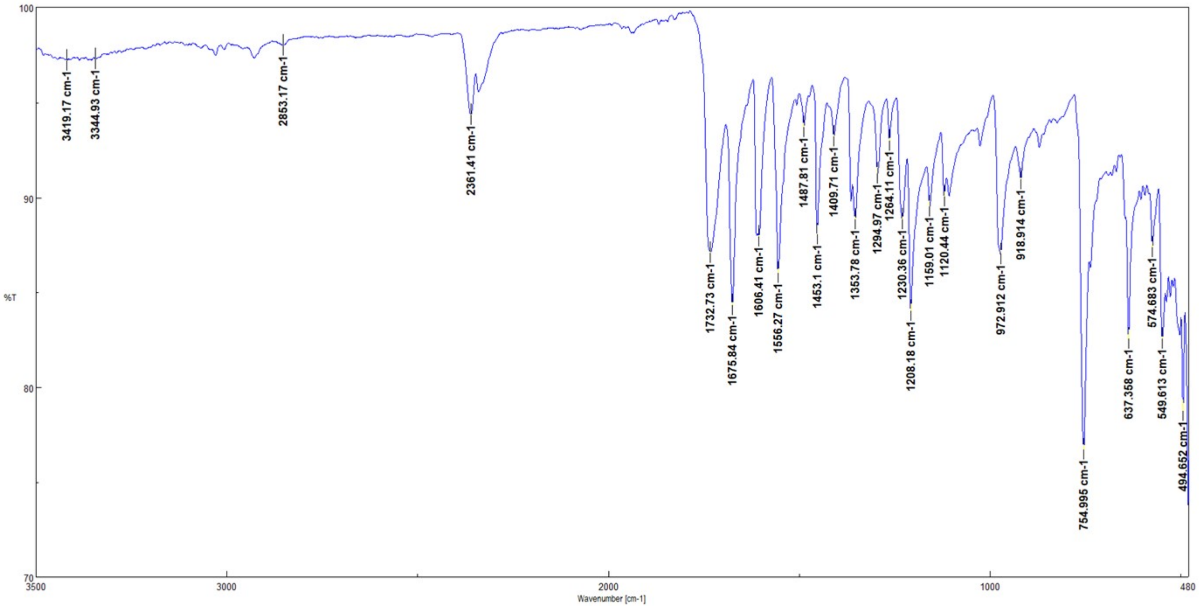

Fig. S42. FTIR Spectra of compound 7b

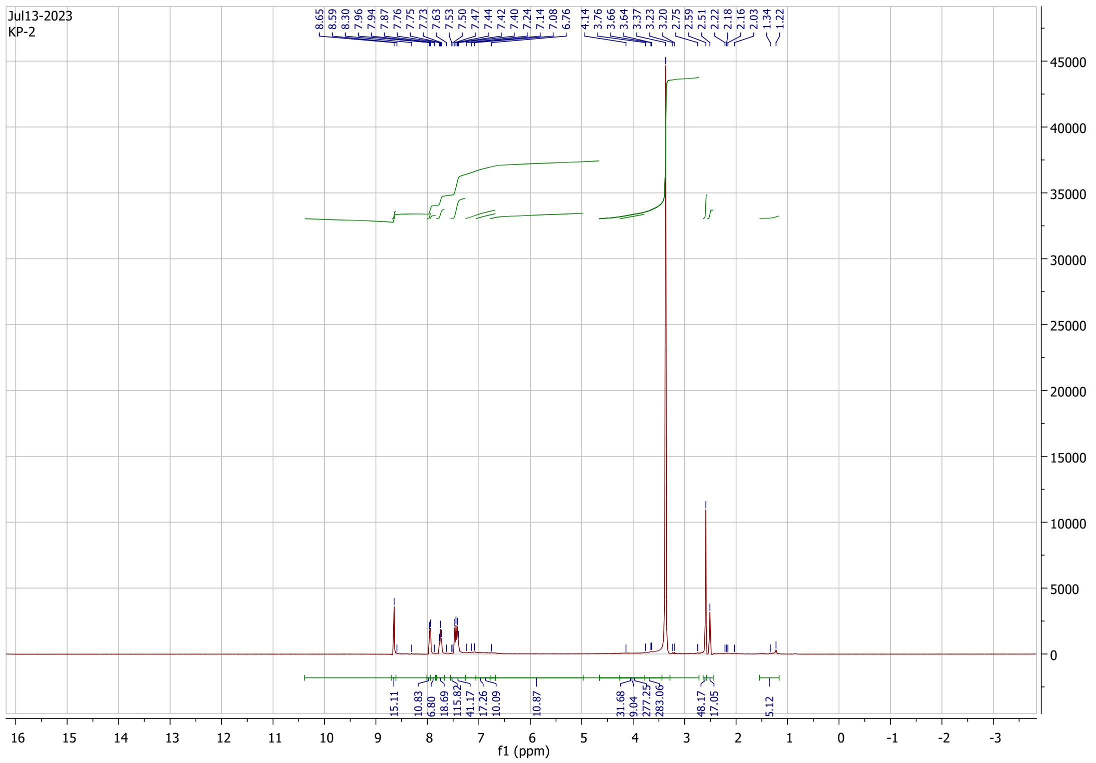

Fig. S43. ^{1}H NMR Spectra of compound 7b

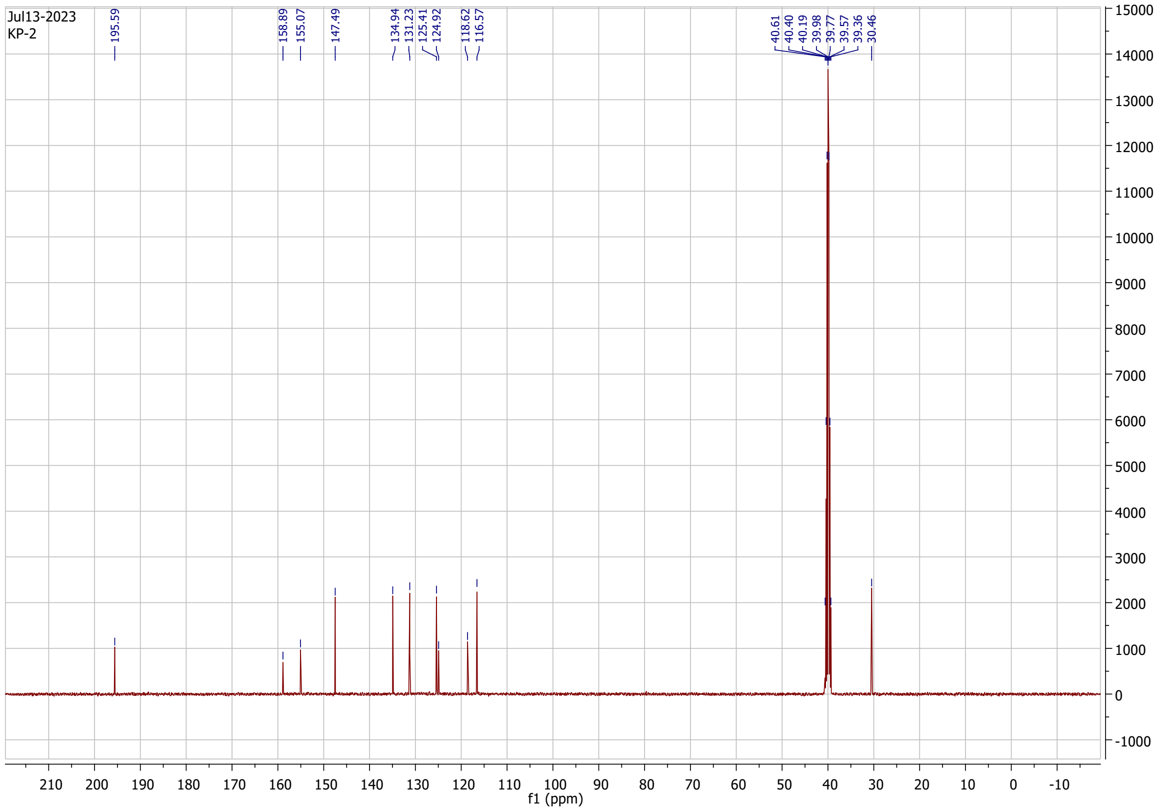

Fig. S44. ^{13}C NMR Spectra of compound 7b

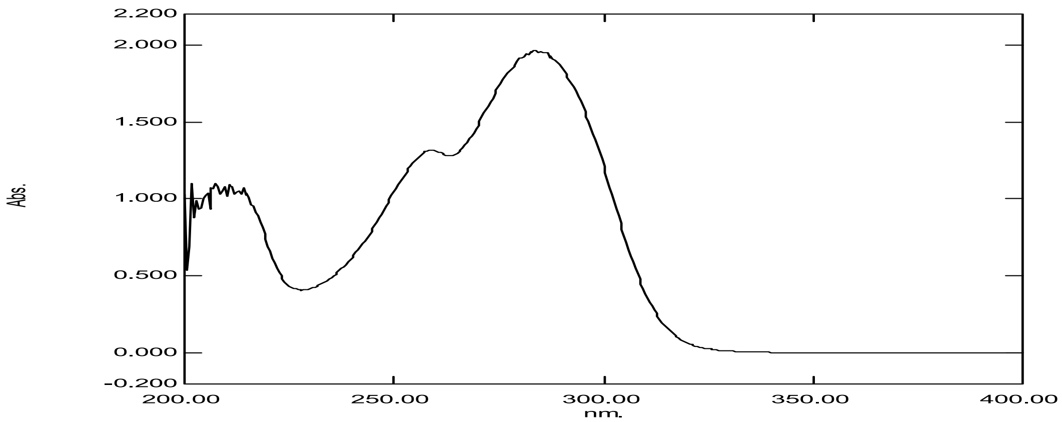

Fig. S45. UV-Visible spectra of compound 7c

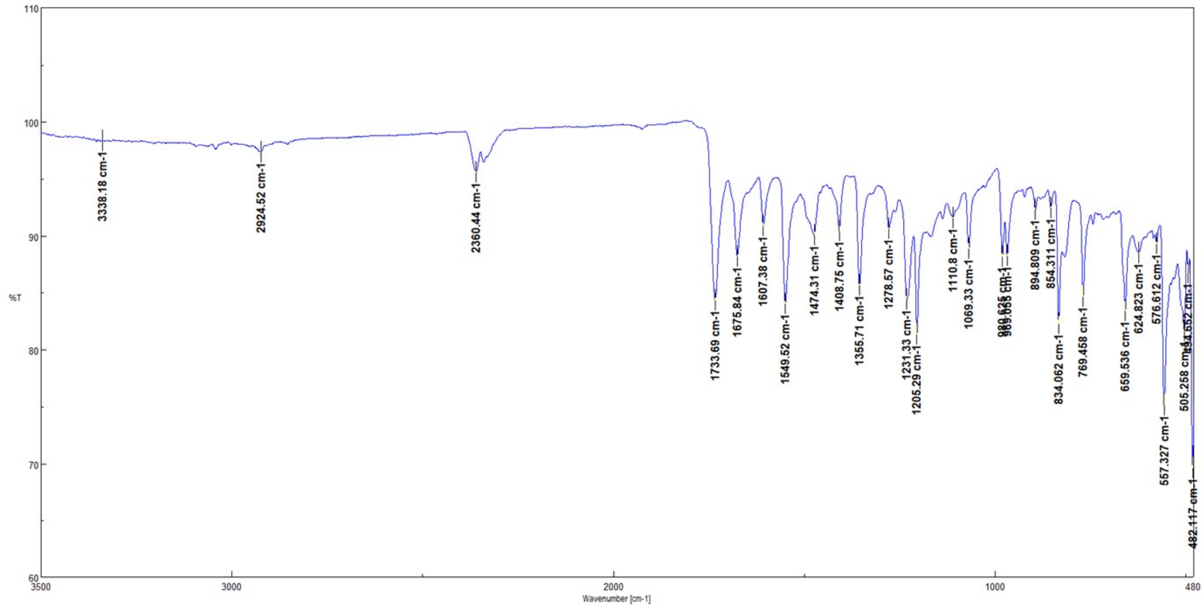

Fig. S46. FTIR Spectra of compound 7c

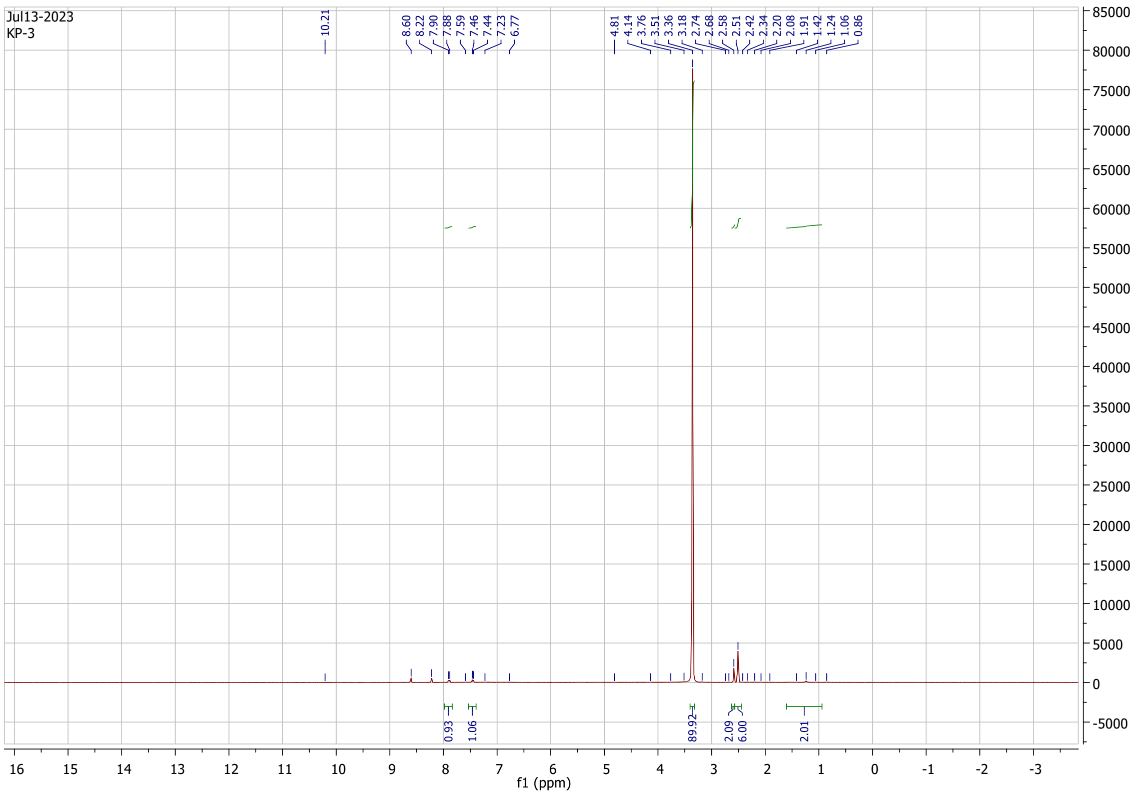

Fig. S47. ^{1}H NMR Spectra of compound 7c

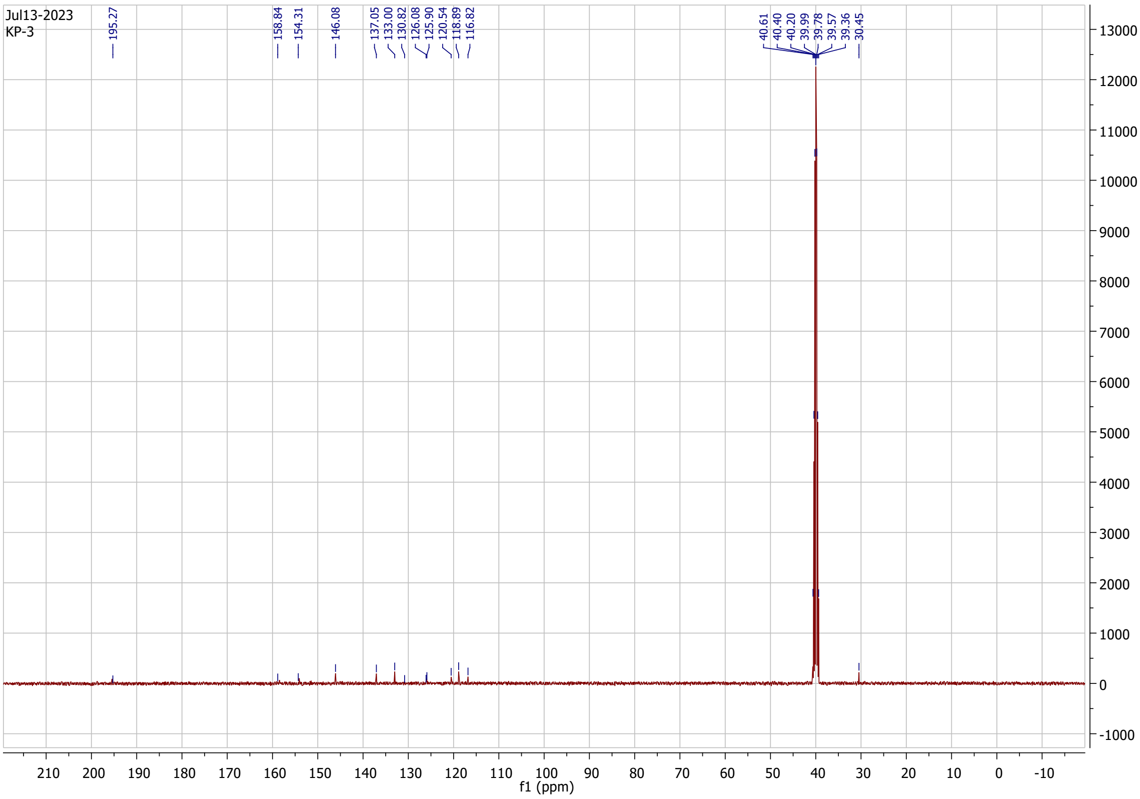

Fig. S48. ^{13}C NMR Spectra of compound 7c

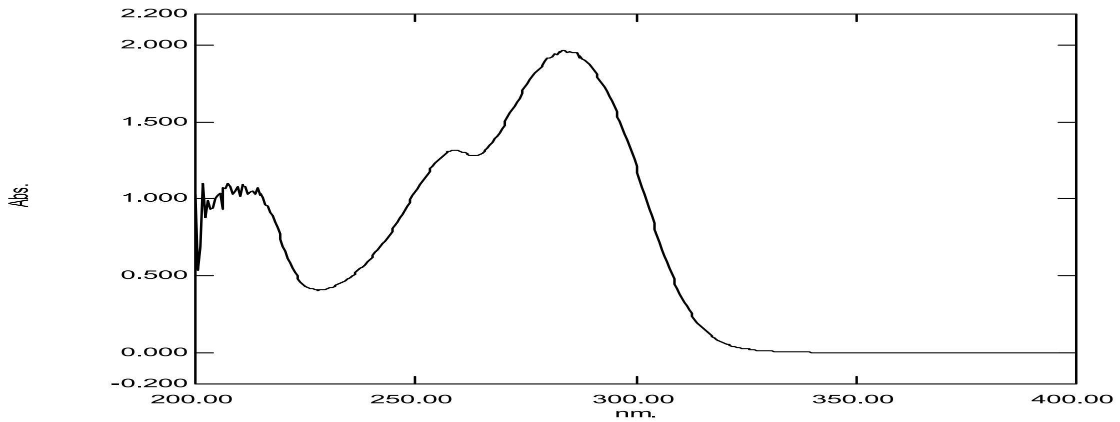

Fig. S49. UV-Visible spectra of compound 7d

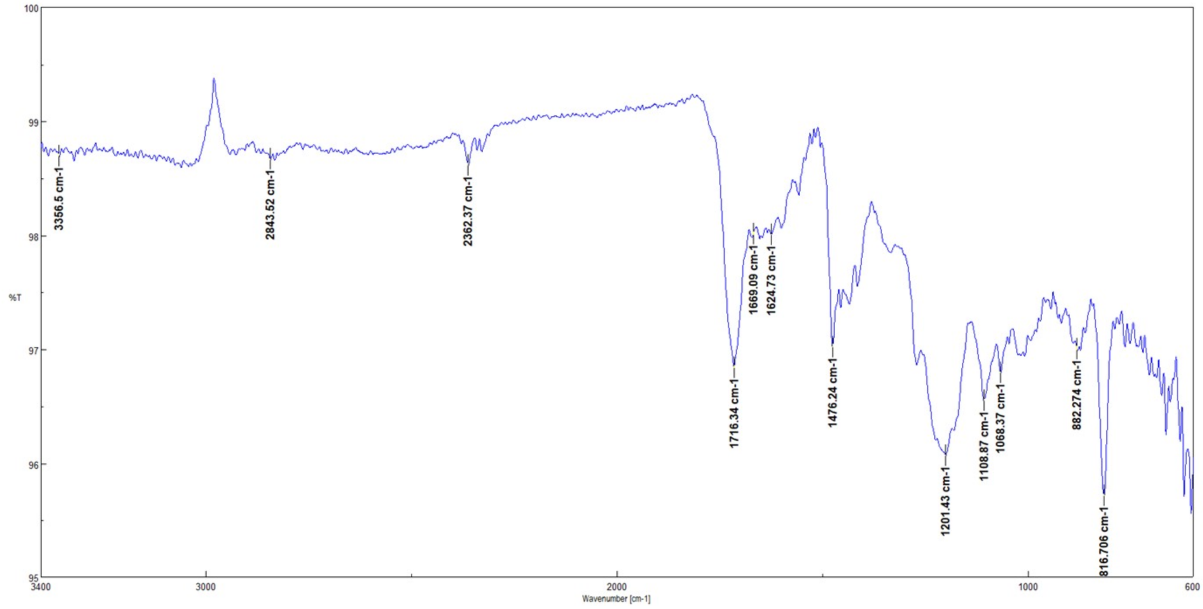

Fig. S50. FTIR Spectra of compound 7d

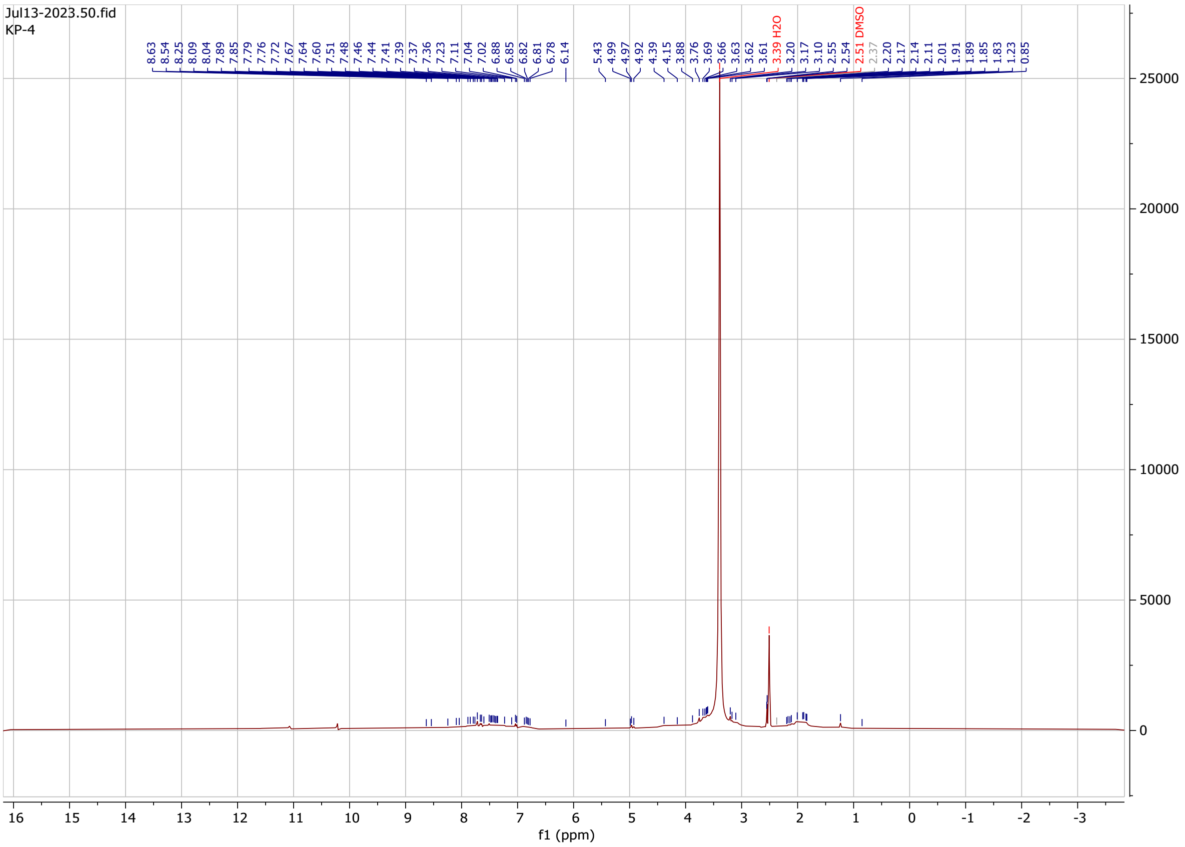

Fig. S51. ^{1}H NMR Spectra of compound 7d

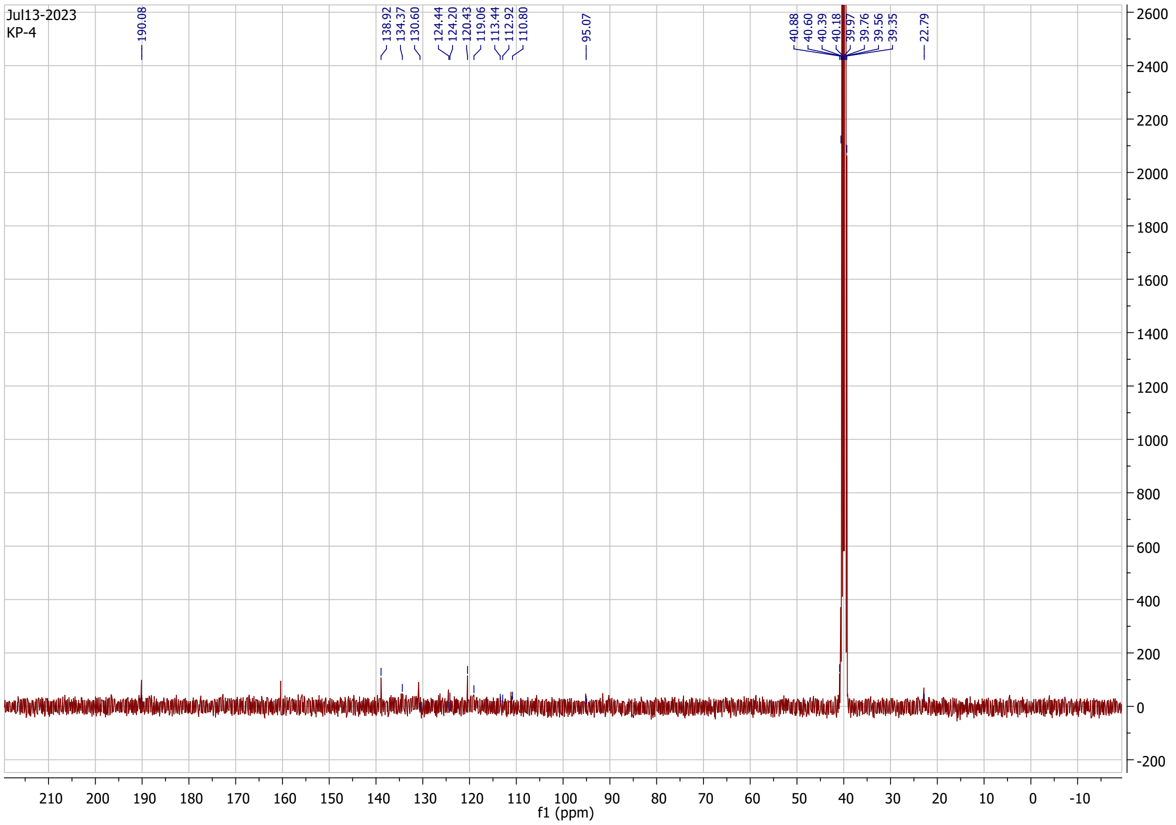

Fig. S52. ^{13}C NMR Spectra of compound 7d

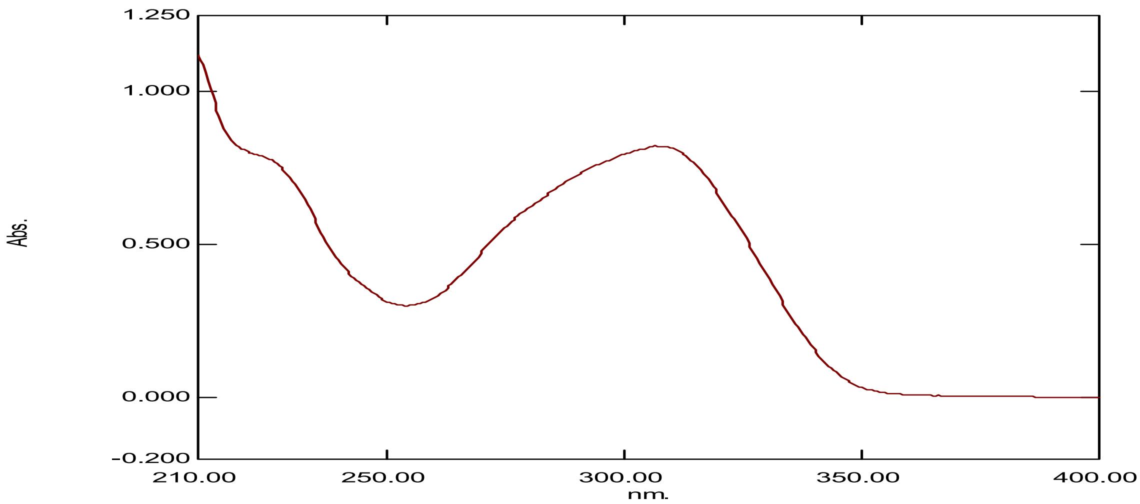

Fig. S53. UV-Visible spectra of compound 7e

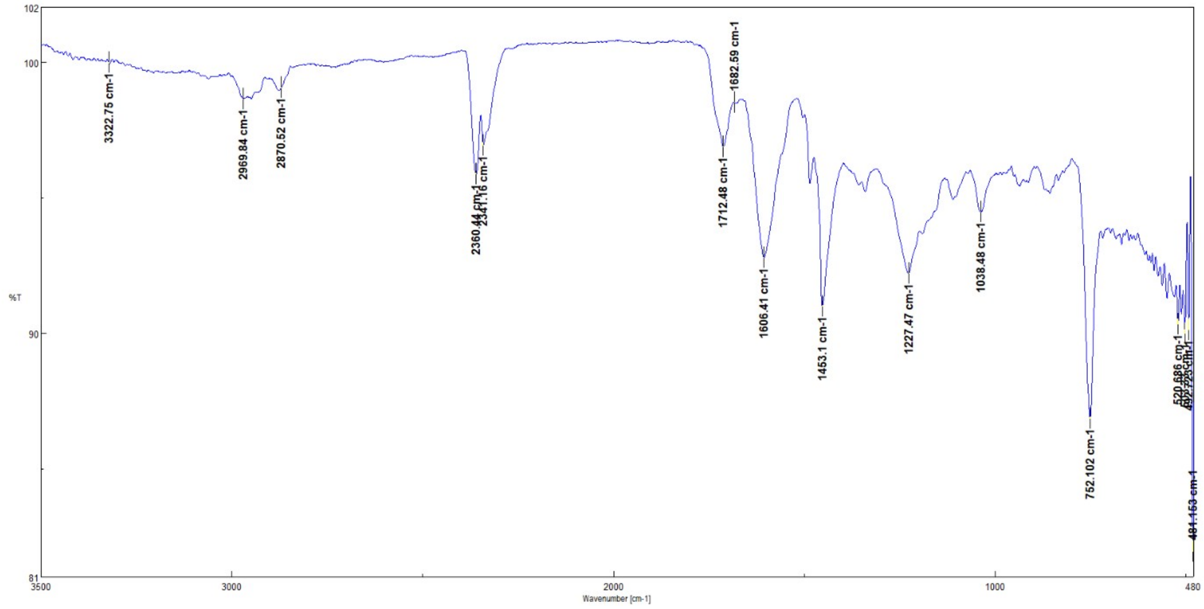

Fig. S54. FTIR Spectra of compound 7e

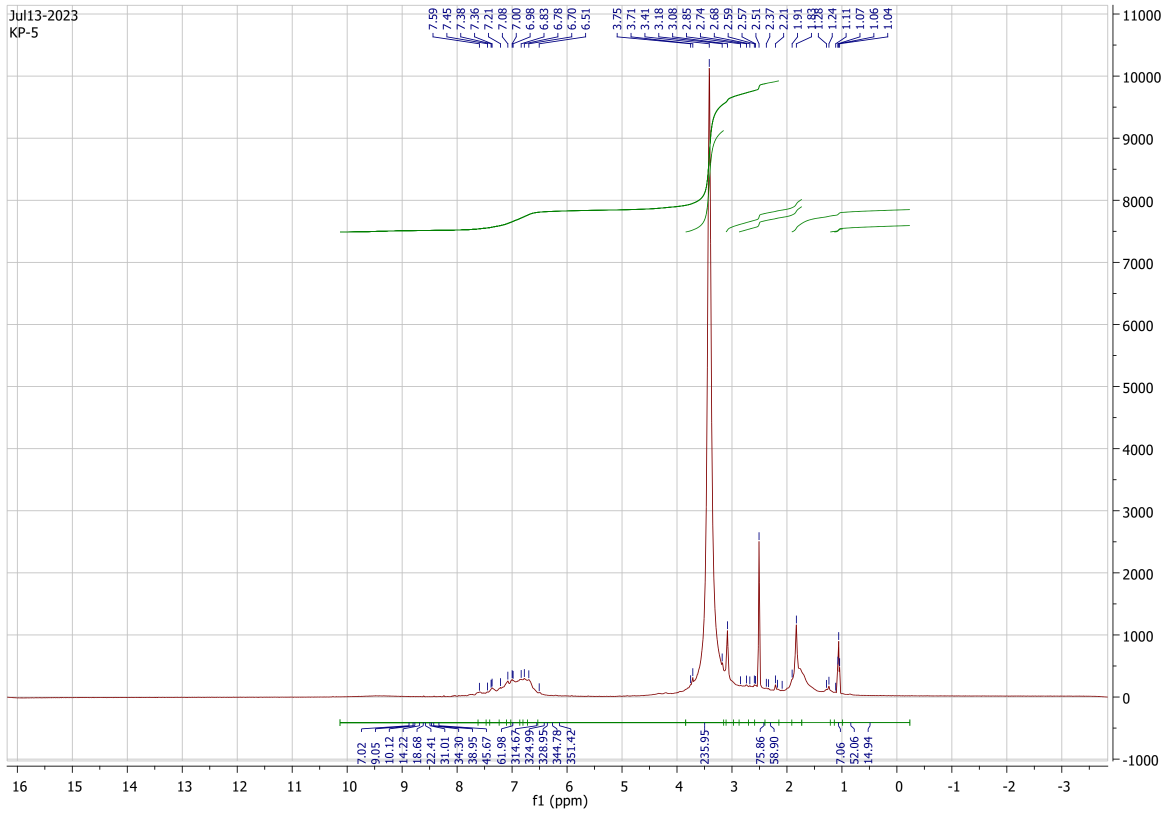

Fig. S55. ^{1}H NMR Spectra of compound 7e

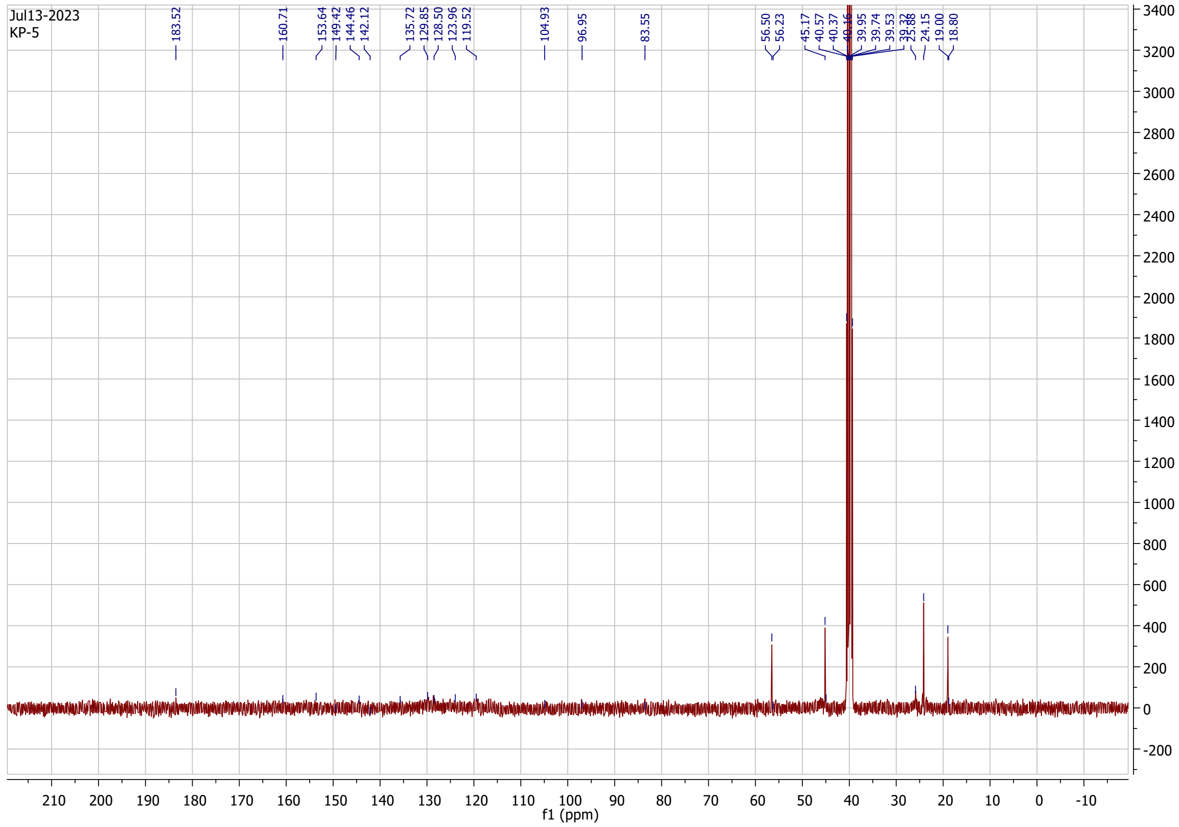

Fig. S56. ^{13}C NMR Spectra of compound 7e

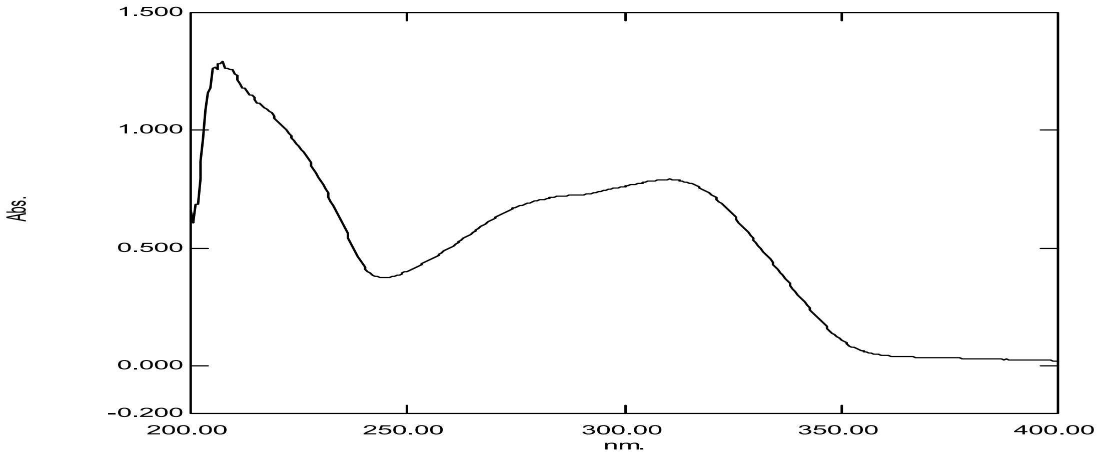

Fig. S57. UV-Visible spectra of compound 7f

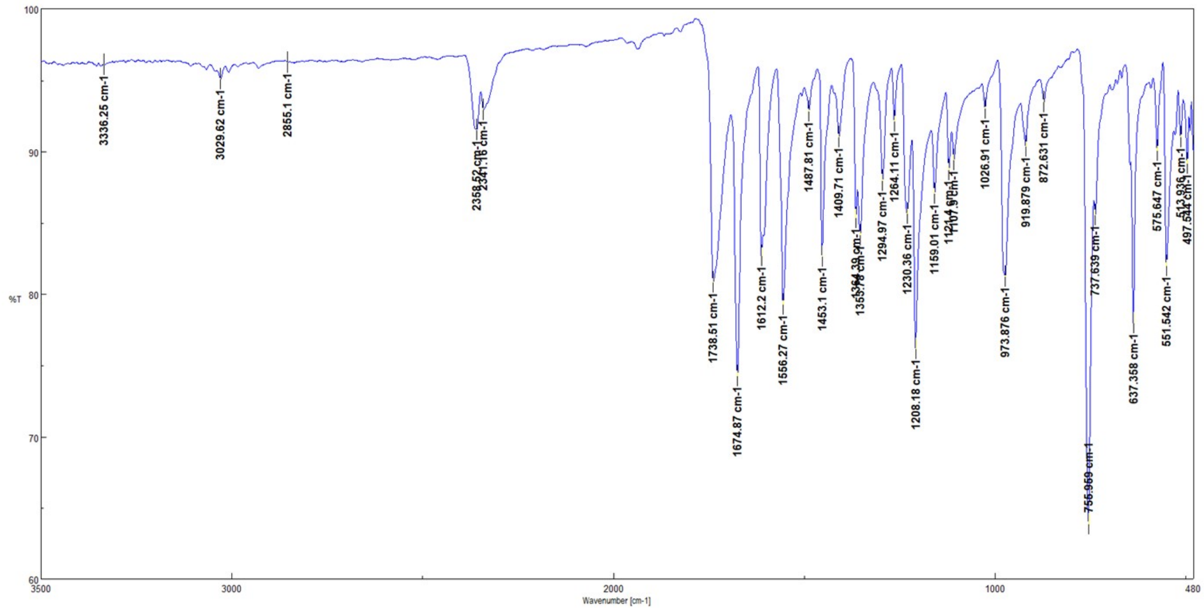

Fig. S58. FTIR Spectra of compound 7f

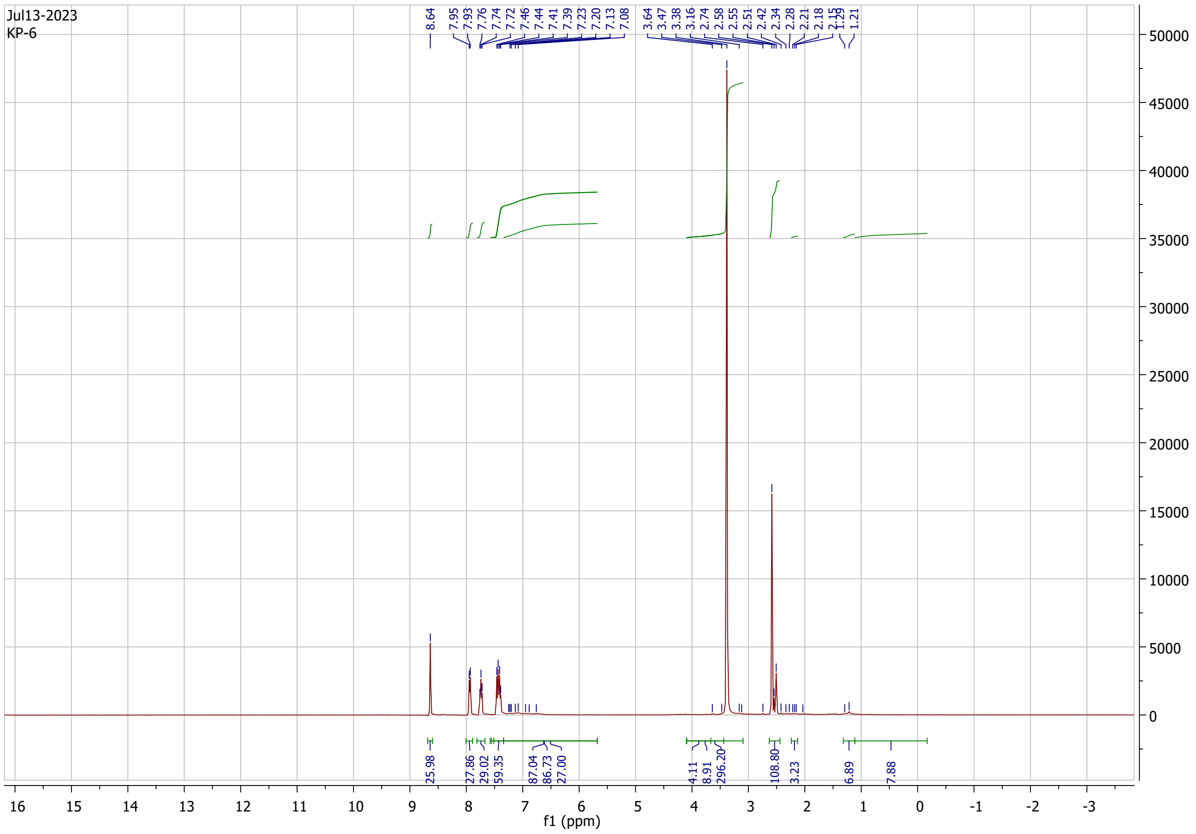

Fig. S59. ^{1}H NMR Spectra of compound 7f

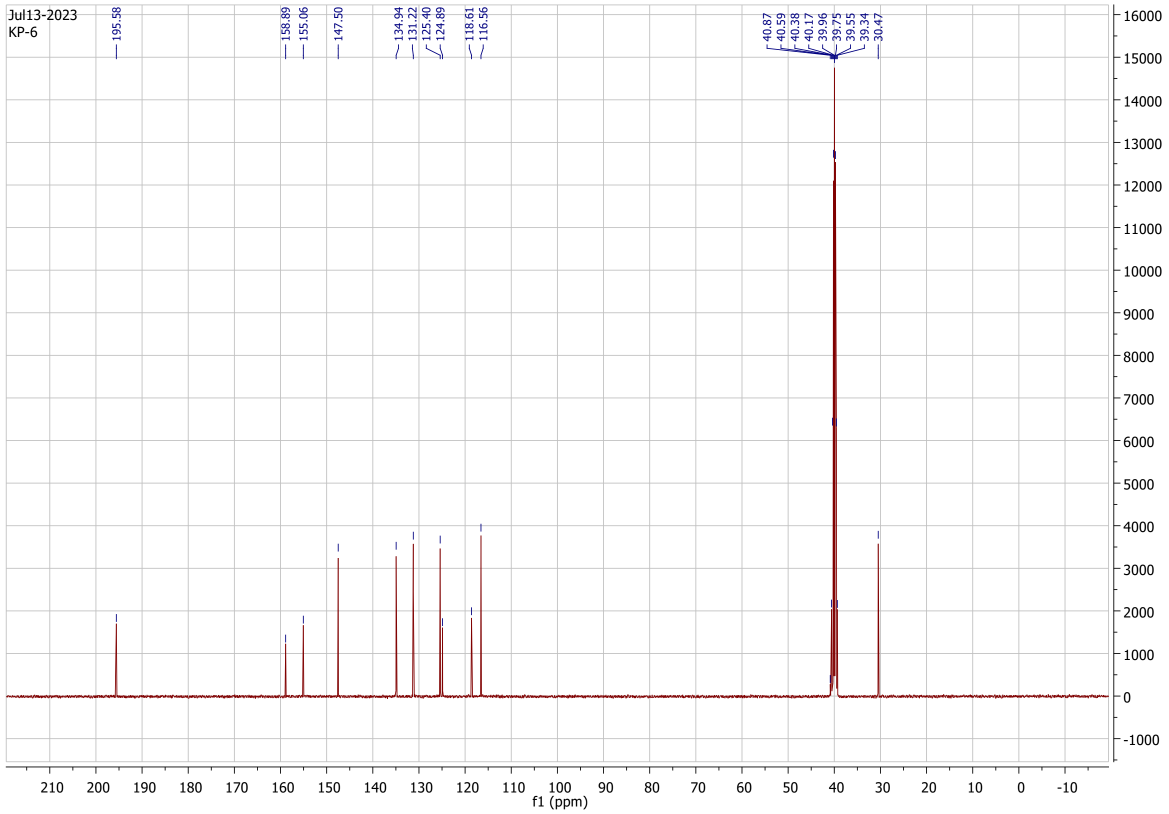

Fig. S60. ^{13}C NMR Spectra of compound 7f

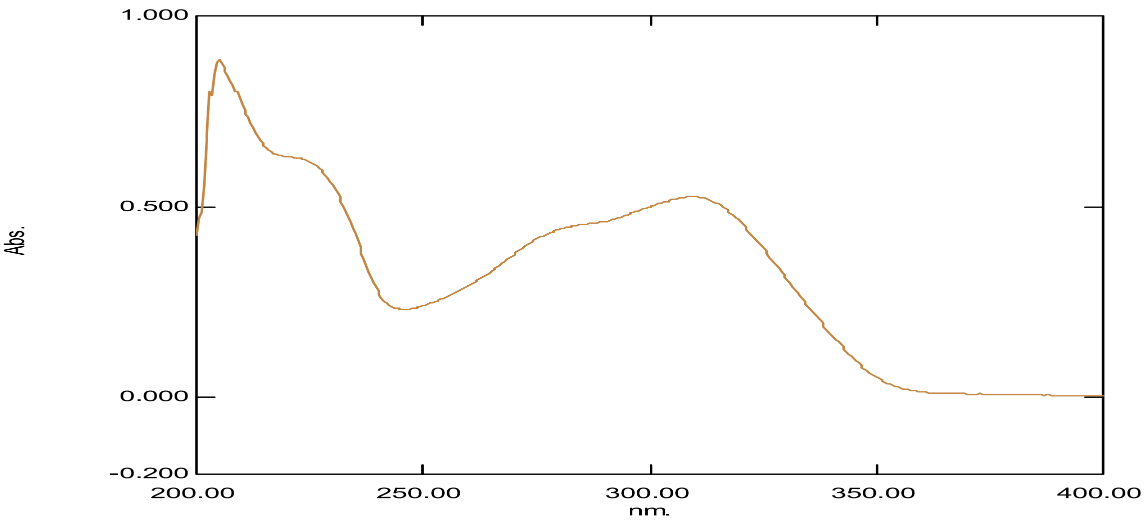

Fig. S61. UV-Visible spectra of compound 7g

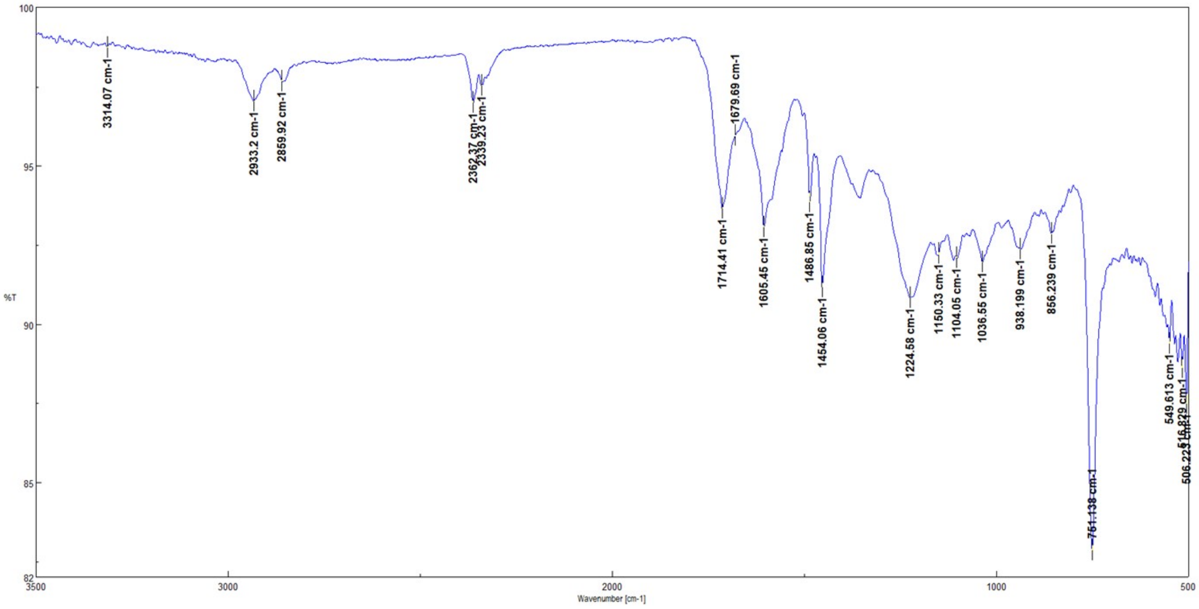

Fig. S62. FTIR Spectra of compound 7g

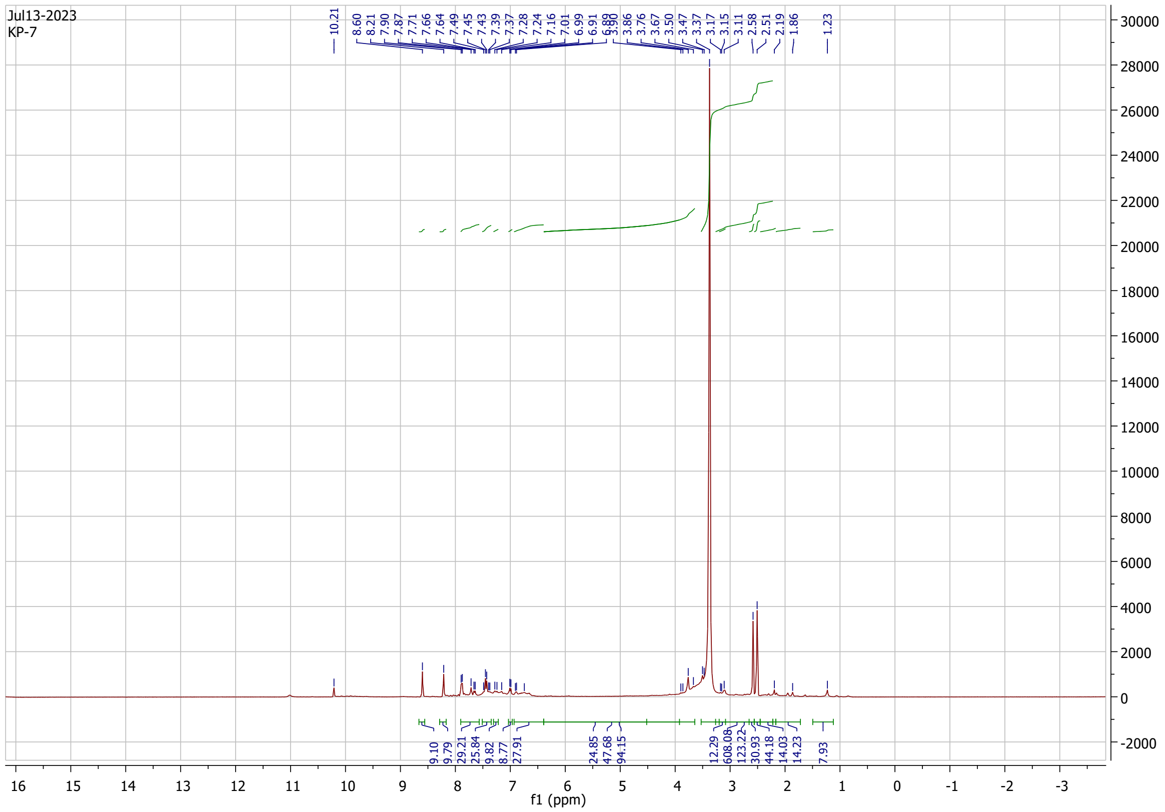

Fig. S63. ^{1}H NMR Spectra of compound 7g

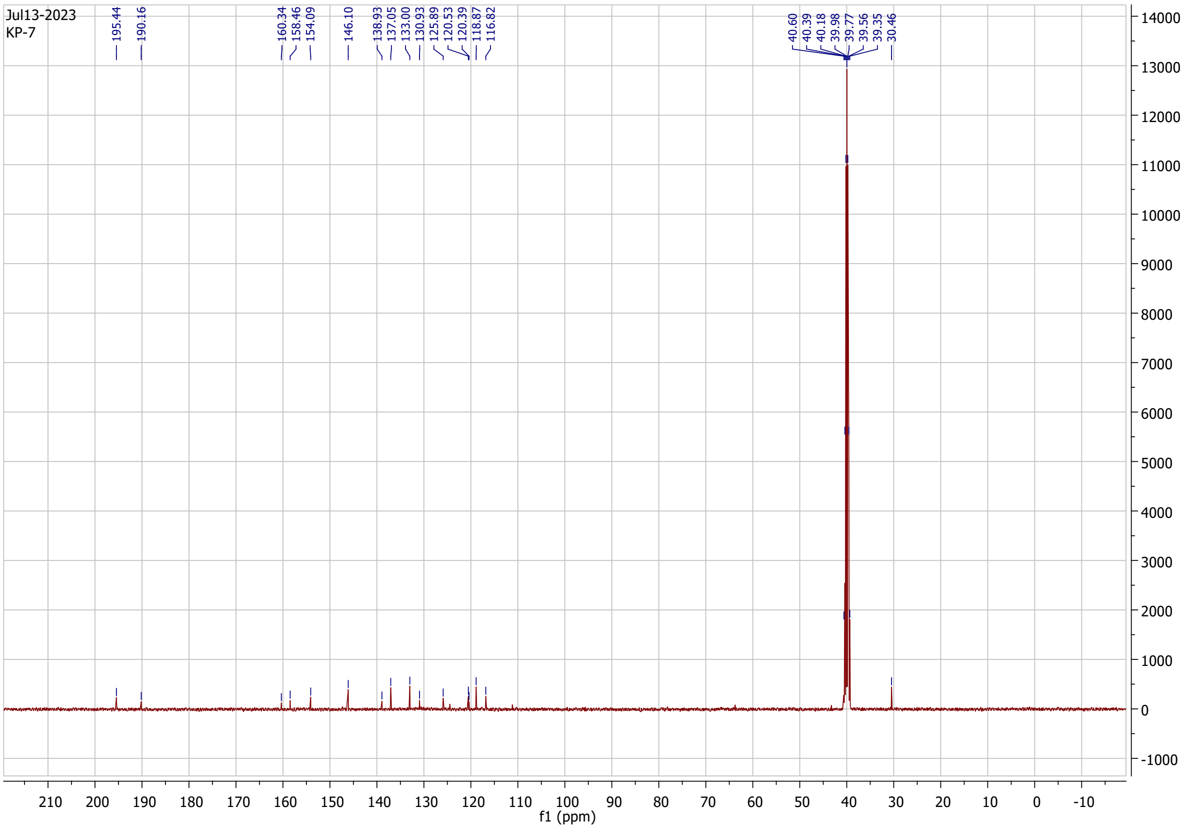

Fig. S64. ^{13}C NMR Spectra of compound 7g

Antimicrobial assessment

Zone of Inhibition of compounds 5d & 5f

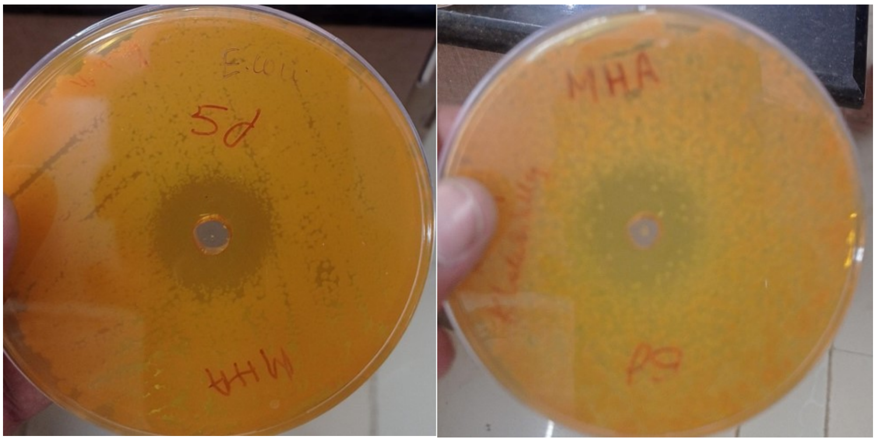

Fig. S65. Zone of Inhibition of compounds 5d & 5f

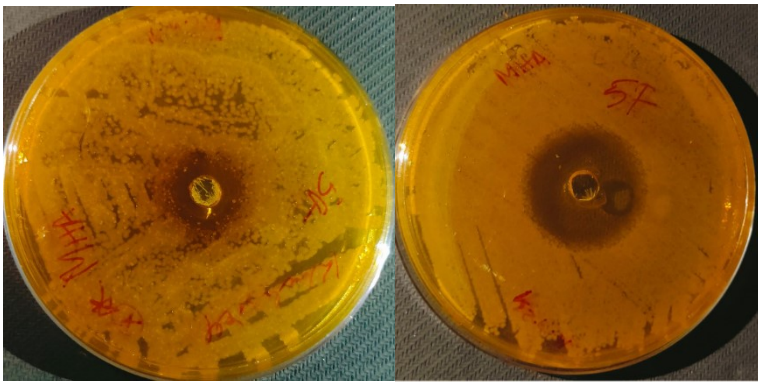

Fig. S66. Zone of Inhibition of compounds 5d & 5f

Serial dilution method

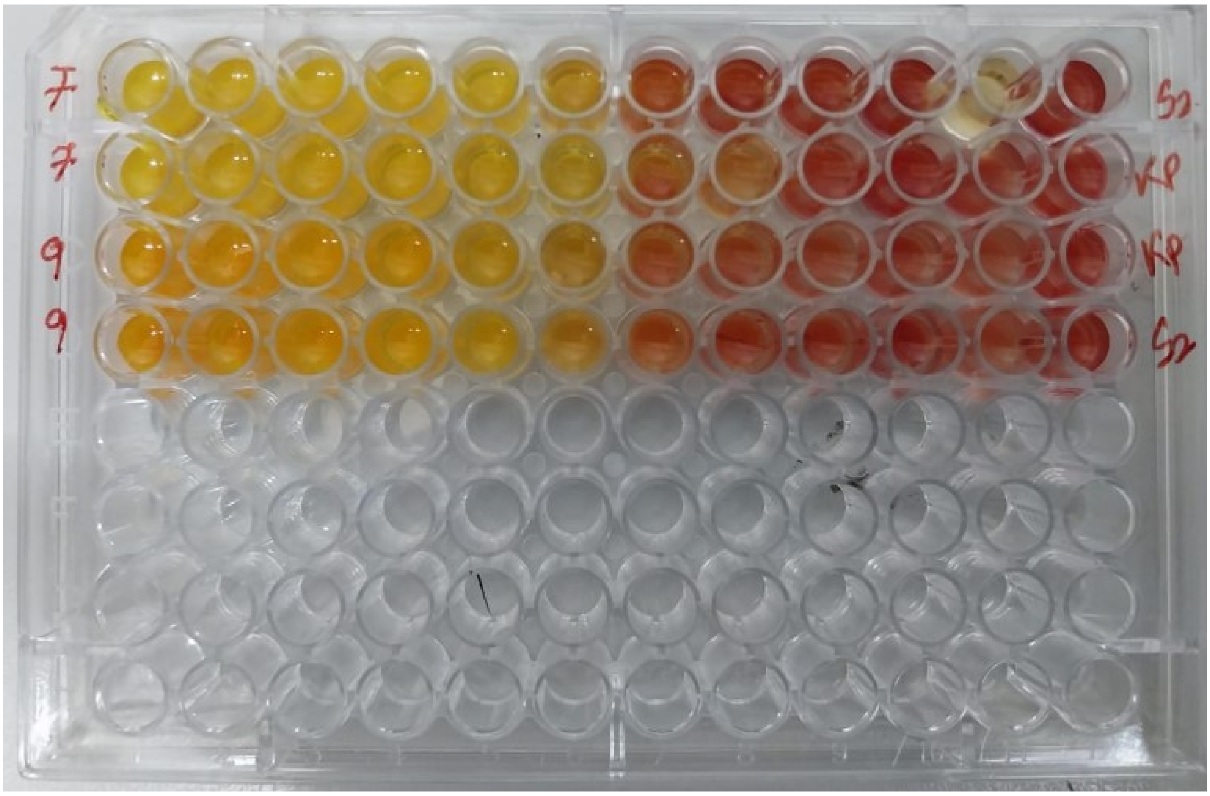

Fig. S67. Minimum Inhibitory Concentration of compounds 5d & 5f

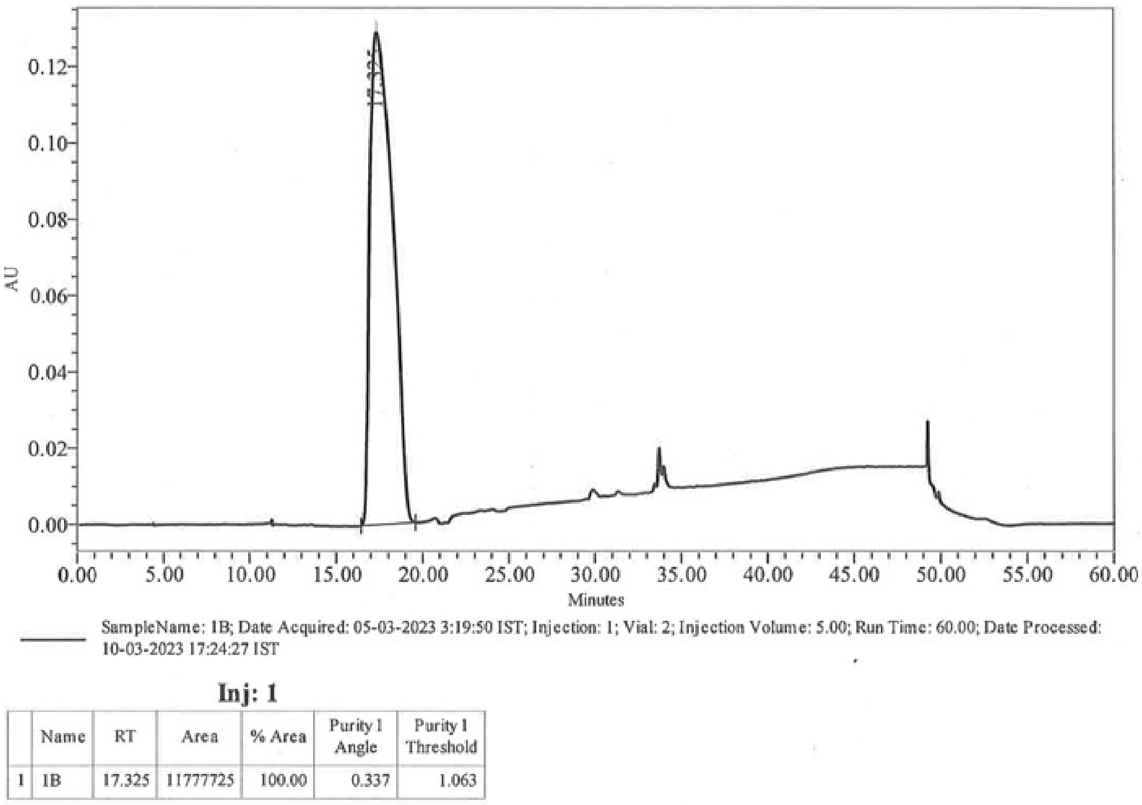

Inj: 1

	Name	RT	Area	% Area	Purity 1 Angle	Purity 1 Threshold
1	1B	17.325	11777725	100.00	0.337	1.063

Fig. S68. HPLC chromatogram of compound 5d
